# Hepatitis B and C in Europe: an update from the Global Burden of Disease Study 2019

**DOI:** 10.1016/S2468-2667(23)00149-4

**Published:** 2023-08-24

**Authors:** GBD 2019 Europe Hepatitis B & C Collaborators, Paolo Angelo Cortesi, Carla Fornari, Sara Conti, Ippazio Cosimo Antonazzo, Pietro Ferrara, Ayman Ahmed, Catalina Liliana Andrei, Tudorel Andrei, Anton A Artamonov, Maciej Banach, Carl Michael Baravelli, Till Winfried Bärnighausen, Akshaya Srikanth Bhagavathula, Nikolay Ivanovich Briko, Daniela Calina, Giulia Carreras, Sheng-Chia Chung, Mostafa Dianatinasab, Eleonora Dubljanin, Oyewole Christopher Durojaiye, Ifeanyi Jude Ezeonwumelu, Adeniyi Francis Fagbamigbe, Florian Fischer, Silvano Gallus, Ekaterina Vladimirovna Glushkova, Davide Golinelli, Giuseppe Gorini, Shoaib Hassan, Simon I Hay, Mihaela Hostiuc, Irena M Ilic, Milena D Ilic, Mihajlo Jakovljevic, Elham Jamshidi, Jacek Jerzy Jozwiak, Zubair Kabir, Joonas H Kauppila, Rovshan Khalilov, Moien AB Khan, Khaled Khatab, Ai Koyanagi, Carlo La Vecchia, Jeffrey V Lazarus, Caterina Ledda, Miriam Levi, Platon D Lopukhov, Joana A Loureiro, Philippa C Matthews, Alexios-Fotios A Mentis, Tomislav Mestrovic, Babak Moazen, Shafiu Mohammed, Lorenzo Monasta, Francesk Mulita, Christopher J L Murray, Ionut Negoi, Bogdan Oancea, Claudia Palladino, Jay Patel, Ionela-Roxana Petcu, Maarten J Postma, David Laith Rawaf, Salman Rawaf, Esperanza Romero-Rodríguez, Milena M Santric-Milicevic, Valentin Yurievich Skryabin, Anna Aleksandrovna Skryabina, Rafael Tabarés-Seisdedos, Mircea Tampa, Nuno Taveira, Arulmani Thiyagarajan, Marcos Roberto Tovani-Palone, Ronny Westerman, Mikhail Sergeevich Zastrozhin, Giampiero Mazzaglia, Lorenzo Giovanni Mantovani

## Abstract

**Background:**

In 2016, the World Health Assembly adopted the resolution to eliminate viral hepatitis by 2030. This study aims to provide an overview of the burdens of hepatitis B virus (HBV) and hepatitis C virus (HCV) in Europe and their changes from 2010 to 2019 using estimates from the Global Burden of Diseases, Injuries, and Risk Factors Study (GBD) 2019.

**Methods:**

We used GBD 2019 estimates of the burden associated with HBV-related and HCV-related diseases: acute hepatitis, cirrhosis and other chronic liver diseases, and liver cancer. We report total numbers and age-standardised rates per 100 000 for mortality, prevalence, incidence, and disability-adjusted life-years (DALYs) from 2010 to 2019. For each HBV-related and HCV-related disease and each measure, we analysed temporal changes and percentage changes for the 2010–19 period.

**Findings:**

In 2019, across all age groups, there were an estimated 2·08 million (95% uncertainty interval [UI] 1·66 to 2·54) incident cases of acute hepatitis B and 0·49 million (0·42 to 0·57) of hepatitis C in Europe. There were an estimated 8·24 million (7·56 to 8·88) prevalent cases of HBV-related cirrhosis and 11·87 million (9·77 to 14·41) of HCV-related cirrhosis, with 24·92 thousand (19·86 to 31·03) deaths due to HBV-related cirrhosis and 36·89 thousand (29·94 to 45·56) deaths due to HCV-related cirrhosis. Deaths were estimated at 9·00 thousand (6·88 to 11·62) due to HBV-related liver cancer and 23·07 thousand (18·95 to 27·31) due to HCV-related liver cancer. Between 2010 and 2019, the age-standardised incidence rate of acute hepatitis B decreased (–22·14% [95% UI –35·44 to –5·98]) as did its age-standardised mortality rate (–33·27% [–43·03 to –25·49]); the age-standardised prevalence rate (–20·60% [–22·09 to –19·10]) and mortality rate (–33·19% [–37·82 to –28·13]) of HBV-related cirrhosis also decreased in this time period. The age-standardised incidence rate of acute hepatitis C decreased by 3·24% (1·17 to 5·02) and its age-standardised mortality rate decreased by 35·73% (23·48 to 47·75) between 2010 and 2019; the age-standardised prevalence rate (–6·37% [–8·11 to –4·32]), incidence rate (–5·87% [–11·24 to –1·01]), and mortality rate (–11·11% [–16·54 to –5·53]) of HCV-related cirrhosis also decreased. No significant changes were observed in age-standardised rates of HBV-related and HCV-related liver cancer, although we observed a significant increase in numbers of cases of HCV-related liver cancer across all ages between 2010 and 2019 (16·41% [2·81 to 30·91] increase in prevalent cases). Substantial reductions in DALYs since 2010 were estimated for acute hepatitis B (–27·82% [–36·92 to –20·24]), acute hepatitis C (–27·07% [–15·97 to –39·34]), and HBV-related cirrhosis (–30·70% [–35·75 to –25·03]). A moderate reduction in DALYs was estimated for HCV-related cirrhosis (–6·19% [–0·19 to –12·57]). Only HCV-related liver cancer showed a significant increase in DALYs (10·37% [4·81–16·63]). Changes in age-standardised DALY rates closely resembled those observed for overall DALY counts, except for HCV-liver related cancer (–2·84% [–7·75 to 2·63]).

**Interpretation:**

Although decreases in some HBV-related and HCV-related diseases were estimated between 2010 and 2019, HBV-related and HCV-related diseases are still associated with a high burden, highlighting the need for more intensive and coordinated interventions within European countries to reach the goal of elimination by 2030.

**Funding:**

Bill & Melinda Gates Foundation.

## Introduction

Hepatitis B virus (HBV) and hepatitis C virus (HCV) can cause acute and chronic infections and are major causes of cirrhosis, liver cancer, liver transplantation, and liver-related death worldwide.^1^ These viruses cause high economic and health burdens due to their hepatic and extrahepatic effects.^2^, ^3^, ^4^ The availability of reliable diagnostic assays and cost-effective interventions has created conditions in which the elimination of HCV and HBV is a feasible objective.^2^, ^5^, ^6^, ^7^, ^8^ Available interventions include universal HBV immunisation programmes, screening for HBV and HCV and linkage to care, prevention of mother-to-child transmission, promotion of safe injection practices, stringent infection-control programmes, and antiviral treatments for people with HBV and HCV infections.^5^ Direct-acting antiviral treatments for HCV infection, introduced in 2013 in the USA and authorised by the European Medicines Agency for marketing in 2014,^9^, ^10^ have changed the approach to HCV management and have enabled public health organisations and agencies to target the worldwide elimination of the burden associated with the virus.^5^

**Research in context**

**Evidence before this study**

Comprehensive and timely estimation of hepatitis B virus (HBV) and hepatitis C virus (HCV) prevalence, incidence, mortality, and disability-adjusted life-years (DALYs) is crucial to assess disease burden, develop policies and programmes, and evaluate progress towards the WHO target of elimination of viral hepatitis by 2030. Several research groups have produced estimates of various measures of HBV and HCV burden. A search of institutional websites for recent publications from the various research groups and institutions was conducted, and we identified global studies during our literature search of PubMed and grey literature. The search terms (“hepatitis C” OR “hepatitis B” AND “prevalence” OR “mortality” AND “country/territory”) were used to identify articles in all languages published between Jan 1, 2014, and May 31, 2022. The Center for Disease Analysis Foundation produced estimates on prevalence for 120 locations in 2016 using a compartmental model, reporting an HBsAg infection prevalence of 1·7% (95% UI 1·3–2·1) in central Europe, 1·5% (0·8–1·8) in eastern Europe, and 0·6% (0·4–0·8) in western Europe. The Foundation also estimated HCV burden for 110 locations in 2020, reporting 0·9 million (0·9–1·2) people with viraemic-HCV infection in central Europe, 6·1 million (4·9–6·6) in eastern Europe, and 1·4 (1·3–1·7) in western Europe. Based on forecasts, the global annual number of new (incident) chronic HCV infections in the total population was expected to remain relatively constant from 2020 to 2030. By 2030, end-stage outcomes (liver-related deaths, hepatocellular carcinoma, and decompensated cirrhosis) in adults were expected to increase by 14–17%. Schweitzer and colleagues generated broad time-period and all-age estimates for HBsAg seroprevalence for 161 countries, reporting an HBsAg seroprevalence of 2·06% (2·06–2·06) and a number of people living with chronic HBV of 18·48 million in the general population of the WHO European Region in 2010. WHO reported that 820 000 people died from HBV infection-related causes and 290 000 from HCV infection-related causes in 2019. The European Association for the Study of the Liver produced a second overview (following their 2013 report) of the burden of liver disease across Europe in 2017, reporting difficulty in the interpretation of hepatitis B and C data due to differences in testing and screening practices, lack of availability of good quality, timely, and nationally representative data, and limited general population level data. Estimates of the burden of acute hepatitis B, acute hepatitis C, cirrhosis due to HBV and HCV, and liver cancer due to HBV and HCV have been included in the comprehensive report of the Global Burden of Diseases, Injuries, and Risk Factors Study (GBD) and the worldwide burden of HBV based on GBD 2019 estimates has also been reported. However, a collective, detailed analysis of the burden of HBV and HCV in Europe has not, to our knowledge, been reported previously.

**Added value of this study**

GBD provides updated, comparable, detailed, and internally consistent estimates of HBV and HCV incidence, prevalence, mortality, and DALYs for all age groups, years (1990–2019), and locations. This publication is the first comprehensive report on estimates of the HBV and HCV burden in Europe from GBD 2019, which includes new data sources on HBsAg seroprevalence to improve estimations and account for HBV vaccination efforts—crucial drivers of the reduction in chronic HBV infection burden. GBD 2019 methods were also improved for redistribution of vaguely characterised codes in vital registration data to acute hepatitis, cirrhosis, and liver cancer. The study provides detailed information on changes in the HBV and HCV burden from 2010 to 2019 in Europe, highlighting areas with high burden, areas with small reductions in burden, and areas in need of improvements in public health interventions to reach WHO elimination goals. In our study, the burden of HBV and HCV remained high in Europe in 2019. In 2019, central and eastern Europe had higher age-standardised mortality rates for HBV-related cirrhosis and liver cancer than western Europe**.** The age-standardised prevalence and mortality rates for HCV-related cirrhosis were highest in eastern Europe, while the prevalence and mortality rates of HCV-related liver cancer were highest in western Europe. Between 2010 and 2019, the burden of cirrhosis due to HBV and HCV decreased. HBV-related cirrhosis and acute hepatitis B showed the most substantial reductions among all HBV-related and HCV-related diseases assessed. No changes were observed in age-standardised rates of liver cancer incidence, prevalence, mortality, and DALYs from 2010 to 2019, whereas we observed increases in all-age incidence, prevalence, mortality, and DALYs.

**Implications of all the available evidence**

Our findings highlight considerable and persistent burdens of HBV and HCV in Europe, showing that the ambitious goal of elimination by 2030 is far from being achieved. Some well structured frameworks for hepatitis prevention and control are available and have been demonstrated in some countries. However, action plans or strategies for hepatitis prevention and control, as well as funding for implementation, are still inadequate or absent in many countries. Hepatitis elimination needs greater commitment from governments, health-care systems, national and international institutions, civil society, and donors. Investment in prevention, detection, and treatment of hepatitis is likely to contribute to reductions in liver-related deaths due to HBV and HCV, incidence of advanced liver disease complications, and related management costs. Hepatitis elimination responses worldwide require information from enhanced monitoring and evaluation systems.

In 2016, the World Health Assembly adopted a resolution to eliminate viral hepatitis as a major public health threat, and WHO subsequently published a policy document on the possible global health strategies and relative budgets required to reach the elimination goal.^2^

The burden of HCV and HBV has been assessed in previous studies at the global and European levels. In an update to their 2016 analysis,^11^ the Center for Disease Analysis Foundation recently published estimates of HBV burden for 170 locations in 2022 using a compartmental model, reporting an HBV infection prevalence of 1·5% (95% uncertainty interval [UI] 1·2–1·9) in central Europe (1·7 million [95% UI 1·4–2·2] HbsAg-positive individuals), 1·4% (0·8–1·7) in eastern Europe (2·9 million [1·6–3·4]), and 0·5% (0·3–0·7) in western Europe (2·0 million [1·4–2·8]).^12^ The Foundation also estimated HCV infection burden for 110 locations in 2020, reporting 0·9 million (95% UI 0·9–1·2) people with viraemic-HCV infection in central Europe, 6·1 million (4·9–6·6) in eastern Europe, and 1·4 million (1·3–1·7) in western Europe.^13^ Based on forecasts, the global annual number of incident chronic HCV infections in the total population was not expected to change substantially from 2020 to 2030, while end-stage outcomes (liver-related deaths, hepatocellular carcinoma, and decompensated cirrhosis) in the adult population were expected to increase by 14–17%. Schweitzer and colleagues^14^ generated broad time-period and all-age estimates of HBsAg positivity for 161 countries in 2010, reporting an HBsAg seroprevalence of 2·06% (95% CI 2·06–2·06) and a number of people living with chronic HBV of 18·5 million in the general population of the WHO European region. In a 2021 global progress report, WHO reported for the European region 19 000 (95% UI 9400–38 000) new HBV infections, 300 000 (240 000–320 000) new HCV infections, 43 000 (34 000–51 000) for HBV-related deaths, and 64 000 (39 000–72 000) for HCV-related deaths in 2019.^15^ The European Association for the Study of the Liver produced a second overview on the burden of liver disease across Europe in 2017,^16^ reporting difficulty in the interpretation of hepatitis B and C data due to differences in testing and screening practices, lack of availability of good quality, timely, and nationally representative data and insufficient general population-level data. Estimates of the burden of acute hepatitis, cirrhosis, and liver cancer due to HBV and HCV have been included in the comprehensive report of the Global Burden of Diseases, Injuries, and Risk Factors (GBD) study and the worldwide burden of HBV based on GBD 2019 estimates has also been reported. By contrast, a collective, detailed analysis of all HBV-related and HCV-related burden for Europe has not been reported.

The GBD approach provides an opportunity to investigate the burdens associated with HBV and HCV infections in Europe.^17^, ^18^ Using GBD 2019 estimates, we aimed to evaluate the incidence and prevalence of HBV-related and HCV-related diseases and their associated mortality and disability-adjusted life-years (DALYs) in Europe from 2010 to 2019 to elucidate variations in the past decade, when important treatment innovations have become available.

## Methods

### Overview

This work is based on data from GBD 2019, which estimated disease burden for 369 diseases and injuries in 204 countries from 1990 to 2019. A summary of the methods and complete report of results for all diseases and injuries have been published previously,^17^ and methods are also detailed in the GBD protocol.

The estimation approach used in GBD 2019^17^, ^18^ builds on the approach used in previous rounds of GBD studies, but estimates for the whole time-series were updated with methodological enhancements and new data. Several regression tools have been built for GBD and have been described previously, including the Causes of Death Ensemble model (CODEm) detailed by Foreman and colleagues^19^ and the Bayesian disease modelling meta-regression system (DisMod-MR) described by Abraham and colleagues^20^ (updates for GBD 2019 were detailed previously).^17^ GBD is compliant with the Guidelines for Accurate and Transparent Health Estimates Reporting (GATHER Statement).^21^

Methodological details for estimating burden related to HBV have been summarised in a previous report on the worldwide burden of HBV.^22^ A standalone report of HCV-related burden has not been previously published, but methods underlying these estimates were described in the GBD 2019 capstone publication.^17^

For all diseases, the GBD approach estimates the associated burden in terms of incidence, prevalence, mortality, and DALYs. All estimates computed in GBD were done 1000 times at the draw level to account for uncertainty from input data, data adjustments, and model selection. The bounds of the 95% uncertainty intervals (UIs) were taken as the 25th and 975th of the 1000 ordered draws. Age-standardisation was done with the direct method, and population estimates for all combinations of age group, sex, location, and year were taken from GBD demographic estimates.^18^

GBD diseases and injuries are organised into a levelled cause hierarchy (appendix p 2).^17^ We extracted GBD estimates related to Level 4 diseases associated with HBV and HCV: acute hepatitis, cirrhosis and other chronic liver diseases (collectively referred to hereafter as cirrhosis), and liver cancer (appendix p 2). We included estimates for Europe from 2010 to 2019.

### Mortality modelling

The GBD study modelled acute hepatitis mortality, irrespective of virus type, using data from the GBD cause of death database and a CODEm.^17^ In the GBD cause of death database, data are standardised, and unspecified or non-underlying causes of death are redistributed on the basis of evidence from a subset of data with multiple diagnostic codes. Unspecified deaths associated with acute hepatitis B were redistributed to mostly cirrhosis and other chronic liver diseases, and a small proportion to acute hepatitis B.^17^

Virus-specific acute hepatitis models used cause of death data only from countries with high-performing vital registration systems (4-star and 5-star cause of death data reported in 34 of 44 countries included), in four separate CODEms for acute hepatitis A, B, C, and E.^17^ Virus-specific deaths were then rescaled to fit within the parent envelope in CoDCorrect, the process by which all cause-specific deaths are scaled to add up to all-cause mortality.^17^ Cirrhosis mortality was modelled with use of data in the cause of death database and proportions of cirrhosis cases attributed to alcohol, HBV, HCV, non-alcoholic steatohepatitis (NASH), and other causes reported in case-series studies. In GBD 2019, 12 new case-series studies were added and cirrhosis due to NASH or non-alcoholic fatty liver disease (NAFLD) was considered as a new category and no longer included in the other chronic liver diseases category.^17^ Total cirrhosis mortality was modelled with a CODEm.^22^ Proportions of cirrhosis due to each of five aetiologies (alcohol, HBV, HCV, NASH, and other) were modelled with DisMod-MR, rescaled to 1, and used to split total estimates from the CODEm.^22^

Liver cancer mortality estimates were obtained from the GBD cause of death database, which includes multiple sources of cancer mortality data and cancer registry data.^17^ The cancer registry mortality data stem from incidence data transformed to mortality estimates using mortality-to-incidence ratios. Liver cancer mortality was split into cases due to specific aetiologies using data from published case-series (available through the GBD 2019 Data Input Sources Tool), which were used as input for five separate DisMod-MR models determining the proportion of liver cancers due to each aetiology (alcohol, HBV, HCV, NASH, and other). These proportions were then scaled to 1, and the parent CODEm estimates were multiplied by corresponding scaled proportions.

### Morbidity modelling

GBD 2019 estimated HBsAg seroprevalence and seroincidence with a multistep approach that first estimated these quantities in a DisMod-MR model using only data from unvaccinated cohorts to produce counterfactual seroprevalence estimates, and then deducted seroprevalent cases based on estimates of HBV vaccine coverage and efficacy.^22^ GBD also estimated the prevalence of HCV viraemia in a multistep manner, first using DisMod-MR to estimate the seroincidence and seroprevalence of anti-HCV antibodies, then converting these to estimates of viraemia using the results of a meta-analysis of studies that reported both measures with the same samples, and then deducting cases treated in national treatment programmes.^17^

GBD defined acute hepatitis as the period corresponding to initial infection with HBV or HCV, regardless of symptoms. Incidence from modelling HBsAg seroprevalence was regarded as the incidence of chronic carriage, and was divided by the age-specific probability of infection resulting in carriage previously published by Edmunds and colleagues^23^ to obtain the incidence of acute hepatitis B. Incidence from modelling anti-HCV antibody seroprevalence was regarded as the incidence of infection, regardless of whether an individual remained viraemic, and was treated as the incidence of acute hepatitis C. For acute hepatitis B and acute hepatitis C, prevalence was estimated as the product of the incidence of infection and an assumed duration of acute infection of 6 weeks, based on clinical expert opinion and published work.^17^

GBD modelled the prevalence of total cirrhosis and decompensated cirrhosis using hospital discharge claims data and DisMod-MR.^22^ The same aetiological proportion models used to split mortality estimates, described above, were also used to split incidence and prevalence estimates for total and decompensated cirrhosis.^22^

The incidence of liver cancer was directly estimated from cancer mortality with use of estimated mortality-to-incidence ratios. After transforming final GBD liver cancer mortality estimates to incidence estimates, incidence was combined with annual relative survival estimates from 1 year to 10 years to compute prevalence.^17^ Splitting into aetiological fractions was done using proportions computed with DisMod-MR models. Prevalence was estimated by applying stage-specific survival information from Surveillance, Epidemiology, and End Results Program data to incidence estimates, as previously described.^17^ People with liver cancer who survived past 10 years were considered cured and no longer contributed to prevalent case estimation.^17^

### Disability estimation

DALYs measure the health loss due to mortality or disability as the sum of years of life lost due to premature mortality and years lived with disability. Years lived with disability are calculated as the prevalence of specific, non-fatal outcomes of a disease for a specific year, age group, sex, and location, multiplied by a disability weight that ranges between 0 (equivalent to perfect health) and 1 (equivalent to death). Disability weight estimation for GBD has been described previously.^24^

In GBD 2019, prevalent cases of acute hepatitis were divided by severity and assigned disability weights as follows: asymptomatic (disability weight 0 [range 0–0]), symptomatic moderate (0·051 [0·032–0·074]), and symptomatic severe (0·133 [0·088–0·190]).^17^ A disability weight of 0 was assigned to compensated cirrhosis cases because of its asymptomatic nature. Cases of decompensated cirrhosis were divided according to the presence and severity of accompanying anaemia and assigned disability weights as follows: no anaemia (0·178 [0·113–0·243]), mild anaemia (0·181 [0·116–0·246]), moderate anaemia (0·220 [0·146–0·295]), and severe anaemia (0·300 [0·202–0·397]).^22^ Prevalent cases of liver cancer were split into four sequelae (ie, non-fatal outcomes) with disability weights: diagnosis and primary treatment (0·288 [0·193–0·399]), controlled phase (0·049 [0·031–0·072]), metastatic phase (0·451 [0·307–0·600]), and terminal phase (0·540 [0·377–0·687]).^22^

### Measures

We report total numbers (counts) and age-standardised rates per 100 000 population for mortality, incidence, and DALYs, and age-standardised prevalence per 100 000 (referred to as prevalence rate) of HBV-related and HCV-related diseases in Europe from 2010 to 2019. We included all countries of the WHO European region as defined by GBD (appendix p 8). All measures are reported with 95% UIs as described above.

For each HBV-related and HCV-related disease and each measure, we present mean estimates for 2010 and 2019 and the percentage change between those two timepoints, each with 95% UIs. Percentage change was calculated as the difference between the 2019 and 2010 estimates, divided by the 2010 estimate, calculated at the draw level to propagate uncertainty. These estimates are presented for the whole of Europe, stratified by European area: eastern, central, western (appendix p 8), and for each European country. Means and UIs of these quantities are also presented annually from 2010 to 2019 in graphical form for all of Europe and stratified by European area, and means and percentage change are presented at the country level in maps.

### Role of the funding source

The funder of this study had no role in study design, data collection, data analysis, data interpretation, or the writing of the report.

## Results

### Burden of HBV in Europe

The dynamics differed for the three HBV-related diseases (acute HBV, HBV-associated cirrhosis, and HBV-associated liver cancer) between 2010 and 2019 (table 1). The age-standardised incidence rate of acute hepatitis B decreased by 22·14% (95% UI 5·98 to 35·44) and the mortality rate due to acute hepatitis B by 33·27% (25·49 to 43·03) from 2010 to 2019. HBV-related cirrhosis was characterised by a high prevalence (8·24 million [95% UI 7·56 to 8·88] cases), and accounted for 24·92 thousand (19·86 to 31·03) deaths in 2019, but the age-standardised prevalence rate (–20·60% [–22·09 to –19·10]) and mortality rate (–33·19% [–37·82 to –28·13]) of HBV-related cirrhosis decreased from 2010 to 2019 (table 1). 14·97 thousand (95% UI 11·51 to 19·47) prevalent cases, 10·17 thousand (7·79 to 13·40) incident cases, and 9·00 thousand (6·88 to 11·62) deaths were attributable to HBV-related liver cancer in 2019, with no significant changes in age-standardised rates observed between 2010 and 2019 (table 1).

**Table 1:** Prevalence, incidence, mortality, and DALYs attributable to HBV infection, by calendar year, 2010–19

	**Number, thousands (95% UI)**				**Age-standardised rate per 100 000 population (95% UI)**			
	Prevalent cases	Incident cases	Deaths	DALYs	Prevalence rate	Incidence rate	Mortality rate	DALY rate
**Acute hepatitis B**								
2010	290·17 (237·67 to 351·55)	2514·80 (2059·84 to 3046·80)	0·58 (0·52 to 0·62)	27·63 (24·81 to 31·10)	33·82 (27·94 to 40·89)	293·13 (242·17 to 354·36)	0·06 (0·05 to 0·06)	3·32 (2·98 to 3·69)
2011	286·74 (238·15 to 344·37)	2485·10 (2063·99 to 2984·56)	0·56 (0·50 to 0·60)	26·62 (23·83 to 30·03)	33·31 (27·87 to 39·68)	288·67 (241·52 to 343·92)	0·06 (0·05 to 0·06)	3·18 (2·84 to 3·55)
2012	281·74 (233·94 to 337·29)	2441·77 (2027·48 to 2923·15)	0·53 (0·47 to 0·57)	25·40 (22·76 to 28·76)	32·54 (27·21 to 38·90)	281·99 (235·81 to 337·13)	0·05 (0·05 to 0·06)	3·03 (2·72 to 3·40)
2013	276·23 (229·40 to 329·20)	2393·96 (1988·14 to 2853·04)	0·48 (0·43 to 0·52)	23·47 (20·94 to 26·64)	31·67 (26·43 to 37·70)	274·48 (229·10 to 326·76)	0·05 (0·04 to 0·05)	2·80 (2·50 to 3·16)
2014	270·92 (222·57 to 323·63)	2348·01 (1928·90 to 2804·77)	0·45 (0·41 to 0·50)	22·79 (20·28 to 26·04)	30·84 (25·63 to 36·87)	267·29 (222·14 to 319·53)	0·04 (0·04 to 0·05)	2·72 (2·43 to 3·06)
2015	266·58 (219·18 to 325·56)	2310·37 (1899·56 to 2821·50)	0·42 (0·38 to 0·48)	21·60 (19·11 to 24·75)	30·19 (24·97 to 36·78)	261·67 (216·41 to 318·80)	0·04 (0·04 to 0·05)	2·57 (2·28 to 2·93)
2016	258·45 (213·20 to 305·63)	2239·86 (1847·71 to 2648·80)	0·41 (0·36 to 0·45)	20·71 (18·13 to 23·60)	29·04 (24·36 to 34·22)	251·70 (211·16 to 296·59)	0·04 (0·04 to 0·04)	2·45 (2·15 to 2·79)
2017	250·62 (202·29 to 305·64)	2172·00 (1753·14 to 2648·89)	0·41 (0·36 to 0·46)	20·43 (17·55 to 23·44)	27·92 (22·83 to 33·79)	242·01 (197·83 to 292·84)	0·04 (0·03 to 0·04)	2·38 (2·04 to 2·74)
2018	245·79 (201·81 to 291·61)	2130·16 (1748·98 to 2527·27)	0·41 (0·36 to 0·46)	20·22 (17·41 to 23·23)	27·23 (22·67 to 32·06)	235·98 (196·44 to 277·81)	0·04 (0·03 to 0·04)	2·35 (2·01 to 2·70)
2019	239·63 (191·92 to 292·66)	2076·80 (1663·32 to 2536·38)	0·41 (0·35 to 0·46)	19·95 (17·01 to 23·06)	26·33 (21·40 to 32·13)	228·23 (185·45 to 278·50)	0·04 (0·03 to 0·04)	2·31 (1·95 to 2·66)
% change, 2010 to 2019	−17·42% (−31·39 to −1·41)	−17·42% (−31·39 to −1·41)	−29·24% (−38·85 to −20·98)	−27·82% (−36·92 to −20·24)	−22·14% (−35·44 to −5·98)	−22·14% (−35·44 to −5·98)	−33·27% (−43·03 to −25·49)	−30·41% (−39·46 to −22·67)
**HBV-related cirrhosis and other chronic liver diseases**								
2010	9510·61 (8695·11 to 10 249·06)	38·36 (28·59 to 50·34)	33·91 (27·93 to 40·88)	1116·94 (917·36 to 1348·05)	1050·10 (953·35 to 1138·16)	4·02 (2·98 to 5·35)	2·89 (2·38 to 3·48)	101·57 (83·23 to 122·00)
2011	9397·52 (8614·60 to 10 120·92)	37·46 (27·92 to 49·23)	31·71 (26·01 to 38·36)	1031·72 (846·02 to 1248·20)	1030·56 (937·44 to 1117·98)	3·92 (2·90 to 5·20)	2·66 (2·19 to 3·22)	92·83 (76·08 to 112·10)
2012	9226·84 (8465·15 to 9930·49)	36·00 (26·82 to 47·24)	29·79 (24·34 to 36·25)	957·92 (782·19 to 1163·95)	1002·46 (913·56 to 1085·35)	3·75 (2·77 to 4·99)	2·47 (2·02 to 3·00)	85·37 (69·74 to 103·45)
2013	9043·76 (8327·41 to 9735·43)	34·34 (25·59 to 44·98)	27·66 (22·51 to 33·96)	881·07 (717·85 to 1074·59)	971·97 (887·78 to 1051·25)	3·56 (2·63 to 4·71)	2·26 (1·85 to 2·76)	77·89 (63·31 to 94·85)
2014	8883·77 (8180·84 to 9566·85)	32·85 (24·53 to 42·93)	26·11 (21·02 to 32·26)	823·27 (667·53 to 1005·41)	944·54 (865·02 to 1020·77)	3·38 (2·50 to 4·47)	2·11 (1·71 to 2·58)	72·15 (58·41 to 88·02)
2015	8783·38 (8078·68 to 9472·47)	31·91 (23·74 to 41·69)	26·08 (20·96 to 32·01)	821·40 (667·96 to 999·82)	925·93 (849·66 to 1001·27)	3·27 (2·41 to 4·32)	2·09 (1·70 to 2·55)	71·65 (58·03 to 87·06)
2016	8667·98 (7989·54 to 9363·36)	31·19 (22·99 to 41·07)	25·41 (20·39 to 31·24)	795·31 (645·73 to 970·92)	904·65 (831·22 to 979·07)	3·19 (2·35 to 4·27)	2·01 (1·63 to 2·46)	68·86 (55·50 to 83·87)
2017	8550·90 (7871·50 to 9228·32)	30·59 (22·51 to 40·34)	24·81 (19·80 to 30·52)	772·02 (620·44 to 943·68)	883·29 (809·83 to 958·18)	3·13 (2·27 to 4·21)	1·95 (1·57 to 2·38)	66·42 (53·44 to 81·10)
2018	8428·61 (7740·81 to 9094·57)	30·28 (22·19 to 40·15)	24·79 (20·09 to 30·57)	770·13 (618·61 to 940·04)	862·93 (791·31 to 934·20)	3·09 (2·24 to 4·18)	1·93 (1·56 to 2·36)	66·01 (53·14 to 80·40)
2019	8238·72 (7564·76 to 8877·23)	30·11 (21·97 to 40·01)	24·92 (19·86 to 31·03)	774·05 (618·29 to 953·80)	833·78 (764·31 to 902·16)	3·07 (2·22 to 4·14)	1·93 (1·55 to 2·38)	66·21 (52·77 to 81·40)
% change, 2010 to 2019	−13·37% (−14·90 to −11·71)	−21·49% (−26·48 to −16·53)	−26·51% (−31·82 to −20·84)	−30·70% (−35·75 to −25·03)	−20·60% (−22·09 to −19·10)	−23·59% (−28·11 to −19·22)	−33·19% (−37·82 to −28·13)	−34·82% (−39·61 to −29·61)
**HBV-related liver cancer**								
2010	13·40 (10·63 to 16·79)	9·38 (7·41 to 11·82)	8·38 (6·60 to 10·60)	224·79 (179·64 to 280·23)	1·13 (0·91 to 1·40)	0·76 (0·60 to 0·95)	0·66 (0·52 to 0·83)	19·05 (15·34 to 23·40)
2011	13·68 (10·87 to 17·12)	9·53 (7·53 to 12·04)	8·45 (6·62 to 10·68)	225·22 (180·68 to 280·05)	1·14 (0·92 to 1·41)	0·76 (0·61 to 0·95)	0·66 (0·52 to 0·83)	18·88 (15·26 to 23·12)
2012	13·95 (11·10 to 17·43)	9·67 (7·65 to 12·20)	8·55 (6·70 to 10·82)	226·75 (180·96 to 281·77)	1·15 (0·93 to 1·42)	0·76 (0·61 to 0·95)	0·66 (0·52 to 0·82)	18·80 (15·15 to 22·99)
2013	14·20 (11·30 to 17·74)	9·79 (7·73 to 12·36)	8·62 (6·74 to 10·90)	227·63 (181·11 to 283·30)	1·16 (0·93 to 1·43)	0·76 (0·61 to 0·95)	0·65 (0·52 to 0·82)	18·69 (15·09 to 22·89)
2014	14·43 (11·47 to 18·03)	9·90 (7·80 to 12·51)	8·70 (6·83 to 11·04)	229·22 (182·75 to 284·76)	1·17 (0·94 to 1·44)	0·76 (0·61 to 0·95)	0·65 (0·52 to 0·82)	18·63 (15·06 to 22·85)
2015	14·62 (11·60 to 18·28)	10·00 (7·86 to 12·64)	8·84 (6·91 to 11·22)	232·11 (184·27 to 288·55)	1·17 (0·94 to 1·44)	0·76 (0·61 to 0·95)	0·65 (0·52 to 0·82)	18·69 (15·10 to 22·83)
2016	14·65 (11·49 to 18·37)	9·99 (7·87 to 12·76)	8·83 (6·89 to 11·23)	230·77 (183·65 to 286·92)	1·16 (0·93 to 1·43)	0·75 (0·60 to 0·95)	0·64 (0·51 to 0·81)	18·40 (14·83 to 22·52)
2017	14·65 (11·48 to 18·54)	9·97 (7·74 to 12·73)	8·82 (6·85 to 11·32)	229·28 (182·51 to 287·29)	1·15 (0·93 to 1·43)	0·74 (0·59 to 0·93)	0·63 (0·50 to 0·80)	18·11 (14·60 to 22·22)
2018	14·78 (11·52 to 18·89)	10·05 (7·77 to 12·94)	8·88 (6·83 to 11·43)	230·20 (181·28 to 289·36)	1·15 (0·92 to 1·44)	0·74 (0·58 to 0·94)	0·63 (0·49 to 0·80)	18·05 (14·56 to 22·36)
2019	14·97 (11·51 to 19·47)	10·17 (7·79 to 13·40)	9·00 (6·88 to 11·62)	232·40 (182·90 to 296·29)	1·16 (0·91 to 1·48)	0·74 (0·58 to 0·96)	0·63 (0·49 to 0·81)	18·09 (14·49 to 22·56)
% change, 2010 to 2019	11·78% (−0·78 to 26·48)	8·42% (−2·79 to 21·65)	7·30% (0·19 to 16·00)	3·39% (−4·36 to 12·75)	2·71% (−8·84 to 15·77)	−2·10% (−12·24 to 9·82)	−4·14% (−10·70 to 4·20)	−5·07% (−12·02 to 4·08)

DALYs=disability-adjusted life-years. HBV=hepatitis B virus. UI=uncertainty interval.

The total DALY burden associated with HBV infection in 2019 was 19·95 thousand (95% UI 17·01 to 23·06) DALYs due to acute hepatitis B, 774·05 thousand (618·29 to 953·80) DALYs due to cirrhosis, and 232·40 thousand (182·90 to 296·29) DALYs due to liver cancer (table 1). Substantial reductions in DALYs since 2010 were estimated for acute hepatitis B (–27·82% [95% UI –36·92 to –20·24]) and HBV-related cirrhosis (–30·70% [–35·75 to –25·03]), but not for DALYs due to HBV-related liver cancer. Changes in age-standardised DALY rates closely resembled those observed for overall DALY counts (table 1).

### Burden of HCV in Europe

In 2019, there were an estimated 487·93 thousand (95% UI 423·13–566·83) incident cases of acute hepatitis C. The age-standardised incidence rate of acute hepatitis C decreased by 3·24% (1·17–5·02) and the age-standardised mortality rate decreased by 35·73% (23·48–47·75; table 2). HCV-related cirrhosis accounted for 11·87 million (95% UI 9·77–14·41) prevalent cases, 52·92 thousand (39·38–70·44) incident cases, and 36·89 thousand (29·94–45·56) deaths in 2019, with no significant change in these measures from 2010 to 2019. However, the age-standardised prevalence rate decreased by 6·37% (4·32–8·11), the age-standardised incidence decreased by 5·87% (1·01–11·24), and age-standardised mortality rate decreased by 11·11% (5·53–16·54; table 2). In 2019, there were an estimated 32·12 thousand (95% UI 25·74–39·96) prevalent cases and 24·13 thousand (19·47–29·58) incident cases of HCV-related liver cancer, and 23·07 thousand (18·95–27·31) deaths due to HCV-related liver cancer, with significant increases in all counts from 2010 to 2019. However, when considering age-standardised rates, the burden of HCV-related liver cancer remained stable from 2010 to 2019 (table 2).

**Table 2:** Prevalence, incidence, mortality, and DALYs attributable to HCV infection, by calendar year, 2010–19

	**Number, thousands (95% UI)**				**Age-standardised rate per 100 000 population (95% UI)**			
	Prevalent cases	Incident cases	Deaths	DALYs	Prevalence rate	Incidence rate	Mortality rate	DALY rate
**Acute hepatitis C**								
2010	55·20 (48·41 to 63·65)	478·39 (419·52 to 551·66)	0·09 (0·07 to 0·14)	3·91 (2·94 to 5·75)	6·70 (5·99 to 7·66)	58·11 (51·89 to 66·41)	0·01 (0·01 to 0·01)	0·46 (0·36 to 0·70)
2011	55·34 (48·52 to 63·90)	479·64 (420·55 to 553·78)	0·09 (0·06 to 0·14)	3·68 (2·79 to 5·40)	6·69 (5·97 to 7·66)	57·98 (51·70 to 66·43)	0·01 (0·01 to 0·01)	0·43 (0·34 to 0·64)
2012	55·23 (48·37 to 63·76)	478·65 (419·18 to 552·59)	0·08 (0·06 to 0·13)	3·47 (2·64 to 5·08)	6·64 (5·92 to 7·63)	57·58 (51·30 to 66·14)	0·01 (0·01 to 0·01)	0·41 (0·32 to 0·60)
2013	54·97 (48·17 to 63·54)	476·43 (417·48 to 550·66)	0·07 (0·06 to 0·12)	3·21 (2·45 to 4·64)	6·58 (5·86 to 7·58)	57·03 (50·77 to 65·67)	0·01 (0·01 to 0·01)	0·38 (0·29 to 0·55)
2014	54·73 (47·96 to 63·22)	474·36 (415·63 to 547·91)	0·07 (0·05 to 0·11)	3·13 (2·41 to 4·52)	6·52 (5·79 to 7·51)	56·49 (50·17 to 65·13)	0·01 (0·00 to 0·01)	0·36 (0·28 to 0·54)
2015	54·64 (47·78 to 63·08)	473·54 (414·14 to 546·69)	0·07 (0·05 to 0·11)	3·03 (2·32 to 4·47)	6·47 (5·74 to 7·48)	56·10 (49·74 to 64·78)	0·01 (0·00 to 0·01)	0·35 (0·27 to 0·53)
2016	54·47 (47·50 to 63·11)	472·10 (411·69 to 546·94)	0·06 (0·05 to 0·10)	2·90 (2·20 to 4·31)	6·41 (5·69 to 7·41)	55·58 (49·32 to 64·24)	0·01 (0·00 to 0·01)	0·34 (0·25 to 0·50)
2017	54·54 (47·33 to 63·58)	472·64 (410·18 to 551·06)	0·07 (0·05 to 0·10)	2·90 (2·20 to 4·30)	6·38 (5·62 to 7·36)	55·25 (48·72 to 63·78)	0·01 (0·00 to 0·01)	0·33 (0·25 to 0·49)
2018	55·25 (47·93 to 64·37)	478·84 (415·43 to 557·83)	0·07 (0·05 to 0·10)	2·87 (2·13 to 4·14)	6·42 (5·67 to 7·43)	55·60 (49·13 to 64·36)	0·01 (0·00 to 0·01)	0·32 (0·24 to 0·48)
2019	56·30 (48·82 to 65·40)	487·93 (423·13 to 566·83)	0·07 (0·05 to 0·10)	2·85 (2·10 to 4·08)	6·49 (5·73 to 7·52)	56·23 (49·64 to 65·14)	0·01 (0·00 to 0·01)	0·32 (0·23 to 0·47)
% change, 2010 to 2019	1·99% (−0·60 to 4·90)	1·99% (−0·60 to 4·90)	−28·06% (−40·99 to −14·33)	−27·07% (−39·34 to −15·97)	−3·24% (−5·02 to −1·17)	−3·24% (−5·02 to −1·17)	−35·73% (−47·75 to −23·48)	−31·37% (−43·52 to −19·90)
**HCV-related cirrhosis and other chronic liver diseases**								
2010	12 034·47 (9972·70 to 14 482·85)	55·62 (41·23 to 73·11)	37·63 (30·99 to 46·55)	1162·92 (959·64 to 1432·55)	1214·73 (997·97 to 1471·25)	5·76 (4·23 to 7·59)	3·14 (2·60 to 3·87)	104·40 (86·48 to 127·36)
2011	12 029·12 (9976·74 to 14 473·43)	55·57 (41·28 to 73·00)	36·63 (30·15 to 45·30)	1120·38 (921·58 to 1380·53)	1207·22 (991·81 to 1461·04)	5·76 (4·22 to 7·57)	3·01 (2·49 to 3·70)	99·59 (82·69 to 121·53)
2012	11 968·21 (9929·86 to 14 434·97)	55·49 (41·13 to 72·92)	36·58 (30·14 to 45·23)	1112·75 (914·98 to 1369·44)	1194·17 (982·30 to 1444·24)	5·74 (4·22 to 7·55)	2·97 (2·46 to 3·65)	98·15 (81·30 to 119·54)
2013	11 880·66 (9856·99 to 14 332·99)	55·37 (40·95 to 72·95)	36·62 (30·09 to 45·03)	1113·53 (917·37 to 1365·99)	1178·65 (969·05 to 1426·62)	5·72 (4·20 to 7·53)	2·95 (2·43 to 3·60)	97·70 (81·04 to 118·64)
2014	11 798·87 (9786·23 to 14 189·47)	55·22 (40·74 to 72·72)	36·97 (30·41 to 45·62)	1123·81 (925·63 to 1379·66)	1163·63 (955·82 to 1413·24)	5·70 (4·19 to 7·51)	2·95 (2·45 to 3·60)	98·08 (81·26 to 119·16)
2015	11 750·89 (9738·72 to 14 171·59)	55·01 (40·59 to 72·48)	38·31 (31·53 to 47·13)	1166·19 (961·79 to 1423·65)	1152·26 (946·39 to 1400·57)	5·67 (4·17 to 7·49)	3·03 (2·51 to 3·70)	101·40 (83·93 to 122·66)
2016	11 672·15 (9613·13 to 14 126·13)	54·59 (40·42 to 72·04)	37·61 (30·86 to 46·28)	1135·20 (934·96 to 1392·64)	1137·91 (930·87 to 1388·13)	5·62 (4·14 to 7·44)	2·94 (2·44 to 3·57)	97·81 (80·88 to 118·44)
2017	11 635·45 (9565·38 to 14 122·10)	54·19 (40·37 to 71·81)	36·92 (30·40 to 45·46)	1103·83 (912·55 to 1349·21)	1127·69 (914·96 to 1381·20)	5·56 (4·11 to 7·40)	2·85 (2·37 to 3·47)	94·32 (77·95 to 114·03)
2018	11 731·51 (9650·88 to 14 244·01)	53·70 (39·90 to 71·39)	36·84 (30·17 to 45·21)	1095·41 (901·37 to 1345·44)	1130·63 (919·11 to 1386·41)	5·51 (4·06 to 7·33)	2·81 (2·32 to 3·43)	93·09 (77·13 to 113·12)
2019	11 868·45 (9773·59 to 14 405·02)	52·92 (39·38 to 70·44)	36·89 (29·94 to 45·56)	1090·90 (883·24 to 1345·08)	1137·31 (925·43 to 1397·09)	5·43 (4·00 to 7·22)	2·79 (2·28 to 3·43)	92·28 (74·81 to 112·65)
% change, 2010 to 2019	−1·38% (−3·19 to 0·70)	−4·85% (−10·11 to 0·14)	−1·96% (−7·80 to 4·13)	−6·19% (−12·57 to −0·19)	−6·37% (−8·11 to −4·32)	−5·87% (−11·24 to −1·01)	−11·11% (−16·54 to −5·53)	−11·61% (−17·77 to −5·56)
**HCV-related liver cancer**								
2010	27·59 (22·86 to 32·44)	21·09 (17·47 to 24·75)	20·19 (16·76 to 23·68)	377·35 (309·09 to 447·49)	2·05 (1·70 to 2·42)	1·52 (1·26 to 1·79)	1·43 (1·19 to 1·68)	28·22 (22·98 to 33·69)
2011	28·39 (23·45 to 33·36)	21·62 (17·94 to 25·33)	20·71 (17·23 to 24·34)	385·25 (316·07 to 457·79)	2·08 (1·72 to 2·46)	1·53 (1·27 to 1·81)	1·44 (1·20 to 1·69)	28·42 (23·16 to 33·89)
2012	29·23 (24·15 to 34·35)	22·17 (18·38 to 26·00)	21·29 (17·73 to 25·05)	394·32 (323·42 to 468·77)	2·11 (1·74 to 2·49)	1·55 (1·28 to 1·82)	1·46 (1·21 to 1·71)	28·65 (23·33 to 34·09)
2013	30·03 (24·81 to 35·30)	22·69 (18·85 to 26·60)	21·66 (18·06 to 25·50)	399·84 (327·97 to 474·04)	2·13 (1·76 to 2·52)	1·56 (1·30 to 1·84)	1·46 (1·21 to 1·72)	28·64 (23·21 to 34·13)
2014	30·77 (25·42 to 36·21)	23·18 (19·28 to 27·25)	22·00 (18·29 to 25·95)	404·84 (331·07 to 480·94)	2·15 (1·78 to 2·56)	1·57 (1·30 to 1·85)	1·45 (1·21 to 1·72)	28·59 (23·19 to 34·18)
2015	31·36 (25·90 to 37·04)	23·58 (19·57 to 27·85)	22·40 (18·60 to 26·38)	411·07 (336·82 to 487·98)	2·17 (1·79 to 2·58)	1·57 (1·30 to 1·86)	1·46 (1·21 to 1·72)	28·63 (23·27 to 34·25)
2016	31·56 (25·80 to 37·37)	23·70 (19·62 to 28·07)	22·41 (18·59 to 26·45)	409·48 (335·32 to 489·75)	2·15 (1·76 to 2·56)	1·55 (1·28 to 1·84)	1·43 (1·19 to 1·70)	28·13 (22·89 to 33·73)
2017	31·65 (25·74 to 38·14)	23·74 (19·44 to 28·43)	22·66 (18·79 to 26·65)	412·75 (337·25 to 494·19)	2·13 (1·72 to 2·56)	1·53 (1·25 to 1·84)	1·43 (1·18 to 1·68)	27·94 (22·82 to 33·66)
2018	31·89 (25·84 to 38·67)	23·92 (19·43 to 28·70)	22·83 (18·87 to 26·95)	414·06 (338·42 to 496·30)	2·11 (1·71 to 2·56)	1·52 (1·23 to 1·83)	1·41 (1·17 to 1·68)	27·63 (22·34 to 33·32)
2019	32·12 (25·74 to 39·96)	24·13 (19·47 to 29·58)	23·07 (18·95 to 27·31)	416·47 (338·30 to 500·48)	2·10 (1·67 to 2·60)	1·51 (1·21 to 1·86)	1·40 (1·15 to 1·67)	27·42 (22·02 to 33·23)
% change, 2010 to 2019	16·41% (2·81 to 30·91)	14·44% (2·03 to 28·06)	14·24% (8·89 to 20·09)	10·37% (4·81 to 16·63)	2·55% (−9·50 to 15·73)	−0·61% (−11·56 to 11·44)	−1·88% (−6·50 to 3·04)	−2·84% (−7·75 to 2·63)

DALYs=disability-adjusted life-years. HCV=hepatitis C virus. UI=uncertainty interval.

The total DALY burden associated with HCV infection in Europe in 2019 was 2·85 thousand (95% UI 2·10–4·08) DALYs due to acute hepatitis, 1·09 million (0·88–1·35) DALYs due to cirrhosis, and 416·47 thousand (338·30–500·48) DALYs due to liver cancer (table 2). Between 2010 and 2019, DALYs due to acute hepatitis C decreased by 27·07% (95% UI 15·97–39·34) and those due to HCV-related cirrhosis decreased by 6·19% (0·19–12·57), while those due to liver cancer increased by 10·37% (4·81–16·63). Age-standardised DALY rates due to acute HCV and HCV-related cirrhosis showed similar reductions to the DALY counts between 2010 and 2019, but no significant change was observed for HCV-related liver cancer (table 2).

### Regional differences and temporal trends in the burden of HBV

**Figure 1:**
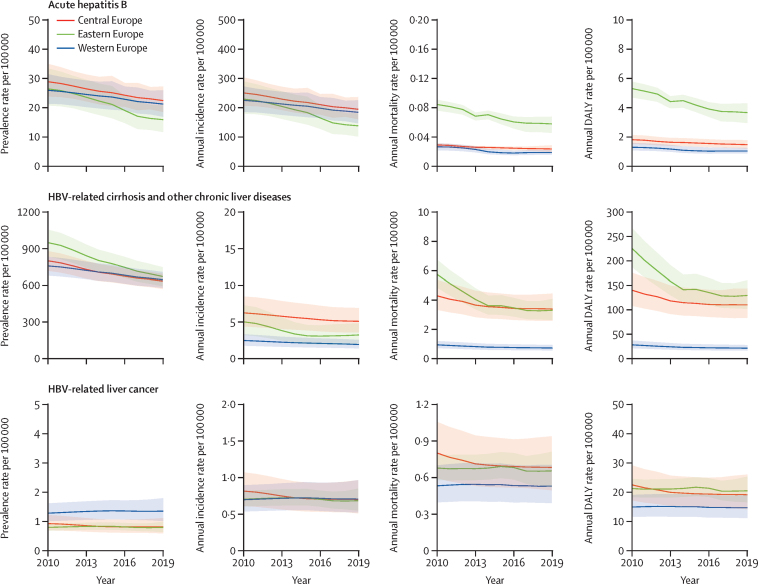
Temporal trends in age-standardised HBV disease burden measures in eastern, central, and western Europe, 2010–19 Graphs show the linear interpolation of point estimates (lines) and 95% uncertainly intervals (shaded regions) of the annual estimations. DALY=disability-adjusted life-year. HBV=hepatitis B virus.

In 2010, the age-standardised incidence rates of acute hepatitis B were similar in the three European regions (eastern, central, and western; appendix p 9). From 2010 to 2019, similar decreases in incidence rates were estimated for central Europe (–22·42% [95% UI –35·82 to –5·40]) and western Europe (–18·24% [–31·41 to –3·38]), whereas eastern Europe showed a larger decrease (–40·01% [–52·24 to –24·82]; appendix p 9). These decreases were consistent with trends in acute hepatitis B incidence and prevalence rates (figure 1). Deaths due to acute hepatitis B were highest in eastern Europe, and showed similar changes in eastern Europe (–31·37% [95% UI –47·15 to –18·94]) and western Europe (–29·22% [–34·10 to –23·21]) from 2010 to 2019 with a smaller reduction in central Europe (–18·24% [–31·41 to –3·38]; appendix p 9). The mortality rate declined more substantially between 2010 and 2013 for eastern Europe, while for western Europe the decline was more pronounced from 2010 to 2014 (figure 1). Central Europe showed a slight reduction from 2010 to 2019.

The prevalence rate for HBV-related cirrhosis decreased by 29·29% (95% UI 26·74–31·69) in eastern Europe, with a 2019 age-standardised prevalence rate of 833·78 (95% UI 764·31 to 902·16) cases per 100 000. Similarly, decreases in age-standardised prevalence rate were observed in western Europe (–20·75% [–22·56 to –18·72]) and central Europe (–14·87% [–16·29 to –13·03]; appendix p 9), and these decreases were constant over time for all three areas (figure 1). The mortality rate due to HBV-related cirrhosis decreased in all areas, with eastern Europe showing the highest reduction, concentrated in the 2010–14 period (figure 1; appendix p 9).

The burden of HBV-related liver cancer remained stable within the three areas, with western Europe having the highest prevalence rate (1·36 [95% UI 1·02–1·80] cases per 100 000) in 2019, followed by central Europe (0·82 [0·60–1·12]) and eastern Europe (0·80 [0·65–1·00]). The incidence and mortality rates of HBV-related liver cancer were similar in all three areas (figure 1; appendix p 10).

**Figure 2:**
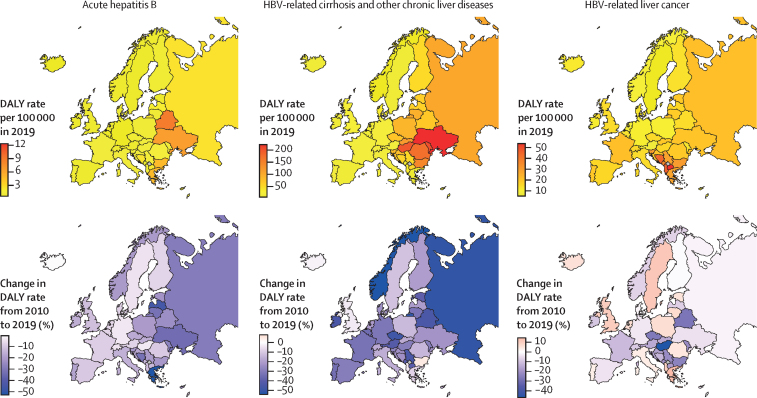
Age-standardised DALY rates for HBV-related diseases in Europe in 2019, and related 2010–19 percentage change DALY=disability-adjusted life-year. HBV=hepatitis B virus.

Geographical differences in the burden of disease are detailed in figure 2 and the appendix (pp 11–31).

### Regional differences and temporal trends in the burden of HCV

**Figure 3:**
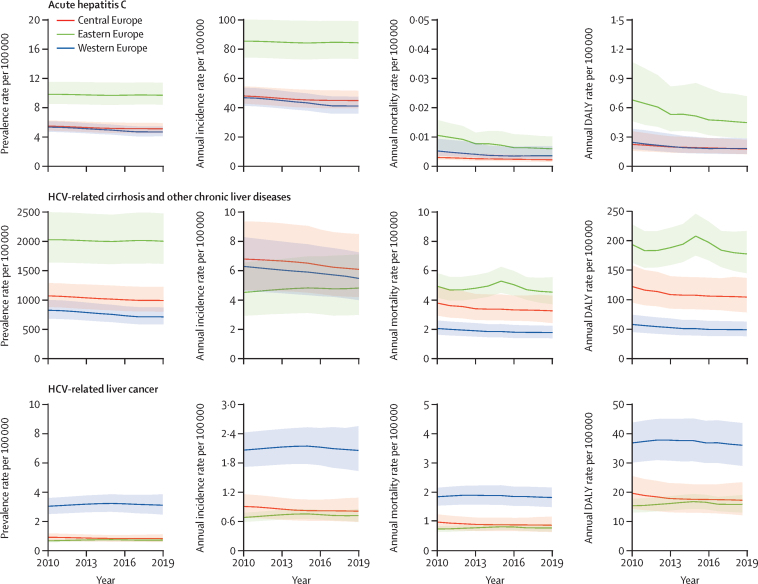
Temporal trends in age-standardised HCV disease burden measures in eastern, central, and western Europe, 2010–19 Graphs show the linear interpolation of point estimates (lines) and 95% uncertainly intervals (shaded regions) of the annual estimations. DALY=disability-adjusted life-year. HCV=hepatitis C virus.

In 2010, the age-standardised incidence rate of acute hepatitis C was highest in eastern Europe (85·27 [95% UI 74·08–99·94] cases per 100 000), and decreased slightly from 2010 to 2019 (appendix p 33). In 2010, the age-standardised incidence of acute hepatitis C was similar in central Europe (47·77 [42·60–54·30] cases per 100 000) and western Europe (46·59 [41·10–53·41] cases per 100 000), and these rates decreased by 6·51% (95% UI 3·90–8·84) and 12·20% (10·27–14·08), respectively, by 2019 (appendix p 33). By contrast, the prevalence rate remained constant over time in all three areas (figure 3). The mortality rate due to acute hepatitis C decreased by 44·17% (23·63–65·29) in eastern Europe, 32·28% (19·77–41·79) in western Europe, and 28·02% (9·25–44·21) in central Europe (figure 3; appendix p 33).

The age-standardised prevalence rate of HCV-related cirrhosis in 2010 was highest in eastern Europe, with 2026·94 (95% UI 1635·00 to 2497·29) cases per 100 000 (appendix p 33). The largest reduction was observed in western Europe (–13·75% [–15·48 to –12·13]) followed by central Europe (–7·32 [–10·54 to –3·73]), and no significant change was observed in eastern Europe (figure 3; appendix p 33). Similar trends were observed for age-standardised HCV-related cirrhosis mortality rates: eastern Europe had the highest rate and showed no significant reduction, whereas the largest reductions were seen in western and central Europe (figure 3; appendix p 33). The age-standardised incidence rate of HCV-related cirrhosis was slightly but not significantly lower in eastern Europe in 2010 (4·51 [95% UI 2·91 to 6·52] cases per 100 000) and in 2019 (4·80 [2·98 to 7·08] cases per 100 000) compared with western Europe (6·26 [4·63 to 8·26] cases per 100 000) in 2010 and 5·45 [3·98 to 7·22] cases per 100 000 in 2019) and central Europe (6·77 [4·67 to 9·34] cases per 100 000) in 2010 and 6·06 [4·20 to 8·47] cases per 100 000 in 2019). The age-standardised incidence rates of HCV-related cirrhosis decreased in all areas except for eastern Europe (appendix p 33).

Age-standardised prevalence, incidence, and mortality rates of HCV-related liver cancer remained stable from 2010 to 2019, with all measures highest in western Europe (figure 3; appendix p 34).

**Figure 4:**
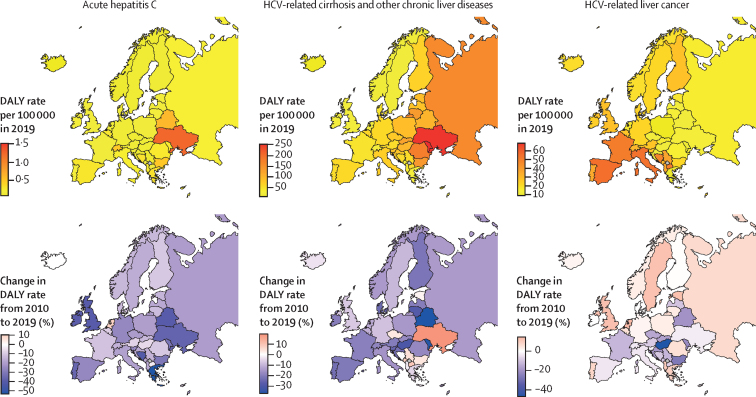
Age-standardised DALY rates for HCV-related diseases in Europe in 2019, and related 2010–19 percentage change DALY=disability-adjusted life-year. HCV=hepatitis C virus.

The geographical distribution of the burden of HCV is shown in figure 4 and the appendix (pp 35–55).

## Discussion

In our study, the burden of HBV and HCV remained high in Europe in 2019. In 2019, central and eastern Europe had higher age-standardised mortality rates for HBV-related cirrhosis and liver cancer than western Europe. The age-standardised prevalence and mortality rates for HCV-related cirrhosis were highest in eastern Europe, while the prevalence and mortality rates of HCV-related liver cancer were highest in western Europe. Monitoring progress in terms of the incidence of acute hepatitis and changes in burden associated with end-stage liver diseases (cirrhosis and liver cancer) is an essential component of the HCV and HBV elimination effort.^25^ These data will inform policy makers about the progress of European countries towards reaching the goal of eliminating viral hepatitis as a public health threat by 2030 and provide essential information to identify regions where implementation of effective strategies is needed most.

Between 2010 and 2019, the burden of cirrhosis due to HBV and HCV decreased. HBV-related cirrhosis and acute hepatitis B showed the most substantial reductions among all HBV-related and HCV-related diseases assessed. These data follow improvements in HBV prevention policies in Europe, where 83·3% (20 of 24) EU and European Economic Area (EEA) countries that have implemented universal childhood vaccination programmes have achieved 90% coverage. Additionally, 27 European countries started HBV screening for pregnant women, with promising results in the few countries that provide coverage data.^26^, ^27^ By contrast, the burden of HBV-related liver cancer did not show any significant improvements between 2010 and 2019. Neonatal HBV vaccination is likely to result in reductions in HBV-related liver cancer in the future, but the effect was not seen in this study as hepatocellular carcinoma is more common in older adults, with a median age at diagnosis of around 60 years, whereas the first neonatal vaccination programmes began in around 1990.

No changes were observed in age-standardised rates of liver cancer incidence, prevalence, mortality, and DALYs from 2010 to 2019, whereas we observed increases in all-age incidence, prevalence, mortality, and DALY counts. The increasing number of cases of HCV-related liver cancer might be driven by changes in population demographics, with ageing of people chronically infected with HCV, as well as the fact that effective treatment and intervention only became available since 2015 and problems with treatment access have been reported in many countries.^28^, ^29^, ^30^ Our study did not show significant reductions in the burden of acute hepatitis C, despite the development of direct-acting antiviral therapy, which became available in the European market in 2014–15. However, direct-acting antivirals are expected to contribute to decreases in deaths due to cirrhosis and liver cancer in the coming years, particularly in countries with a higher proportion of people being treated.

HBV and HCV are still associated with high health burdens, highlighting the need for more intensive and coordinated intervention in European countries to reach the goal of elimination by 2030. In 2018, only seven European countries were considered to be on track for this target, including several high-income countries and one low-to-middle-income country.^29^ In 2019, around a third of all EU and EEA countries reported no action plan or strategy for hepatitis prevention and control and, of those with a plan or strategy, nearly half reported no funding for implementation.^26^ The higher burden of HBV and HCV in countries without prevention and control plans or strategies, or without funding for implementations, highlights the importance of strategies supported by economic resources to achieve the target of elimination by 2030 throughout European countries.^31^

So far, many frameworks have been proposed to reach the goal of elimination of viral hepatitis by 2030.^2,6,26,27,29–33^ However, a major barrier to elimination is insufficient funding. Prevention, screening, and treatment programmes for hepatitis B and C are greatly underfunded compared with those for HIV, tuberculosis, and malaria. Hepatitis elimination programmes are not substantially financed by the Bill & Melinda Gates Foundation, The Global Fund to Fight AIDS, Tuberculosis, and Malaria, or other international funders.^32^, ^33^ Only programmes for people with HIV co-infection are covered and, without support from these or similar organisations, the 2030 elimination targets are unlikely to be achieved in the whole European area. However, it is important that each country works to define, implement, and fund their own sustainable elimination programmes. Egypt provides a good example of an HCV elimination programme applied in 2014.^17^, ^34^ Between 2014 and 2017, the Egyptian National Committee for Control of Viral Hepatitis provided no-cost HCV treatment to more than 2 million people. After provision of treatment for those already diagnosed, in 2018, Egypt introduced a national HCV screening initiative with a target population of 62·5 million and reached screening coverage of 79·4%.^34^ The success of the Egyptian programme was characterised by a strong political will, social pressure from affected communities, cheap testing, treatment free of charge to patients, and efficient programme management, which also included shifting of some tasks to primary care physicians.^29^, ^34^ Other good examples have been provided by Georgia for HCV and China for HBV.^6^

Another major barrier to hepatitis elimination is, in part, that HCV in high-income countries largely affects people who inject drugs and people who are incarcerated, who are generally highly marginalised, which has a substantial impact on the social response, with less consideration by society of the need for targeted campaigns, screening, and treatment programmes to eliminate the virus in these subpopulations. In low and middle-income countries, stigmatised lifestyles are less associated with HCV and HBV transmission; in these countries, unsafe health-care practices are one of the main drivers of infection, although the affected populations still have a considerable vulnerability.^2^, ^6^, ^29^, ^30^, ^31^, ^32^, ^33^

The 2020–21 global health challenges related to SARS-CoV-2 infection have increased uncertainty around the definition and implementation of existing action plans to eliminate viral hepatitis due to the competing priorities and opportunity costs of investment in other infectious and chronic disease management programmes. The COVID-19 pandemic has made access to diagnosis of HBV and HCV infection and linkage to care very difficult, constituting a potential barrier that could prevent achievement of HCV and HBV elimination.^35^, ^36^ Despite these devastating impacts, new opportunities to achieve the hepatitis elimination goal can be found in the work done in the COVID-19 pandemic response, such as significant investments in surveillance and reporting systems. Many elements of the health-system response, such as rapid training and mobilisation of skilled health workers, decentralisation of services, novel community-based remote models of care such as telemedicine, resource and task sharing, and integration of multiple health systems to deliver public health responses could be leveraged for hepatitis B and C elimination.^32^, ^33^, ^37^

We acknowledge several limitations to our study. The main limitation of GBD estimates is the low quality and availability of data in some countries; however, robust statistical methods were used to reduce these limitations in countries with low data availability. In these locations, a combination of predictive covariates and trends in neighbouring countries was applied. The variation in the availability of high-quality data across locations influences the uncertainty associated with all estimates and is reflected in our results by wide 95% UIs for some estimates. Moreover, although GBD statistical modelling is designed to capture uncertainty related to multiple sources of bias (such as selection, measurement, and model specification), there is still some uncertainty not included in the UIs. Defining the proportions of acute hepatitis, cirrhosis, and liver cancer due to HBV and HCV is difficult because the aetiological models are informed by small amounts of data. To overcome this issue, models rely on covariates and space–time extrapolation. This methodological issue could be improved with the support of more longitudinal studies and better ascertainment of HBV-related and HCV-related diseases. Furthermore, seroprevalence data can frequently overestimate the number of people with active chronic infection, leading to misclassification of chronic hepatitis due to the absence of specific markers for acute hepatitis. Estimates of chronic hepatitis C do not account for virus-clearing treatments in many countries, and future GBD estimation must include more data related to this aspect. However, in GBD 2019, we changed the strategy of modelling deaths for acute hepatitis B and C from a natural history model relying on inpatient case fatality rates, to CODEm models after predicting type-specific acute hepatitis deaths from vital registration data with specified hepatitis type.^17^ Additionally, DisMod-MR was used to determine the proportions of deaths by underlying causes of cirrhosis and liver cancer.^17^ Furthermore, the contribution of immigration to hepatitis mortality estimates is not captured by this study. This issue could make it difficult to evaluate the effect of interventions on domestic populations, particularly if immigration is from countries with a high prevalence of hepatitis.^38^ Finally, other specific conditions could be associated with issues related to poor outcomes, and, particularly, to death classification, in people infected with HBV and HCV. Although the methods used within GBD, as far as aetiology is concerned, were developed to ascribe the whole burden of a specific condition to the underlying cause, the clinical complexity of liver disease could make these estimates subject to certain limitations and uncertainties, considering the pathogenesis and clinical aspects of HBV-attributable and HCV-attributable disease.^39^

Although viral infection is a significant factor in the development of acute hepatitis, the severity and clinical outcomes of the disease are influenced by a range of other factors. Age, sex, lifestyle factors (eg, alcohol consumption or substance use), genetic predisposition, and the immune system's ability to control viral replication and limit inflammation can substantially affect the morbidity and mortality associated with viral hepatitis.^40^ The presence of co-infections (eg, HIV or hepatitis delta virus) or underlying comorbidities, such as liver disease, obesity, or diabetes, also worsens the clinical outcomes associated with HBV-related or HCV-related liver injury.^41^, ^42^

GBD considers the contributions of HBV and HCV to the overall burden of chronic liver disease, including chronic liver injury, cirrhosis, hepatocellular carcinoma, and deaths attributed to hepatitis-related complications. By analysing the available evidence, GBD estimates the probability of developing these conditions after being infected with HBV or HCV based on available attributable-fraction studies, accounting for comorbidities and adjusting the estimates accordingly.^17^, ^22^ However, as for acute hepatitis, the viral origin cannot fully provide a comprehensive explanation of clinical outcomes in patients with chronic liver disease. In addition to factors that can influence the clinical course of acute hepatitis, progression to chronic liver disease can vary between individuals due to factors that contribute to liver inflammation and fibrosis, including NAFLD and NASH, and environmental factors (eg, some medications or industrial chemicals).^43^, ^44^, ^45^ HCV and HBV are also associated with several extrahepatic manifestations, including mixed cryoglobulinaemia, diabetes, cardiocerebrovascular disease, lymphoma, and autoimmune diseases. These non-liver-related complications increase the complexity of liver disease and the roles of HCV or HBV infection in its burden, morbidity, and mortality.^46^

Considering the above, the attribution of liver disease and outcomes to HBV or HCV does not necessarily reflect the clinical cause of the outcome (ie, disability or death). Indeed, the GBD approach, as far as aetiology is concerned, is to ascribe the whole burden of a specific condition to the underlying cause, gathering patients with different clinical histories and comorbidities that could be related to death within the same group.

In conclusion, hepatitis B and C still constitute a substantial burden in Europe, and the ambitious goal of elimination by 2030 is far from being achieved. Some well structured frameworks for hepatitis prevention and control are available, and a few countries have demonstrated the potential to implement them in effective and sustainable ways.^6^, ^34^, ^47^ However, many countries still have no action plan or strategy for hepatitis prevention and control, or funding for implementation.^26^, ^27^ Elimination of viral hepatitis needs a greater commitment from governments, health-care systems, national and international institutions, civil society, and donors. The return on the investment required for prevention, detection, and treatment of viral hepatitis could be seen in future reductions in deaths due to HCV-related or HBV-related liver diseases, incidence of advanced liver disease complications, and related management costs. Enhanced monitoring and evaluation systems such as GBD are essential to hepatitis elimination responses in European countries and worldwide.

## Data sharing

To download the GBD 2019 data used in these analyses, please visit the Global Health Data Exchange website.

## Declaration of interests

J V Lazarus reports grants or contracts from AbbVie, Gilead Sciences, MSD, and Roche Diagnostics; royalties and licenses from Novavax; payment or honoraria for lectures from AbbVie, Gilead Sciences, Intercept, Janssen, and Novo Nordisk; participation on a data safety monitoring board or advisory board for the study “Same-visit hepatitis C testing and treatment to accelerate cure among people who inject drugs (The QuickStart Study): a cluster randomised control trial” (Australia); and leadership or fiduciary roles in board, society, committee, or advocacy groups, paid or unpaid, with the European Association for the Study of the Liver Public Health and Policy Committee as a member, HIV Outcomes as a co-chair, and SHARE Global Health Foundation; all outside the submitted work. P C Matthews reports support for the present manuscript from The Francis Crick Institute, London, UK; grants and contracts from The Wellcome Trust and University College London Hospital National Institute for Health and Care Research Biomedical Research Centre; and funding from GSK for a PhD student fellowship. A-F A Mentis reports grants and contracts from MilkSafe (A novel pipeline to enrich formula milk using omics technologies), which is co-financed by the European Regional Development Fund of the EU and Greek national funds through the Operational Program Competitiveness, Entrepreneurship and Innovation, under the call RESEARCH–CREATE–INNOVATE (project code T2EDK-02222), as well as from ELIDEK (Hellenic Foundation for Research and Innovation, MIMS-860); payment for expert testimony from serving as external peer-reviewer for Fondazione Cariplo, Italy; leadership or fiduciary roles in board, society, committee or advocacy groups, paid or unpaid, as an editorial board member for *Systemic Reviews* and *Annals of Epidemiology*, and as Associate Editor for *Translational Psychiatry*; other financial or non-financial support from the BGI group as a scientific officer; outside the submitted work. M J Postma reports stock or stock options in PAG BV (Groningen, Netherlands) and HealthEcore (Zeist, Netherlands). All other authors declare no competing interests.
