# Supplementary material for: Hepatitis B and C in Europe: an update from the Global Burden of Disease Study 2019
Source: Lancet Public Health. 2023 Aug 24;8(9):e701–16. doi: 10.1016/S2468-2667(23)00149-4 (PMC11138131; doi:10.1016/S2468-2667(23)00149-4)
Supplement: Supplementary appendix [file mmc1.pdf]

THE LANCET
Public Health

## Supplementary appendix

This appendix formed part of the original submission and has been peer reviewed. We post it as supplied by the authors.

Supplement to: GBD 2019 Europe Hepatitis B & C Collaborators. Hepatitis B and C in Europe: an update from the Global Burden of Disease Study 2019. *Lancet Public Health* 2023; **8:** e701–16.

# Supplementary Methods and Results Appendix to "Hepatitis B and C in Europe: an update from the Global Burden of Disease Study 2019"

**Table of content**


1. Statement of GATHER compliance
2. Methods - Diseases associated with HBV and HCV definition.
3. References
4. GATHER CECKLIST
5. Authors' Contributions

Supplementary Tables

Supplement Table A. "WHO Europe" countries stratifies by European Area: Eastern, Central, Western.

Supplement Table B. Prevalence, incidence, Mortality and DALYs attributable to HBV in 2010 and 2019, by European region.

Supplement Table C. Prevalence, incidence, mortality and DALYs attributable to HBV in 2010 and 2019, by Central Europe countries.

Supplement Table D. Prevalence, incidence, mortality and DALYs attributable to HBV in 2010 and 2019, by Eastern Europe countries.

Supplement Table E. Prevalence, incidence, mortality and DALYs attributable to HBV in 2010 and 2019, by Western Europe countries.

Supplement Table F. Prevalence, incidence, Mortality and DALYs attributable to HCV in 2010 and 2019, by European region.

Supplement Table G. Prevalence, incidence, mortality and DALYs attributable to HCV in 2010 and 2019, by Central Europe countries.

Supplement Table H. Prevalence, incidence, mortality and DALYs attributable to HCV in 2010 and 2019, by Eastern Europe countries.

Supplement Table I. Prevalence, incidence, mortality and DALYs attributable to HCV in 2010 and 2019, by Western Europe countries.

## 1. Statement of GATHER compliance

This study was developed in accordance with the Guidelines for Accurate and Transparent Health Estimates Reporting (GATHER) guidelines.[1] Websites cited in this appendix were last accessed May 18, 2022, unless otherwise stated.

## 2. METHODS

**Diseases associated with HBV and HCV definition.**

In the Global Burden of Disease (GBD) study, diseases and injuries are organized into a levelled cause hierarchy to accommodate different purposes and needs. The first two levels aggregate causes into general groupings. Level 1 defines three cause groups: communicable, maternal, neonatal, and nutritional diseases (Group 1); non-communicable diseases (Group 2); and injuries (Group 3). The disaggregation into levels 3 and 4 contains the finest level of detail for causes captured in GBD 2019 study. Sequelae of diseases and injuries are organised at Levels 5 and 6 of the hierarchy. In GBD, sequelae are defined as distinct, mutually exclusive categories of health consequences that can be directly attributed to a cause.

For the aim of this study, we extracted GBD study estimates related to level 4 diseases associated with hepatitis B virus (HBV) and hepatitis C virus (HCV) from 2010 to 2019 in Europe: acute hepatitis, cirrhosis and other chronic liver diseases (collectively referred to as cirrhosis in this paper) and liver cancer. Acute hepatitis in GBD methodology refers to "acute viral hepatitis caused by the hepatitis A, B, C or E viruses.[2] Cirrhosis is defined as a "chronic liver disease in which there is progressive destruction of functional hepatic cells and replacement with fibrosis (scarring) of the liver"[1] and the causes grouped in the "other chronic liver diseases" category mainly included autoimmune hepatitis, toxic liver diseases, other inflammatory liver diseases, chronic hepatitis not specified, and other diseases of the liver.[3] Finally, liver cancer GBD estimates are derived from registries and causes of death data and defined by International Classification of Disease vs. 10 (ICD10) codes C.22- C.22.8, D13.4. in terms of incidence, prevalence, mortality and Disability Adjusted Life-Years DALYs and the amount attributable to risk factors or causes.

## 3. References

1. Stevens G, Alkema L, Black R, et al. Guidelines for accurate and transparent health estimates reporting: the GATHER statement. Lancet 2016; 388: 19–23.

2. GBD 2019 Diseases and Injuries Collaborators. Global burden of 369 diseases and injuries in 204 countries and territories, 1990-2019: a systematic analysis for the Global Burden of Disease Study 2019. Lancet. 2020 Oct 17;396(10258):1204-1222. Erratum in: Lancet. 2020 Nov 14;396(10262):1562.
3. GBD 2017 Cirrhosis Collaborators. The global, regional, and national burden of cirrhosis by cause in 195 countries and territories, 1990–2017: a systematic analysis for the Global Burden of Disease Study 2017. Lancet Gastroenterol Hepatol 2020

## 4. GHATER Checklist

| Item # | Checklist item | Reported on page# |
|---|---|---|
| **Objectives and funding** | | |
| 1 | Define the indicator(s), populations (including age, sex, and geographic entities), and time period(s) for which estimates were made. | Main text (Introduction, pg. 5) |
| 2 | List the funding sources for the work. | Main text (Methods; pg. 9) |
| **Data Inputs** | | |
| For all data inputs from multiple sources that are synthesized as part of the study: | | |
| 3 | Describe how the data were identified and how the data were accessed. | Main text (Methods; pg. 5-9) Appendix (pg. 2) |
| 4 | Specify the inclusion and exclusion criteria. Identify all ad-hoc exclusions. | Main text (Methods; pg. 6-7) |
| 5 | Provide information on all included data sources and their main characteristics. For each data source used, report reference information or contact name/institution, population represented, data collection method, year(s) of data collection, sex and age range, diagnostic criteria or measurement method, and sample size, as relevant. | Online data citation tools http://ghdx.healthdata.org/gbd-2019 |
| 6 | Identify and describe any categories of input data that have potentially important biases (e.g., based on characteristics listed in item 5). | Main text (Methods; pg. 6) |
| For data inputs that contribute to the analysis but were not synthesized as part of the study: | | |
| 7 | Describe and give sources for any other data inputs. | Online data citation tools http://ghdx.healthdata.org/gbd-2019 |
| For all data inputs: | | |
| 8 | Provide all data inputs in a file format from which data can be efficiently extracted (e.g., a spreadsheet rather than a PDF), including all relevant meta-data listed in item 5. For any data inputs that cannot be shared because of ethical or legal reasons, such as third-party ownership, provide a contact name or the name of the institution that retains the right to the data. | Online data visualization tools, data query tools, and the Global Health Data Exchange http://ghdx.healthdata.org |
| **Data analysis** | | |
| 9 | Provide a conceptual overview of the data analysis method. A diagram may be helpful. | Main text (Methods; pg. 9) |
| 10 | Provide a detailed description of all steps of the analysis, including mathematical formulae. This description should cover, as relevant, data cleaning, data pre-processing, data adjustments and weighting of data sources, and mathematical or statistical model(s). | Main text (Methods; pg. 6-9) |
| 11 | Describe how candidate models were evaluated and how the final model(s) were selected. | Main text (Methods; pg. 6) |
| 1 | Provide the results of an evaluation of model performance, if done, as well as the | Main text |

| | | |
|---|---|---|
| 2 | results of any relevant sensitivity analysis. | (Methods; pg. 6) |
| 13 | Describe methods for calculating uncertainty of the estimates. State which sources of uncertainty were, and were not, accounted for in the uncertainty analysis. | Main text (Methods; pg. 6) |
| 14 | State how analytic or statistical source code used to generate estimates can be accessed. | Code is provided in an online repository, http://ghdx.healthdata.org/gbd-2019/code |
| **Results and Discussion** | | |
| 15 | Provide published estimates in a file format from which data can be efficiently extracted. | Visualization tools, data query tools, and the Global Health Data Exchange, http://ghdx.healthdata.org/gbd-2019 |
| 16 | Report a quantitative measure of the uncertainty of the estimates (e.g. uncertainty intervals). | Main text (Results, Table 1,2,3,4), Appendix (Supplemental Table B1,B2,C1,C2,D1 and D2, pg 8-49), and online data tools |
| 17 | Interpret results in light of existing evidence. If updating a previous set of estimates, describe the reasons for changes in estimates. | Main text (Discussion, pg 12-15) |
| 18 | Discuss limitations of the estimates. Include a discussion of any modelling assumptions or data limitations that affect interpretation of the estimates. | Main text (Discussion, pg 14-15) |

This checklist should be used in conjunction with the GATHER statement and Explanation and Elaboration document, found on gather-statement.org

## 5. **Authors' Contributions**

*Providing data or critical feedback on data sources*

Ayman Ahmed, Anton A Artamonov, Maciej Banach, Akshaya Srikanth Bhagavathula, Sara Conti, Paolo Angelo Cortesi, Mostafa Dianatinasab, M Ashworth Dirac, Adeniyi Francis Fagbamigbe, Davide Golinelli, Mihajlo Jakovljevic, Jacek Jerzy Jozwiak, Zubair Kabir, Rovshan Khalilov, Moien AB Khan, Khaled Khatab, Jeffrey V Lazarus, Caterina Ledda, Lorenzo Giovanni Mantovani, Babak Moazen, Shafiu Mohammed, Lorenzo Monasta, Francesk Mulita, Christopher J L Murray, Ionut Negoi, Bogdan Oancea, Jay Patel, Maarten J Postma, Salman Rawaf, Milena M Santric-Milicevic, Valentin Yurievich Skryabin, Anna Aleksandrovna Skryabina, Rafael Tabarés-Seisdedos, Mircea Tampa, Nuno Taveira, Marcos Roberto Tovani-Palone, Ronny Westerman, and Mikhail Sergeevich Zastrozhin.

*Developing methods or computational machinery*

Ippazio Cosimo Antonazzo, Mostafa Dianatinasab, Adeniyi Francis Fagbamigbe, Simon I Hay, Rovshan Khalilov, Francesk Mulita, Christopher J L Murray, Ronny Westerman, and Mikhail Sergeevich Zastrozhin.

*Providing critical feedback on methods or results*

Ayman Ahmed, Catalina Liliana Andrei, Tudorel Andrei, Ippazio Cosimo Antonazzo, Anton A Artamonov, Till Winfried Bärnighausen, Maciej Banach, Akshaya Srikanth Bhagavathula, Giulia Carreras, Sheng-Chia Chung, Sara Conti, Mostafa Dianatinasab, M Ashworth Dirac, Eleonora Dubljanin, Oyewole Christopher Durojaiye, Ifeanyi Jude Ezeonwumelu, Adeniyi Francis Fagbamigbe, Pietro Ferrara, Florian Fischer, Carla Fornari, Ekaterina Vladimirovna Glushkova, Shoaib Hassan, Simon I Hay, Mihaela Hostiuc, Irena M Ilic, Milena D Ilic, Mihajlo Jakovljevic, Elham Jamshidi, Jacek Jerzy Jozwiak, Zubair Kabir, Joonas H Kauppila, Rovshan Khalilov, Moien AB Khan, Khaled Khatab, Ai Koyanagi, Carlo La Vecchia, Caterina Ledda, Joana A Loureiro, Lorenzo Giovanni Mantovani, Philippa C Matthews, Giampiero Mazzaglia, Alexios-Fotios A Mentis, Tomislav Mestrovic, Babak Moazen, Shafiu Mohammed, Francesk Mulita, Christopher J L Murray, Ionut Negoi, Bogdan Oancea, Claudia Palladino, Jay Patel, Ionela-Roxana Petcu, Maarten J Postma, David Laith Rawaf, Salman Rawaf, Esperanza Romero-Rodríguez, Milena M Santric-Milicevic, Valentin Yurievich Skryabin, Anna Aleksandrovna Skryabina, Rafael Tabarés-Seisdedos, Mircea Tampa, Nuno Taveira, Arulmani Thiyagarajan, Marcos Roberto Tovani-Palone, Ronny Westerman, and Mikhail Sergeevich Zastrozhin.

*Drafting the work or revising is critically for important intellectual content*

Ayman Ahmed, Catalina Liliana Andrei, Tudorel Andrei, Ippazio Cosimo Antonazzo, Till Winfried Bärnighausen, Maciej Banach, Carl Michael Baravelli, Nikolay Ivanovich Briko, Daniela Calina, Sara Conti, Paolo Angelo Cortesi, Mostafa Dianatinasab, M Ashworth Dirac, Eleonora Dubljanin, Oyewole Christopher Durojaiye, Adeniyi Francis Fagbamigbe, Pietro Ferrara, Florian Fischer, Carla Fornari, Silvano Gallus, Davide Golinelli, Giuseppe Gorini, Shoaib Hassan, Irena M Ilic, Milena D Ilic, Mihajlo Jakovljevic, Jacek Jerzy Jozwiak, Joonas H Kauppila, Rovshan Khalilov, Moien AB Khan, Khaled Khatab, Ai Koyanagi, Carlo La Vecchia, Caterina Ledda, Miriam Levi, Platon D Lopukhov, Joana A Loureiro, Lorenzo Giovanni Mantovani, Philippa C Matthews, Giampiero Mazzaglia, Alexios-Fotios A Mentis, Tomislav Mestrovic, Babak Moazen, Shafiu Mohammed, Lorenzo Monasta, Christopher J L Murray, Ionut Negoi, Bogdan Oancea, Claudia Palladino, Jay Patel, Ionela-Roxana Petcu, Maarten J Postma, David Laith Rawaf, Salman Rawaf, Esperanza Romero-Rodríguez, Milena M Santric-Milicevic, Valentin Yurievich Skryabin, Anna Aleksandrovna Skryabina, Nuno Taveira, Arulmani Thiyagarajan, Marcos Roberto Tovani-Palone, Ronny Westerman, and Mikhail Sergeevich Zastrozhin.

*Management of the overall research enterprise*

Paolo Angelo Cortesi, Simon I Hay, Lorenzo Giovanni Mantovani, and Christopher J L Murray.

## Supplementary Tables

**Supplemental Table A.** “WHO Europe” countries stratifies by European Area: Eastern, Central, Western.

| European Area | N of Countries | Countries |
|---|---|---|
| Central | 13 | Albania; Bosnia and Herzegovina;Bulgaria; Croatia; Czechia; Hungary; Montenegro; North Macedonia; Poland; Romania; Serbia; Slovakia; Slovenia |
| Eastern | 7 | Belarus; Estonia; Latvia; Lithuania; Republic of Moldova; Russian Federation; Ukraine |
| Western | 24 | Andorra; Austria; Belgium; Cyprus; Denmark; Finland; France; Germany; Greece; Iceland; Ireland; Israel; Italy; Luxembourg; Malta; Monaco; Netherlands; Norway; Portugal; San Marino; Spain; Sweden; Switzerland; United Kingdom |

**Supplement Table B.** Prevalence, incidence, Mortality and DALYs attributable to HBV in 2010 and 2019, by European region.

| | | N in thousand (95%UI) | | | | Age- standardized rate x 100,000 (95%UI) | | | |
|---|---|---|---|---|---|---|---|---|---|
| | | **Acute hepatitis** | | | | | | | |
| | **Year** | **Prevalence** | **Incidence** | **Mortality** | **DALYs** | **Prevalence** | **Incidence** | **Mortality** | **DALYs** |
| **Eastern Europe** | **2010** | 57·85 | 501·34 | 0·19 | 10·33 | 26·42 | 228·94 | 0·08 | 5·29 |
| | | ( 44·77; 74·41) | ( 388·05; 644·84) | (0·17;0·20) | ( 9·30;11·27) | (20·69;33·40) | (179·28;289·49) | (0·08;0·09) | ( 4·69; 5·77) |
| | **2019** | 35·06 | 303·89 | 0·12 | 6·68 | 15·85 | 137·34 | 0·06 | 3·65 |
| | | ( 25·51; 45·80) | ( 221·09; 396·95) | (0·10;0·14) | ( 5·31; 7·81) | (11·60;20·71) | (100·50;179·48) | (0·05;0·07) | ( 2·93; 4·28) |
| | **Change (%) 2010-2019** | -39·39 | -39·39 | -33·97 | -35·34 | -40·01 | -40·01 | -31·37 | -31·07 |
| | | (-51·61;-24·84) | (-51·61;-24·84) | (-49·82;-21·67) | (-49·78;-24·46) | (-52·24;-24·82) | (-52·24;-24·82) | (-47·15;-18·94) | (-45·73;-19·70) |
| **Central Europe** | **2010** | 37·36 | 323·78 | 0·04 | 2·31 | 28·86 | 250·16 | 0·03 | 1·80 |
| | | ( 30·84; 45·30) | ( 267·27; 392·61) | (0·04;0·05) | ( 1·97; 2·73) | (23·81;34·98) | (206·33;303·14) | (0·03;0·03) | ( 1·51; 2·11) |
| | **2019** | 29·55 | 256·08 | 0·04 | 1·88 | 22·39 | 194·07 | 0·02 | 1·45 |
| | | ( 24·30; 36·02) | ( 210·62; 312·19) | (0·03;0·04) | ( 1·55; 2·28) | (18·46;27·22) | (159·99;235·88) | (0·02;0·03) | ( 1·18; 1·77) |
| | **Change (%) 2010-2019** | -20·91 | -20·91 | -14·79 | -18·36 | -22·42 | -22·42 | -18·78 | -19·00 |
| | | (-34·65; -3·69) | (-34·65; -3·69) | (-27·95; 0·01) | (-28·22; -6·25) | (-35·82; -5·40) | (-35·82; -5·40) | (-31·78; -3·63) | (-29·90; -5·80) |
| **Western Europe** | **2010** | 109·71 | 950·81 | 0·18 | 6·77 | 25·92 | 224·64 | 0·03 | 1·28 |
| | | ( 87·28;135·07) | ( 756·43;1170·61) | (0·15;0·21) | ( 5·71; 8·23) | (21·32;31·43) | (184·78;272·38) | (0·02;0·03) | ( 1·06; 1·58) |
| | **2019** | 96·00 | 832·03 | 0·14 | 5·57 | 21·19 | 183·68 | 0·02 | 1·02 |
| | | ( 73·71;118·57) | ( 638·82;1027·63) | (0·11;0·16) | ( 4·68; 6·79) | (16·84;25·93) | (145·91;224·75) | (0·02;0·02) | ( 0·85; 1·25) |
| | **Change (%) 2010-2019** | -12·49 | -12·49 | -24·82 | -17·69 | -18·24 | -18·24 | -29·22 | -20·46 |
| | | (-27·16; 4·21) | (-27·16; 4·21) | (-30·21;-18·11) | (-24·48; -9·49) | (-31·41; -3·38) | (-31·41; -3·38) | (-34·10;-23·21) | (-27·95;-12·03) |
| | | **Cirrhosis and other chronic liver diseases due to hepatitis** | | | | | | | |
| | **Year** | **Prevalence** | **Incidence** | **Mortality** | **DALYs** | **Prevalence** | **Incidence** | **Mortality** | **DALYs** |
| **Eastern Europe** | **2010** | 2149·73 | 11·47 | 16·25 | 612·99 | 947·16 | 5·03 | 5·76 | 226·20 |
| | | (1905·82; 2382·68) | ( 7·34;16·64) | (13·65;19·26) | (510·57; 729·24) | ( 834·51;1053·08) | (3·22; 7·31) | (4·87; 6·77) | (187·93;268·04) |
| | **2019** | 1586·03 | 7·42 | 9·64 | 357·40 | 669·70 | 3·22 | 3·30 | 129·57 |
| | | (1410·68; 1756·46) | ( 4·45;11·25) | ( 7·68;11·78) | (282·32; 443·55) | ( 592·79; 745·56) | (1·99; 4·80) | (2·63; 4·08) | (102·01;160·75) |
| | **Change (%) 2010-2019** | -26·22 | -35·28 | -40·72 | -41·70 | -29·29 | -36·02 | -42·66 | -42·72 |
| | | (-28·55;-23·59) | (-42·14;-28·81) | (-47·09;-33·60) | (-48·06;-34·65) | (-31·69;-26·74) | (-40·94;-31·62) | (-48·86;-35·93) | (-49·03;-35·99) |
| **Central Europe** | **2010** | 1083·41 | 8·28 | 7·45 | 231·53 | 798·16 | 6·25 | 4·30 | 140·45 |
| | | ( 978·75; 1182·87) | ( 5·79;11·19) | ( 5·70; 9·42) | (176·77; 292·76) | ( 716·70; 877·45) | (4·38; 8·49) | (3·34; 5·39) | (108·30;175·97) |
| | **2019** | 896·37 | 6·92 | 6·26 | 187·71 | 632·53 | 5·09 | 3·39 | 110·21 |
| | | ( 808·18; 976·35) | ( 4·81; 9·53) | ( 4·69; 8·27) | (140·91; 247·27) | ( 569·15; 690·78) | (3·56; 6·92) | (2·58; 4·45) | ( 83·57;143·53) |
| | **Change (%) 2010-2019** | -17·26 | -16·35 | -15·99 | -18·93 | -20·75 | -18·60 | -21·12 | -21·53 |
| | | (-19·26;-15·08) | (-22·68; -9·77) | (-27·38; -4·32) | (-29·60; -7·60) | (-22·56;-18·72) | (-24·65;-12·72) | (-31·63;-10·57) | (-32·10;-10·74) |
| **Western Europe** | **2010** | 3623·19 | 12·40 | 6·45 | 173·91 | 756·12 | 2·45 | 0·93 | 27·87 |
| | | (3265·01; 3962·98) | ( 8·78;16·78) | ( 4·91; 8·29) | (130·58; 227·54) | ( 677·75; 831·04) | (1·74; 3·31) | (0·71; 1·19) | ( 20·96; 36·77) |
| | **2019** | 3383·20 | 9·32 | 5·62 | 142·00 | 643·72 | 1·90 | 0·71 | 20·97 |
| | | (3041·50; 3696·22) | ( 6·69;12·50) | ( 4·29; 7·25) | (108·66; 184·71) | ( 577·98; 706·76) | (1·36; 2·53) | (0·54; 0·92) | ( 16·00; 27·47) |
| | **Change (%) 2010-2019** | -6·62 | -24·84 | -12·93 | -18·35 | -14·87 | -22·46 | -23·36 | -24·77 |
| | | ( -8·24; -4·64) | (-29·93;-19·35) | (-16·86; -8·76) | (-21·95;-14·51) | (-16·29;-13·03) | (-27·21;-17·69) | (-26·46;-20·11) | (-27·93;-21·54) |

| | Year | Prevalence | Incidence | Mortality | DALYs | Liver cancer due to hepatitis Prevalence | Incidence | Mortality | DALYs |
|---|---|---|---|---|---|---|---|---|---|
| **Eastern Europe** | **2010** | 2·36<br>( 2·02; 2·74) | 2·10<br>(1·79; 2·45) | 2·05<br>(1·75; 2·40) | 61·46<br>( 52·50; 71·19) | 0·81<br>(0·69;0·93) | 0·70<br>(0·60;0·82) | 0·68<br>(0·58;0·79) | 21·17<br>(18·26;24·48) |
| | **2019** | 2·46<br>( 1·96; 3·09) | 2·16<br>(1·72; 2·69) | 2·12<br>(1·68; 2·65) | 61·33<br>( 48·87; 76·78) | 0·80<br>(0·65;1·00) | 0·68<br>(0·55;0·85) | 0·65<br>(0·52;0·81) | 20·39<br>(16·49;25·25) |
| | **Change (%) 2010-2019** | 4·25<br>(-12·35; 24·59) | 2·90<br>(-13·56; 22·48) | 2·98<br>(-12·59; 24·35) | -0·21<br>(-15·86; 22·17) | -0·11<br>(-16·08; 19·21) | -2·81<br>(-18·02; 15·73) | -3·44<br>(-17·65; 16·88) | -3·71<br>(-19·14; 17·91) |
| **Central Europe** | **2010** | 1·65<br>( 1·23; 2·18) | 1·47<br>(1·08; 1·95) | 1·47<br>(1·08; 1·96) | 38·82<br>( 29·33; 50·98) | 0·94<br>(0·71;1·22) | 0·82<br>(0·61;1·07) | 0·80<br>(0·60;1·06) | 22·44<br>(17·14;29·10) |
| | **2019** | 1·54<br>( 1·10; 2·14) | 1·37<br>(0·98; 1·91) | 1·37<br>(0·97; 1·91) | 34·88<br>( 25·57; 48·00) | 0·82<br>(0·60;1·12) | 0·70<br>(0·51;0·96) | 0·68<br>(0·50;0·94) | 19·05<br>(14·11;25·94) |
| | **Change (%) 2010-2019** | -6·46<br>(-19·99; 9·23) | -6·77<br>(-20·02; 8·48) | -6·45<br>(-20·16; 9·00) | -10·16<br>(-24·05; 5·33) | -12·21<br>(-24·80; 3·34) | -14·04<br>(-26·04; 0·84) | -14·71<br>(-27·19; -0·76) | -15·09<br>(-28·16; -0·34) |
| **Western Europe** | **2010** | 8·10<br>( 6·30;10·38) | 4·73<br>(3·58; 6·17) | 3·82<br>(2·84; 5·09) | 95·12<br>( 72·51;123·73) | 1·29<br>(1·02;1·62) | 0·70<br>(0·54;0·90) | 0·53<br>(0·40;0·70) | 14·84<br>(11·47;18·99) |
| | **2019** | 9·30<br>( 6·87;12·63) | 5·34<br>(3·87; 7·41) | 4·30<br>(3·13; 5·82) | 103·30<br>( 76·20;137·07) | 1·36<br>(1·02;1·80) | 0·71<br>(0·52;0·97) | 0·53<br>(0·39;0·71) | 14·60<br>(11·09;18·96) |
| | **Change (%) 2010-2019** | 14·72<br>( -1·28; 34·47) | 12·94<br>( -2·38; 32·61) | 12·60<br>( 6·09; 20·09) | 8·60<br>( 1·48; 16·80) | 5·30<br>( -9·44; 23·34) | 1·39<br>(-12·83; 19·52) | -0·49<br>( -6·29; 6·33) | -1·59<br>( -7·62; 5·54) |

UI= Uncertainty Interval

**Supplemental Table C·** Prevalence, incidence, mortality and DALYs attributable to HBV in 2010 and 2019, by Central Europe countries·

| | | N in thousands (95%UI) | | N (95%UI) | | Age- standardized rate x 100,000 (95%UI) | | | |
|---|---|---|---|---|---|---|---|---|---|
| | **Year** | **Prevalenze** | **Incidence** | **Mortality** | **DALYs** | **Prevalence** | **Incidence** | **Mortality** | **DALYs** |
| **Acute hepatitis** | | | | | | | | | |
| **Albania** | **2010** | 0·81 (0·66;0·99) | 7·05 (5·68;8·58) | 0·54 (0·43;0·68) | 35·41 (27·47; 45·37) | 26·48 (21·21;32·59) | 229·51 (183·84;282·42) | 0·0166 (0·0134;0·0209) | 1·15 (0·89;1·49) |
| | **2019** | 0·63 (0·51;0·76) | 5·42 (4·42;6·60) | 0·54 (0·38;0·75) | 32·02 (24·29; 42·12) | 19·38 (15·68;23·99) | 167·99 (135·87;207·95) | 0·0150 (0·0106;0·0207) | 1·01 (0·76;1·33) |
| | **Change (%) 2010-2019** | -23·13 (-39·28;-3·31) | -23·13 (-39·28;-3·31) | -0·14 (-28·05;36·12) | -9·56 (-27·34;15·06) | -26·80 (-42·21;-7·52) | -26·80 (-42·21;-7·52) | -9·70 (-34·24;22·21) | -12·16 (-29·70;11·49) |
| **Bosnia and Herzegovina** | **2010** | 1·07 (0·89;1·27) | 9·26 (7·69;10·98) | 4·13 (3·41;5·53) | 163·92 (139·65; 204·31) | 25·47 (21·18;30·25) | 220·75 (183·53;262·14) | 0·0835 (0·0700;0·1099) | 3·66 (3·10;4·64) |
| | **2019** | 0·83 (0·69;1·00) | 7·20 (6·01;8·69) | 2·75 (2·02;3·74) | 104·60 (81·31; 133·34) | 21·53 (17·81;26·00) | 186·61 (154·32;225·35) | 0·0550 (0·0405;0·0731) | 2·47 (1·92;3·17) |
| | **Change (%) 2010-2019** | -22·24 (-35·99;-2·88) | -22·24 (-35·99;-2·88) | -33·25 (-50·53;-10·80) | -36·19 (-49·96;-18·52) | -15·46 (-30·58;5·53) | -15·46 (-30·58;5·53) | -34·12 (-50·38;-13·14) | -32·47 (-47·12;-13·47) |
| **Bulgaria** | **2010** | 5·55 (4·52;6·97) | 48·13 (39·17;60·42) | 8·74 (6·36;10·35) | 459·00 (335·68; 566·63) | 66·06 (53·82;81·90) | 572·49 (466·42;709·77) | 0·0971 (0·0656;0·1224) | 6·16 (4·07;8·09) |
| | **2019** | 4·08 (3·14;5·19) | 35·39 (27·25;44·96) | 7·09 (4·90;9·62) | 348·30 (254·52; 463·02) | 47·63 (37·77;59·54) | 412·76 (327·31;515·98) | 0·0794 (0·0528;0·1142) | 4·82 (3·27;6·82) |
| | **Change (%) 2010-2019** | -26·46 (-43·80;-5·96) | -26·46 (-43·80;-5·96) | -18·85 (-38·34;5·90) | -24·12 (-40·44;-3·86) | -27·90 (-44·03;-7·35) | -27·90 (-44·03;-7·35) | -18·25 (-39·40;9·73) | -21·64 (-42·47;4·23) |
| **Croatia** | **2010** | 1·78 (1·41;2·21) | 15·46 (12·19;19·18) | 1·28 (1·15;1·44) | 75·24 (60·12; 94·95) | 37·20 (29·17;46·34) | 322·37 (252·79;401·60) | 0·0199 (0·0181;0·0220) | 1·39 (1·11;1·76) |
| | **2019** | 1·45 (1·15;1·80) | 12·54 (10·01;15·61) | 1·05 (0·79;1·39) | 63·34 (47·63; 82·82) | 28·45 (22·63;35·27) | 246·57 (196·08;305·69) | 0·0158 (0·0118;0·0210) | 1·16 (0·86;1·50) |
| | **Change (%) 2010-2019** | -18·89 (-35·88;1·87) | -18·89 (-35·88;1·87) | -17·71 (-38·21;10·02) | -15·81 (-30·48;1·82) | -23·51 (-39·40;-3·70) | -23·51 (-39·40;-3·70) | -20·86 (-40·83;5·89) | -17·12 (-32·12;0·45) |
| **Czechia** | **2010** | 2·77 (2·10;3·57) | 24·02 (18·22;30·94) | 2·93 (2·58;3·47) | 144·60 (120·36; 179·36) | 25·69 (19·66;32·68) | 222·69 (170·40;283·26) | 0·0191 (0·0171;0·0223) | 1·11 (0·92;1·39) |
| | **2019** | 2·35 (1·80;2·99) | 20·33 (15·62;25·93) | 2·47 (1·84;3·16) | 123·63 (96·38; 159·02) | 20·45 (15·55;26·07) | 177·20 (134·80;225·94) | 0·0150 (0·0113;0·0189) | 0·93 (0·72;1·19) |
| | **Change (%) 2010-2019** | -15·37 (-34·32;4·99) | -15·37 (-34·32;4·99) | -15·58 (-34·07;6·13) | -14·50 (-29·57;1·66) | -20·43 (-38·36;1·05) | -20·43 (-38·36;1·05) | -21·64 (-38·73;-1·42) | -16·85 (-31·86;-0·78) |
| **Hungary** | **2010** | 4·37 (3·80;5·15) | 37·90 (32·90;44·59) | 2·28 (1·90;2·83) | 157·58 (120·88; 204·52) | 41·55 (36·05;48·56) | 360·09 (312·40;420·84) | 0·0162 (0·0136;0·0197) | 1·32 (1·01;1·69) |
| | **2019** | 4·17 (3·63;4·85) | 36·12 (31·47;42·03) | 2·12 (1·53;2·92) | 148·55 (111·36; 192·91) | 40·94 (35·82;46·78) | 354·82 (310·42;405·46) | 0·0148 (0·0109;0·0195) | 1·27 (0·96;1·64) |
| | **Change (%) 2010-2019** | -4·69 (-20·03;13·20) | -4·69 (-20·03;13·20) | -6·91 (-27·15;16·96) | -5·73 (-19·27;11·66) | -1·46 (-17·50;17·20) | -1·46 (-17·50;17·20) | -8·64 (-27·18;13·73) | -3·83 (-18·30;14·46) |
| **Montenegro** | **2010** | 0·14 | 1·18 | 0·10 | 5·90 | 21·33 | 184·88 | 0·0125 | 0·86 |

| | | | | | | | | | |
|---|---|---|---|---|---|---|---|---|---|
| | | (0·11;0·17) | (0·92;1·46) | (0·08;0·11) | (4·78; 7·40) | (16·70;26·41) | (144·73;228·92) | (0·0107;0·0146) | (0·70;1·08) |
| | **2019** | 0·12 (0·09;0·14) | 1·01 (0·77;1·25) | 0·09 (0·07;0·11) | 5·27 (4·04; 6·71) | 17·83 (13·76;22·28) | 154·50 (119·22;193·12) | 0·0109 (0·0084;0·0138) | 0·74 (0·57;0·95) |
| | **Change (%) 2010-2019** | -14·23 (-30·37;5·51) | -14·23 (-30·37;5·51) | -6·80 (-27·01;17·44) | -10·60 (-23·95;6·36) | -16·43 (-32·27;1·83) | -16·43 (-32·27;1·83) | -12·83 (-31·21;9·31) | -13·63 (-26·72;4·33) |
| **North Macedonia** | **2010** | 0·73 (0·59;0·88) | 6·32 (5·08;7·59) | 1·23 (1·10;1·38) | 57·55 (49·78; 66·45) | 31·82 (25·79;38·53) | 275·75 (223·55;333·97) | 0·0487 (0·0432;0·0545) | 2·54 (2·16;2·98) |
| | **2019** | 0·68 (0·54;0·82) | 5·85 (4·65;7·07) | 1·1 8(0·89;1·53) | 53·83 (42·24; 68·34) | 27·29 (21·78;33·25) | 236·54 (188·77;288·20) | 0·0416 (0·0311;0·0541) | 2·20 (1·69;2·84) |
| | **Change (%) 2010-2019** | -7·39 (-24·76;14·49) | -7·39 (-24·76;14·49) | -4·20 (-28·19;25·50) | -6·46 (-24·54;17·03) | -14·22 (-30·47;5·47) | -14·22 (-30·47;5·47) | -14·70 (-35·64;12·67) | -13·44 (-32·43;11·97) |
| **Poland** | **2010** | 7·79 (6·23;9·57) | 67·55 (54·03;82·98) | 9·61 (8·38;10·29) | 494·00 (422·10; 580·87) | 18·00 (14·48;22·18) | 156·03 (125·49;192·25) | 0·0204 (0·0176;0·0222) | 1·23 (1·03;1·45) |
| | **2019** | 5·15 (3·89;6·53) | 44·67 (33·71;56·61) | 9·30 (7·13;11·41) | 415·60 (336·39; 511·18) | 11·23 (8·58;14·28) | 97·34 (74·34;123·80) | 0·0177 (0·0134;0·0217) | 0·98 (0·77;1·20) |
| | **Change (%) 2010-2019** | -33·86 (-46·26;-18·73) | -33·86 (-46·26;-18·73) | -3·22 (-20·78;16·52) | -15·87 (-28·40;-1·60) | -37·62 (-49·37;-22·58) | -37·62 (-49·37;-22·58) | -13·52 (-30·00;4·04) | -20·64 (-34·00;-4·64) |
| **Romania** | **2010** | 7·50 (5·79;9·49) | 65·01 (50·14;82·22) | 5·26 (4·45;6·01) | 369·81 (294·98; 462·85) | 31·85 (24·08;40·34) | 276·00 (208·68;349·59) | 0·0219 (0·0179;0·0252) | 1·69 (1·33;2·11) |
| | **2019** | 6·10 (4·82;7·58) | 52·89 (41·78;65·69) | 4·31 (3·35;5·59) | 304·17 (231·72; 395·16) | 27·46 (21·84;34·06) | 238·03 (189·24;295·18) | 0·0192 (0·0147;0·0248) | 1·53 (1·16;1·98) |
| | **Change (%) 2010-2019** | -18·65 (-36·22;4·19) | -18·65 (-36·22;4·19) | -18·18 (-35·54;2·40) | -17·75 (-32·11;-2·06) | -13·76 (-32·82;12·05) | -13·76 (-32·82;12·05) | -12·21 (-31·03;11·64) | -9·63 (-26·55;11·58) |
| **Serbia** | **2010** | 2·26 (1·78;2·85) | 19·56 (15·42;24·74) | 5·07 (4·61;5·55) | 214·73 (192·07; 243·00) | 24·52 (19·49;30·91) | 212·46 (168·94;267·85) | 0·0423 (0·0386;0·0464) | 2·09 (1·86;2·38) |
| | **2019** | 1·89 (1·42;2·42) | 16·35 (12·32;21·01) | 4·09 (3·12;5·21) | 171·47 (136·70; 215·03) | 20·13 (15·23;25·92) | 174·48 (131·96;224·68) | 0·0332 (0·0255;0·0422) | 1·67 (1·33;2·10) |
| | **Change (%) 2010-2019** | -16·43 (-34·39;6·74) | -16·43 (-34·39;6·74) | -19·30 (-38·52;3·84) | -20·14 (-35·77;-0·94) | -17·88 (-36·42;5·49) | -17·88 (-36·42;5·49) | -21·59 (-40·47;2·21) | -20·51 (-35·83;-0·85) |
| **Slovakia** | **2010** | 1·81 (1·41;2·29) | 15·64 (12·18;19·87) | 1·61 (1·46;1·77) | 90·05 (74·28; 111·74) | 29·97 (23·34;38·19) | 259·73 (202·29;330·95) | 0·0239 (0·0215;0·0263) | 1·48 (1·22;1·82) |
| | **2019** | 1·47 (1·06;1·87) | 12·70 (9·15;16·23) | 1·48 (1·10;1·92) | 80·24 (61·23; 102·84) | 22·63 (16·61;28·90) | 196·17 (143·94;250·45) | 0·0197 (0·0144;0·0257) | 1·25 (0·95;1·61) |
| | **Change (%) 2010-2019** | -18·84 (-36·92;4·93) | -18·84 (-36·92;4·93) | -8·21 (-30·54;17·75) | -10·89 (-27·19;8·47) | -24·47 (-41·09;-2·57) | -24·47 (-41·09;-2·57) | -17·55 (-38·51;6·29) | -15·93 (-32·72;5·24) |
| **Slovenia** | **2010** | 0·77 (0·63;0·95) | 6·71 (5·43;8·26) | 0·72 (0·62;0·85) | 38·04 (31·32; 46·97) | 34·31 (27·91;42·40) | 297·32 (241·85;367·47) | 0·0239 (0·0212;0·0275) | 1·47 (1·21;1·82) |
| | **2019** | 0·65 (0·51;0·80) | 5·60 (4·40;6·96) | 0·58 (0·42;0·77) | 31·50 (23·67; 41·46) | 26·45 (20·70;33·13) | 229·22 (179·41;287·10) | 0·0174 (0·0129;0·0228) | 1·16 (0·89;1·51) |
| | **Change (%) 2010-2019** | -16·44 (-33·03;5·47) | -16·44 (-33·03;5·47) | -19·31 (-38·90;7·67) | -17·17 (-32·87;2·02) | -22·90 (-38·59;-1·34) | -22·90 (-38·59;-1·34) | -27·13 (-44·70;-2·23) | -21·45 (-36·87;-2·99) |
| **Cirrhosis and other chronic liver diseases due to hepatitis** | | | | | | | | | |
| **Albania** | **2010** | 21·29 | 0·11 | 47·59 | 1298·32 | 678·87 | 3·37 | 1·4110 | 38·07 |

| | | | | | | | | | |
|---|---|---|---|---|---|---|---|---|---|
| | | (18·34;24·29) | (0·08;0·16) | (32·88; 65·73) | (898·51; 1782·04) | (583·19;778·15) | (2·26;4·80) | (0·9930; 1·9598) | (26·73;51·85) |
| | **2019** | 17·37 (15·45;19·44) | 0·10 (0·07;0·15) | 54·75 (34·85; 82·22) | 1373·74 (885·91; 2055·01) | 520·42 (460·40;585·33) | 3·42 (2·33;4·86) | 1·3531 (0·8761; 2·0089) | 36·48 (23·43;53·64) |
| | **Change (%) 2010-2019** | -18·40 (-24·72;-11·09) | -7·54 (-17·48;3·32) | 137·86 (98·30;185·67) | 5·81 (-22·25;39·57) | -23·34 (-29·46;-16·26) | 1·59 (-8·29;11·83) | -4·10 (-28·17;24·10) | -4·17 (-28·63;25·69) |
| **Bosnia and Herzegovina** | **2010** | 24·32 (21·16;27·51) | 0·23 (0·16;0·32) | 107·81 (70·92;158·11) | 3967·39 (2870·66; 5295·00) | 569·86 (493·82;648·93) | 4·86 (3·36;6·68) | 2·5122 (1·8106; 3·3674) | 73·80 (54·34;97·84) |
| | **2019** | 19·37 (17·04;21·75) | 0·15 (0·10;0·21) | 0·11 (0·07;0·16) | 2985·02 (1964·82; 4361·88) | 486·03 (424·28;547·60) | 3·66 (2·45;5·17) | 1·9139 (1·2772; 2·7773) | 56·30 (37·85;80·89) |
| | **Change (%) 2010-2019** | -20·35 (-26·48;-13·81) | -35·89 (-42·23;-27·94) | -21·80 (-39·81;2·55) | -24·76 (-42·74;-0·81) | -14·71 (-21·41;-7·63) | -24·70 (-31·53;-15·64) | -23·81 (-41·60;-1·27) | -23·71 (-41·76;-0·27) |
| **Bulgaria** | **2010** | 215·19 (208·17;221·62) | 0·46 (0·29;0·68) | 485·39 (347·54;649·95) | 15146·52 (10789·79; 20423·00) | 2331·29 (2266·72;2396·80) | 5·49 (3·50;8·05) | 4·122 0(2·9754; 5·5054) | 139·21 (99·75;188·58) |
| | **2019** | 168·84 (161·14;174·76) | 0·45 (0·29;0·64) | 501·86 (334·53;713·31) | 15373·27 (10214·76; 22279·24) | 1762·43 (1697·81;1815·62) | 5·59 (3·63;8·06) | 4·3074 (2·8834; 6·2424) | 145·58 (95·23;214·18) |
| | **Change (%) 2010-2019** | -21·54 (-23·60;-19·86) | -2·98 (-14·47;9·24) | 3·39 (-21·17;33·40) | 1·50 (-23·33;32·24) | -24·40 (-26·20;-22·84) | 1·77 (-9·74;14·50) | 4·50 (-20·87;35·75) | 4·58 (-20·99;36·06) |
| **Croatia** | **2010** | 56·06 (51·47;60·73) | 0·35 (0·23;0·50) | 242·44 (173·13;327·68) | 6930·72 (4961·91;9355·57) | 1123·08 (1029·82;1221·66) | 6·73 (4·37;9·66) | 3·3952 (2·4445; 4·5656) | 104·06 (74·06;140·23) |
| | **2019** | 46·03 (41·87;49·84) | 0·28 (0·19;0·40) | 201·0 2(132·42;289·62) | 5454·88 (3615·10; 7840·85) | 856·19 (777·65;927·85) | 5·70 (3·77;8·12) | 2·6399 (1·7518; 3·7541) | 79·36 (52·85;113·63) |
| | **Change (%) 2010-2019** | -17·89 (-22·51;-13·13) | -19·10 (-26·61;-11·10) | -17·08 (-36·42;6·58) | -21·29 (-39·59;2·02) | -23·76 (-28·02;-19·24) | -15·25 (-23·20;-6·97) | -22·24 (-40·14;0·31) | -23·74 (-41·20;-0·39) |
| **Czechia** | **2010** | 84·66 (71·68;95·47) | 0·84 (0·59;1·13) | 703·53 (523·44;893·89) | 21821·47 (16231·99; 27665·63) | 737·49 (622·33;837·54) | 7·01 (4·98;9·40) | 4·3614 (3·2716; 5·4896) | 143·01 (107·58;179·98) |
| | **2019** | 75·05 (65·35;83·84) | 0·45 (0·29;0·67) | 396·12 (269·75;561·61) | 11891·68 (8145·23; 16734·82) | 594·19 (515·58;663·96) | 3·53 (2·28;5·16) | 2·2682 (1·5739; 3·2098) | 73·93 (49·87;105·49) |
| | **Change (%) 2010-2019** | -11·36 (-18·40;-2·24) | -46·30 (-55·09;-34·47) | -43·69 (-56·45;-29·00) | -45·50 (-57·96;-29·82) | -19·43 (-25·88;-10·22) | -49·61 (-57·18;-39·23) | -47·99 (-59·71;-33·12) | -48·30 (-59·96;-32·93) |
| **Hungary** | **2010** | 74·72 (67·21;83·15) | 1·25 (0·86;1·66) | 1291·81 (967·86;1638·97) | 40584·94 (30372·19; 51479·03) | 698·59 (631·44;777·35) | 11·27 (7·79;14·97) | 8·3995 (6·3514;10·6105) | 278·98 (210·53;351·28) |
| | **2019** | 68·45 (62·73;75·22) | 1·11 (0·78;1·45) | 1009·33 (727·35;1357·55) | 29906·35 (21629·45; 40159·48) | 668·66 (610·96;738·97) | 9·14 (6·50;11·95) | 6·1729 (4·4547; 8·2848) | 197·41 (142·63;264·19) |
| | **Change (%) 2010-2019** | -8·39 (-13·51;-2·39) | -11·70 (-24·28;2·01) | -21·87 (-36·78;-4·32) | -26·31 (-40·32;-9·58) | -4·29 (-9·73;2·26) | -18·89 (-29·02;-8·10) | -26·51 (-40·37;-10·02) | -29·24 (-42·63;-13·35) |
| **Montenegro** | **2010** | 4·42 (3·76;5·11) | 0·02 (0·01;0·02) | 14·65 (10·77; 19·05) | 462·27 (337·18; 597·19) | 676·69 (566·93;787·48) | 2·71 (1·88;3·62) | 1·7261 (1·2890; 2·2240) | 55·90 (41·21;71·96) |
| | **2019** | 3·85 (3·28;4·36) | 0·01 (0·01;0·02) | 9·36 (6·23; 13·68) | 288·83 (191·80; 425·41) | 562·50 (478·35;641·15) | 1·65 (1·08;2·41) | 1·0244 (0·6884; 1·4932) | 33·19 (22·01;48·89) |
| | **Change (%) 2010-2019** | -13·04 (-20·59;-4·92) | -39·17 (-48·07;-26·78) | -36·12 (-50·62;-18·11) | -37·52 (-52·31;-18·39) | -16·87 (-24·33;-8·64) | -38·96 (-47·88;-26·95) | -40·65 (-54·18;-23·82) | -40·63 (-54·75;-23·00) |
| **North** | **2010** | 19·01 (16·08;21·73) | 0·15 (0·11;0·19) | 87·54 (65·80;113·34) | 2567·15 (1921·15; 3285·07) | 822·65 (691·63;950·32) | 5·71 (4·10;7·54) | 3·1996 (2·4443; 4·1228) | 92·09 (69·38;117·87) |

| | | | | | | | | | |
|---|---|---|---|---|---|---|---|---|---|
| **Macedonia** | **2019** | 17·52<br>(15·14;19·65) | 0·09<br>(0·06;0·13) | 59·47<br>(39·00; 85·80) | 1724·52<br>(1117·86; 2493·61) | 689·53<br>(592·48;775·59) | 3·47<br>(2·26;4·94) | 1·8965<br>(1·2693; 2·7153) | 55·51<br>(36·55;79·72) |
| | **Change (%) 2010-2019** | -7·84<br>(-15·48;0·92) | -37·05<br>(-46·44;-24·07) | -32·06<br>(-48·53;-10·93) | -32·82<br>(-50·01;-11·83) | -16·18<br>(-23·00;-8·43) | -39·21<br>(-48·35;-26·68) | -40·73<br>(-55·03;-22·80) | -39·73<br>(-55·02;-20·37) |
| **Poland** | **2010** | 217·94<br>(191·54;243·46) | 1·52<br>(1·03;2·10) | 1593·90<br>(1349·19;1865·80) | 53255·08<br>(44495·32; 63193·54) | 485·16<br>(426·03;543·94) | 3·66<br>(2·49;5·10) | 2·9464<br>(2·5040; 3·4522) | 102·80<br>(86·31;121·45) |
| | **2019** | 170·09<br>(150·63;189·96) | 1·35<br>(0·84;1·94) | 1485·35<br>(1150·20;1900·16) | 48197·80<br>(37014·88; 62026·46) | 347·28<br>(307·97;388·64) | 3·01<br>(1·92;4·29) | 2·5458<br>(1·9538; 3·2744) | 88·85<br>(68·24;114·41) |
| | **Change (%) 2010-2019** | -21·96<br>(-24·10;-19·67) | -11·40<br>(-21·58;-3·34) | -6·81<br>(-23·76;13·49) | -9·50<br>(-25·64;10·41) | -28·42<br>(-30·35;-26·40) | -17·65<br>(-26·36;-11·42) | -13·60<br>(-28·99;4·81) | -13·57<br>(-28·77;4·92) |
| **Romania** | **2010** | 221·90<br>(190·34;252·53) | 2·24<br>(1·48;3·29) | 1960·05<br>(1361·41;2627·36) | 58699·07<br>(41741·44; 78750·62) | 902·24<br>(770·67;1022·36) | 9·26<br>(6·07;13·63) | 6·3234<br>(4·5136; 8·4083) | 201·39<br>(143·94;271·30) |
| | **2019** | 186·87<br>(159·01;212·67) | 2·16<br>(1·41;3·17) | 1789·28<br>(1215·01;2568·56) | 51345·45<br>(34873·31; 74510·52) | 789·77<br>(672·38;902·26) | 8·87<br>(5·87;12·88) | 5·5222<br>(3·7373; 7·9891) | 172·56<br>(115·34;253·42) |
| | **Change (%) 2010-2019** | -15·78<br>(-23·24;-6·30) | -3·50<br>(-17·67;10·45) | -8·71<br>(-25·88;11·43) | -12·53<br>(-29·60;7·16) | -12·47<br>(-20·17;-2·08) | -4·22<br>(-16·58;8·68) | -12·67<br>(-29·47;6·71) | -14·32<br>(-30·97;4·79) |
| **Serbia** | **2010** | 73·04<br>(61·48;83·59) | 0·56<br>(0·40;0·74) | 444·50<br>(333·98;581·86) | 12619·68<br>(9467·82; 16298·27) | 762·37<br>(639·46;883·32) | 5·64<br>(4·04;7·44) | 3·2256<br>(2·4503; 4·1823) | 96·56<br>(72·10;123·53) |
| | **2019** | 61·34<br>(52·16;70·46) | 0·28<br>(0·18;0·41) | 243·81<br>(160·35;357·56) | 6820·37<br>(4521·82; 9929·29) | 620·29<br>(523·99;714·07) | 2·91<br>(1·89;4·19) | 1·731<br>0(1·1647; 2·5277) | 52·66<br>(35·47;77·32) |
| | **Change (%) 2010-2019** | -16·01<br>(-23·45;-7·14) | -49·65<br>(-57·25;-40·00) | -45·15<br>(-58·81;-27·96) | -45·95<br>(-60·02;-28·38) | -18·64<br>(-25·94;-9·63) | -48·30<br>(-55·62;-38·52) | -46·34<br>(-59·59;-29·11) | -45·46<br>(-59·54;-26·67) |
| **Slovakia** | **2010** | 51·11<br>(43·55;57·99) | 0·37<br>(0·23;0·56) | 320·26<br>(223·95;441·13) | 10656·86<br>(7289·51; 14756·23) | 814·17<br>(690·33;933·89) | 6·02<br>(3·79;9·14) | 4·2639<br>(3·0212; 5·8337) | 145·12<br>(100·33;198·28) |
| | **2019** | 44·10<br>(37·54;50·43) | 0·35<br>(0·22;0·52) | 298·28<br>(193·71;438·78) | 9547·50<br>(6235·87; 14114·20) | 637·45<br>(540·15;730·84) | 5·32<br>(3·39;7·86) | 3·5762<br>(2·3379; 5·2724) | 120·44<br>(78·63;179·02) |
| | **Change (%) 2010-2019** | -13·71<br>(-21·22;-6·30) | -4·75<br>(-15·52;7·85) | -6·86<br>(-30·28;24·16) | -10·41<br>(-32·76;19·91) | -21·71<br>(-28·67;-15·16) | -11·64<br>(-21·13;-0·87) | -16·13<br>(-36·62;11·53) | -17·00<br>(-37·40;10·99) |
| **Slovenia** | **2010** | 19·75<br>(17·17;22·64) | 0·17<br>(0·11;0·25) | 119·34<br>(84·77;161·16) | 3524·10<br>(2492·34; 4805·53) | 824·62<br>(705·98;947·72) | 6·77<br>(4·37;9·77) | 3·6519<br>(2·5933; 4·9121) | 113·91<br>(80·94;154·29) |
| | **2019** | 17·49<br>(15·22;19·71) | 0·13<br>(0·09;0·19) | 101·02<br>(64·97;152·18) | 2804·70<br>(1800·49; 4211·60) | 663·04<br>(577·59;745·00) | 5·41<br>(3·54;7·77) | 2·7249<br>(1·7446; 4·1102) | 83·17<br>(53·24;125·41) |
| | **Change (%) 2010-2019** | -11·44<br>(-19·23;-2·69) | -21·34<br>(-29·80;-11·95) | -15·35<br>(-35·99;12·03) | -20·41<br>(-40·14;5·27) | -19·59<br>(-26·47;-11·94) | -20·14<br>(-29·26;-11·08) | -25·38<br>(-44·01;-0·91) | -26·99<br>(-45·16;-2·88) |
| **Liver cancer due to hepatitis** | | | | | | | | | |
| **Albania** | **2010** | 0·06<br>(0·04;0·08) | 0·05<br>(0·04;0·07) | 51·48<br>(34·62; 73·35) | 1442·77<br>(1011·55; 1967·41) | 1·69<br>(1·18;2·30) | 1·48<br>(1·03;2·06) | 1·4783<br>(1·0119;2·0824) | 41·91<br>(29·67;56·35) |
| | **2019** | 0·08<br>(0·05;0·11) | 0·07<br>(0·04;0·10) | 68·08<br>(42·30;102·53) | 1786·10<br>(1131·98; 2672·56) | 1·91<br>(1·23;2·84) | 1·66<br>(1·06;2·45) | 1·6403<br>(1·0440;2·4364) | 46·12<br>(30·09;68·18) |
| | **Change (%) 2010-2019** | 30·48<br>(-7·01;74·75) | 31·37<br>(-6·22;76·20) | 32·24<br>(-4·64;76·96) | 23·80<br>(-11·78;64·71) | 13·45<br>(-18·07;51·85) | 11·83<br>(-18·87;49·22) | 10·96<br>(-19·10;47·55) | 10·05<br>(-20·76;47·05) |
| **Bosnia and** | **2010** | 0·11<br>(0·08;0·16) | 0·11<br>(0·07;0·15) | 107·79<br>(74·68;151·78) | 2791·22<br>(2006·51; 3771·86) | 2·06<br>(1·49;2·80) | 1·90<br>(1·36;2·59) | 1·9322<br>(1·3808;2·6909) | 50·74<br>(36·94;67·72) |

| | | | | | | | | | |
|---|---|---|---|---|---|---|---|---|---|
| **Herzegovina** | **2019** | 0·10 (0·07;0·16) | 0·10 (0·06;0·15) | 99·36 (64·38;150·74) | 2480·08 (1624·03; 3745·99) | 1·84 (1·22;2·73) | 1·67 (1·11;2·50) | 1·6920 (1·1194;2·5395) | 44·42 (29·68;65·30) |
| | **Change (%) 2010-2019** | -8·35 (-30·81;17·77) | -8·24 (-30·11;17·96) | -7·82 (-29·86;18·50) | -11·15 (-33·16;14·75) | -11·04 (-32·42;14·62) | -12·08 (-32·80;12·85) | -12·43 (-32·84;12·26) | -12·46 (-33·52;13·03) |
| **Bulgaria** | **2010** | 0·18 (0·13;0·25) | 0·17 (0·12;0·23) | 167·44 (117·24;231·74) | 4615·32 (3340·49; 6283·68) | 1·51 (1·11;2·04) | 1·34 (0·97;1·80) | 1·3220 (0·9535;1·7879) | 39·90 (29·33;53·58) |
| | **2019** | 0·14 (0·09;0·21) | 0·13 (0·08;0·19) | 126·05 (83·30;189·12) | 3398·04 (2261·51; 5080·77) | 1·17 (0·79;1·71) | 1·02 (0·68;1·52) | 0·9964 (0·6696;1·4708) | 30·27 (20·40;44·37) |
| | **Change (%) 2010-2019** | -24·37 (-41·78;-3·31) | -24·72 (-41·67;-3·90) | -24·72 (-41·67;-5·31) | -26·37 (-43·82;-5·30) | -22·47 (-40·43;-0·03) | -23·94 (-41·42;-2·46) | -24·63 (-41·87;-3·30) | -24·12 (-42·47;-1·81) |
| **Croatia** | **2010** | 0·09 (0·06;0·12) | 0·07 (0·05;0·10) | 67·03 (46·89; 91·70) | 1677·17 (1199·29; 2280·84) | 1·23 (0·89;1·65) | 0·96 (0·68;1·29) | 0·8855 (0·6275;1·1987) | 23·88 (17·46;31·93) |
| | **2019** | 0·08 (0·05;0·12) | 0·06 (0·04;0·10) | 60·55 (38·70; 90·41) | 1470·86 (951·93; 2204·57) | 1·09 (0·72;1·60) | 0·82 (0·54;1·22) | 0·7404 (0·4876;1·1081) | 19·95 (13·26;29·57) |
| | **Change (%) 2010-2019** | -6·94 (-28·55;22·26) | -9·05 (-29·62;18·52) | -9·67 (-29·99;16·88) | -12·30 (-32·79;14·93) | -11·39 (-32·28;17·45) | -14·87 (-34·09;10·98) | -16·38 (-35·18;8·63) | -16·45 (-35·94;9·70) |
| **Czechia** | **2010** | 0·15 (0·11;0·21) | 0·13 (0·09;0·18) | 128·75 (90·04;178·17) | 3279·17 (2353·32; 4476·01) | 0·89 (0·65;1·21) | 0·76 (0·55;1·04) | 0·7420 (0·5307;1·0206) | 20·03 (14·76;26·81) |
| | **2019** | 0·12 (0·08;0·18) | 0·11 (0·07;0·16) | 106·91 (69·52;160·57) | 2599·62 (1727·38; 3856·63) | 0·66 (0·45;0·98) | 0·56 (0·38;0·82) | 0·5436 (0·3631;0·7987) | 14·66 (9·93;21·43) |
| | **Change (%) 2010-2019** | -18·72 (-36·39;2·43) | -17·88 (-35·48;2·99) | -16·96 (-34·70;3·77) | -20·72 (-37·94;0·19) | -25·79 (-41·60;-5·92) | -26·56 (-42·10;-7·03) | -26·73 (-42·00;-7·38) | -26·81 (-42·77;-6·29) |
| **Hungary** | **2010** | 0·19 (0·14;0·26) | 0·17 (0·12;0·23) | 170·88 (123·56;232·16) | 4546·29 (3283·61; 6073·27) | 1·21 (0·88;1·60) | 1·06 (0·77;1·41) | 1·0383 (0·7583;1·3867) | 29·68 (21·83;39·17) |
| | **2019** | 0·11 (0·08;0·16) | 0·10 (0·07;0·15) | 103·24 (68·63;150·76) | 2540·20 (1717·77; 3676·92) | 0·68 (0·47;0·97) | 0·59 (0·40;0·84) | 0·5759 (0·3939;0·8329) | 15·78 (10·95;22·37) |
| | **Change (%) 2010-2019** | -40·41 (-52·83;-26·26) | -40·24 (-52·82;-26·21) | -39·58 (-52·11;-25·33) | -44·13 (-55·67;-30·12) | -43·56 (-55·52;-29·51) | -44·45 (-56·14;-30·99) | -44·53 (-56·20;-31·07) | -46·82 (-58·30;-33·25) |
| **Montenegro** | **2010** | 0·02 (0·01;0·02) | 0·01 (0·01;0·02) | 13·99 (9·88; 19·44) | 388·36 (285·00; 523·76) | 1·87 (1·38;2·50) | 1·63 (1·17;2·21) | 1·6017 (1·1515;2·1730) | 45·97 (34·54;61·07) |
| | **2019** | 0·01 (0·01;0·02) | 0·01 (0·01;0·02) | 12·17 (8·01; 17·83) | 331·29 (221·79; 482·30) | 1·54 (1·04;2·20) | 1·31 (0·88;1·90) | 1·2775 (0·8545;1·8400) | 36·73 (25·19;52·33) |
| | **Change (%) 2010-2019** | -11·17 (-29·36;10·70) | -12·42 (-30·51;8·67) | -13·03 (-30·76;7·72) | -14·69 (-31·96;5·97) | -17·37 (-34·43;2·91) | -19·26 (-35·70;0·20) | -20·24 (-36·52;-0·84) | -20·10 (-36·46;-0·81) |
| **North Macedonia** | **2010** | 0·07 (0·05;0·10) | 0·07 (0·05;0·09) | 66·78 (46·87; 90·57) | 1827·85 (1334·02; 2466·32) | 2·62 (1·90;3·53) | 2·38 (1·70;3·21) | 2·3990 (1·7123;3·2275) | 64·95 (47·89;86·51) |
| | **2019** | 0·07 (0·05;0·11) | 0·06 (0·04;0·10) | 63·23 (41·09; 94·27) | 1701·21 (1125·78; 2554·59) | 2·20 (1·47;3·26) | 1·96 (1·30;2·89) | 1·9676 (1·2997;2·8988) | 53·51 (35·86;78·52) |
| | **Change (%) 2010-2019** | -4·68 (-27·77;23·37) | -5·23 (-27·87;22·49) | -5·32 (-27·40;22·04) | -6·93 (-29·59;20·47) | -16·31 (-36·16;7·85) | -17·50 (-36·48;5·95) | -17·98 (-36·49;5·09) | -17·60 (-37·15;6·32) |
| **Poland** | **2010** | 0·22 (0·19;0·26) | 0·20 (0·17;0·24) | 210·13 (177·14;248·41) | 5340·16 (4540·23; 6351·70) | 0·39 (0·33;0·46) | 0·36 (0·30;0·42) | 0·3615 (0·3054;0·4262) | 9·60 (8·20;11·31) |
| | **2019** | 0·27 | 0·25 | 260·18 | 6311·92 | 0·43 | 0·38 | 0·3873 | 10·21 |

| | | | | | | | | | |
|---|---|---|---|---|---|---|---|---|---|
| | | (0·21;0·35) | (0·19;0·32) | (204·46;335·61) | (4925·70; 8120·69) | (0·33;0·54) | (0·30;0·49) | (0·3067;0·4961) | (7·99;13·04) |
| | **Change (%) 2010-2019** | 22·72 (1·30;48·01) | 23·15 (2·14;48·26) | 23·82 (2·45;48·98) | 18·20 (-3·00;43·16) | 9·17 (-10·15;32·32) | 7·68 (-10·99;29·82) | 7·14 (-11·10;29·77) | 6·28 (-12·51;28·91) |
| **Romania** | **2010** | 0·21 (0·15;0·29) | 0·19 (0·14;0·26) | 187·62 (134·38;261·25) | 5276·03 (3863·97; 7199·81) | 0·69 (0·51;0·94) | 0·60 (0·45;0·82) | 0·5918 (0·4346;0·8075) | 17·89 (13·27;24·01) |
| | **2019** | 0·23 (0·16;0·34) | 0·21 (0·14;0·31) | 209·26 (140·88;304·68) | 5624·98 (3799·31; 8185·37) | 0·75 (0·51;1·08) | 0·65 (0·44;0·94) | 0·6294 (0·4276;0·9023) | 18·72 (12·76;26·87) |
| | **Change (%) 2010-2019** | 11·61 (-10·67;38·64) | 11·28 (-10·63;37·65) | 11·54 (-10·21;38·22) | 6·61 (-14·69;32·74) | 8·85 (-12·57;35·80) | 7·12 (-13·73;33·19) | 6·35 (-14·45;31·75) | 4·65 (-16·54;31·26) |
| **Serbia** | **2010** | 0·23 (0·16;0·31) | 0·20 (0·14;0·27) | 193·85 (134·68;270·52) | 4972·79 (3566·18; 6777·32) | 1·63 (1·21;2·18) | 1·38 (1·00;1·86) | 1·3401 (0·9614;1·8314) | 36·23 (26·79;48·31) |
| | **2019** | 0·19 (0·12;0·29) | 0·16 (0·10;0·25) | 157·75 (98·38;241·81) | 3958·37 (2495·21; 6130·67) | 1·36 (0·89;2·04) | 1·09 (0·70;1·65) | 1·0393 (0·6681;1·5642) | 28·37 (18·38;43·17) |
| | **Change (%) 2010-2019** | -15·88 (-36·60;10·85) | -17·97 (-37·59;6·85) | -18·62 (-37·84;5·67) | -20·40 (-40·07;4·99) | -16·90 (-37·43;10·19) | -20·79 (-39·67;3·44) | -22·45 (-40·61;0·43) | -21·69 (-41·34;3·52) |
| **Slovakia** | **2010** | 0·07 (0·05;0·10) | 0·06 (0·04;0·09) | 60·29 (42·15; 84·09) | 1655·26 (1191·87; 2296·94) | 0·99 (0·72;1·37) | 0·82 (0·59;1·14) | 0·7897 (0·5570;1·0950) | 22·18 (16·08;30·33) |
| | **2019** | 0·07 (0·05;0·11) | 0·06 (0·04;0·09) | 59·08 (37·00; 89·30) | 1580·63 (1006·80; 2362·27) | 0·87 (0·56;1·28) | 0·70 (0·45;1·04) | 0·6648 (0·4247;0·9867) | 18·86 (12·28;27·62) |
| | **Change (%) 2010-2019** | -0·37 (-25·44;31·14) | -1·68 (-25·96;27·96) | -2·00 (-25·47;27·35) | -4·51 (-28·50;25·46) | -11·66 (-33·33;16·29) | -14·58 (-35·00;11·54) | -15·81 (-36·13;10·01) | -14·96 (-36·09;11·41) |
| **Slovenia** | **2010** | 0·05 (0·03;0·07) | 0·04 (0·03;0·06) | 39·47 (27·41; 54·04) | 1005·03 (710·92; 1353·50) | 1·52 (1·08;2·03) | 1·22 (0·86;1·66) | 1·1526 (0·8093;1·5713) | 31·23 (22·40;41·97) |
| | **2019** | 0·06 (0·04;0·09) | 0·05 (0·03;0·07) | 45·18 (28·51; 67·66) | 1092·01 (679·99; 1635·71) | 1·54 (0·97;2·29) | 1·21 (0·76;1·81) | 1·1290 (0·7132;1·6966) | 30·04 (18·93;44·57) |
| | **Change (%) 2010-2019** | 15·14 (-15·85;54·29) | 14·24 (-16·01;53·19) | 14·47 (-15·04;53·14) | 8·66 (-21·29;46·92) | 1·12 (-26·41;35·58) | -1·23 (-27·32;33·00) | -2·04 (-27·84;31·64) | -3·79 (-30·72;30·37) |

UI= Uncertainty Interval

**Supplement Table D·** Prevalence, incidence, mortality and DALYs attributable to HBV in 2010 and 2019, by Eastern Europe countries·

| | | N in thousands (95%UI) | | N (95%UI) | | Age- standardized rate x 100,000 (95%UI) | | | |
|---|---|---|---|---|---|---|---|---|---|
| | **Year** | **Prevalence** | **Incidence** | **Mortality** | **DALYs** | **Prevalence** | **Incidence** | **Mortality** | **DALYs** |
| **Acute hepatitis** | | | | | | | | | |
| **Belarus** | **2010** | 1·52 (1·03;2·07) | 13·20 (8·92;17·98) | 21·27 (17·99;24·42) | 1049·70 (894·00; 1231·66) | 14·44 (9·84;19·66) | 125·17 (85·28;170·38) | 0·2058 (0·1730;0·2420) | 11·61 (9·58;14·21) |
| | **2019** | 1·16 (0·78;1·59) | 10·03 (6·74;13·82) | 16·44 (8·28;23·19) | 786·63 (388·71; 1146·65) | 10·55 (7·11;14·59) | 91·45 (61·62;126·41) | 0·1587 (0·0771;0·2317) | 8·87 (4·30;13·35) |
| | **Change (%) 2010-2019** | -23·98 (-45·07;3·57) | -23·98 (-45·07;3·57) | -22·69 (-64·03;11·09) | -25·06 (-65·51;8·94) | -26·94 (-47·50;-0·17) | -26·94 (-47·50;-0·17) | -22·88 (-64·92;13·32) | -23·62 (-65·44;15·71) |
| **Estonia** | **2010** | 0·24 (0·17;0·32) | 2·08 (1·51;2·74) | 0·29 (0·24;0·38) | 15·02 (12·46; 18·35) | 17·88 (13·03;23·50) | 154·97 (112·96;203·65) | 0·0166 (0·0140;0·0201) | 0·99 (0·82;1·20) |
| | **2019** | 0·18 (0·12;0·25) | 1·58 (1·07;2·17) | 0·23 (0·14;0·32) | 11·66 (7·87; 15·25) | 13·07 (8·79;18·09) | 113·24 (76·20;156·82) | 0·0127 (0·0076;0·0173) | 0·76 (0·51;0·99) |
| | **Change (%) 2010-2019** | -24·18 (-45·93;2·36) | -24·18 (-45·93;2·36) | -21·33 (-53·97;7·81) | -22·39 (-46·42;-0·65) | -26·92 (-48·33;0·32) | -26·92 (-48·33;0·32) | -23·22 (-56·41;5·82) | -22·63 (-46·74;-1·11) |
| **Latvia** | **2010** | 0·25 (0·16;0·37) | 2·15 (1·41;3·17) | 1·40 (1·17;1·81) | 62·70 (50·24; 83·33) | 11·06 (7·19;16·58) | 95·84 (62·34;143·66) | 0·0613 (0·0484;0·0822) | 3·62 (2·72;5·01) |
| | **2019** | 0·17 (0·11;0·26) | 1·51 (0·97;2·23) | 0·82 (0·52;1·07) | 34·10 (22·34; 45·06) | 8·18 (5·28;12·20) | 70·89 (45·72;105·72) | 0·0345 (0·0207;0·0471) | 1·97 (1·22;2·87) |
| | **Change (%) 2010-2019** | -29·70 (-51·93;-3·10) | -29·70 (-51·93;-3·10) | -41·83 (-65·33;-20·78) | -45·60 (-65·85;-24·08) | -26·03 (-50·47;3·56) | -26·03 (-50·47;3·56) | -43·70 (-67·54;-17·91) | -45·69 (-69·05;-17·30) |
| **Lithuania** | **2010** | 0·54 (0·39;0·72) | 4·69 (3·40;6·28) | 1·21 (1·08;1·39) | 49·66 (43·54; 58·60) | 17·02 (12·26;22·84) | 147·54 (106·25;197·93) | 0·0286 (0·0258;0·0329) | 1·43 (1·25;1·70) |
| | **2019** | 0·36 (0·23;0·50) | 3·15 (2·02;4·32) | 0·81 (0·55;1·04) | 32·10 (22·49; 40·62) | 12·12 (8·00;16·53) | 105·06 (69·31;143·23) | 0·0188 (0·0126;0·0241) | 0·97 (0·67;1·23) |
| | **Change (%) 2010-2019** | -32·75 (-52·16;-8·61) | -32·75 (-52·16;-8·61) | -33·07 (-59·18;-11·40) | -35·37 (-56·71;-18·52) | -28·79 (-50·03;-3·05) | -28·79 (-50·03;-3·05) | -34·34 (-60·25;-13·49) | -32·50 (-54·71;-15·01) |
| **Republic of Moldova** | **2010** | 1·92 (1·54;2·40) | 16·67 (13·35;20·77) | 0·89 (0·70;1·27) | 76·38 (58·31; 102·07) | 43·68 (34·98;54·91) | 378·58 (303·19;475·90) | 0·0201 (0·0160;0·0278) | 1·78 (1·39;2·33) |
| | **2019** | 1·66 (1·32;2·06) | 14·34 (11·44;17·81) | 0·54 (0·43;0·71) | 56·26 (41·28; 76·40) | 37·69 (30·11;46·37) | 326·66 (260·99;401·87) | 0·0122 (0·0098;0·0153) | 1·30 (0·97;1·71) |
| | **Change (%) 2010-2019** | -13·98 (-30·79;9·62) | -13·98 (-30·79;9·62) | -38·87 (-52·39;-25·51) | -26·34 (-40·08;-10·46) | -13·71 (-30·54;9·46) | -13·71 (-30·54;9·46) | -39·14 (-52·76;-26·26) | -27·13 (-40·59;-11·65) |
| **Russian Federation** | **2010** | 36·41 (28·28;46·41) | 315·55 (245·05;402·25) | 66·73 (61·38;72·37) | 4494·90 (4030·50; 4940·65) | 23·83 (18·67;30·02) | 206·50 (161·83;260·21) | 0·0496 (0·0446;0·0532) | 3·65 (3·23;3·99) |
| | **2019** | 21·13 (15·11;27·86) | 183·11 (130·96;241·47) | 48·10 (41·10;55·18) | 3010·60 (2523·43; 3515·50) | 13·54 (9·78;18·01) | 117·31 (84·76;156·12) | 0·0364 (0·0304;0·0419) | 2·57 (2·11;3·05) |
| | **Change (%) 2010-2019** | -41·97 (-54·02;-27·93) | -41·97 (-54·02;-27·93) | -27·92 (-38·59;-17·50) | -33·02 (-42·30;-24·15) | -43·19 (-55·56;-28·08) | -43·19 (-55·56;-28·08) | -26·70 (-37·84;-16·01) | -29·48 (-39·96;-19·08) |

| | | | | | | | | | |
|---|---|---|---|---|---|---|---|---|---|
| **Ukraine** | **2010** | 16·96 (13·00;22·06) | 147·01 (112·70;191·20) | 94·05 (82·05;104·66) | 4585·86 (3983·18; 5140·29) | 37·51 (29·19;48·73) | 325·08 (252·96;422·37) | 0·1800 (0·1548;0·2007) | 9·90 (8·37;11·23) |
| | **2019** | 10·40 (7·37;13·85) | 90·17 (63·87;119·99) | 55·76 (34·84;74·53) | 2751·15 (1775·68; 3616·38) | 23·68 (17·00;31·30) | 205·27 (147·35;271·28) | 0·1153 (0·0726;0·1534) | 6·55 (4·27;8·70) |
| | **Change (%) 2010-2019** | -38·67 (-52·75;-21·71) | -38·67 (-52·75;-21·71) | -40·71 (-64·11;-18·78) | -40·01 (-62·49;-18·11) | -36·86 (-51·11;-18·91) | -36·86 (-51·11;-18·91) | -35·94 (-61·24;-11·96) | -33·87 (-58·66;-10·03) |
| **Cirrhosis and other chronic liver diseases due to hepatitis** | | | | | | | | | |
| **Belarus** | **2010** | 66·30 (56·89;76·18) | 0·36 (0·22;0·55) | 439·64 (305·16;612·01) | 15835·13 (10911·64; 22123·71) | 614·22 (523·69;707·56) | 3·46 (2·10;5·34) | 3·3415 (2·3472; 4·6089) | 125·02 (88·39;173·80) |
| | **2019** | 52·63 (44·89;59·74) | 0·29 (0·17;0·46) | 265·21 (165·50;402·07) | 9091·06 (5682·19; 13732·16) | 457·60 (391·33;518·87) | 2·78 (1·66;4·43) | 1·8844 (1·1926; 2·8389) | 68·90 (43·57;103·66) |
| | **Change (%) 2010-2019** | -20·63 (-27·69;-13·04) | -20·15 (-30·50;-10·27) | -39·67 (-54·25;-20·25) | -42·59 (-56·17;-23·21) | -25·50 (-32·32;-18·13) | -19·69 (-29·86;-9·81) | -43·61 (-57·27;-25·14) | -44·89 (-58·35;-26·65) |
| **Estonia** | **2010** | 8·14 (6·96;9·30) | 0·05 (0·03;0·08) | 54·18 (38·54; 74·31) | 1837·31 (1295·17; 2511·36) | 569·63 (486·52;655·38) | 3·96 (2·41;6·07) | 2·8443 (2·0323; 3·8599) | 103·19 (72·60;140·87) |
| | **2019** | 6·93 (5·89;8·05) | 0·03 (0·02;0·05) | 33·17 (21·29; 50·51) | 1110·30 (697·68; 1729·20) | 458·24 (389·17;532·92) | 2·30 (1·38;3·63) | 1·7146 (1·0827; 2·6732) | 62·54 (38·71;97·10) |
| | **Change (%) 2010-2019)** | -14·86 (-23·25;-4·75) | -42·56 (-50·12;-34·87) | -38·78 (-53·90;-21·50) | -39·57 (-54·69;-22·07) | -19·56 (-27·60;-10·10) | -41·93 (-49·42;-34·20) | -39·72 (-54·80;-21·86) | -39·39 (-54·49;-21·65) |
| **Latvia** | **2010** | 13·80 (11·86;15·58) | 0·05 (0·03;0·08) | 62·03 (43·55; 86·06) | 2141·16 (1487·47; 3000·91) | 597·23 (515·44;677·40) | 2·48 (1·49;3·96) | 2·0608 (1·4572; 2·8622) | 76·62 (52·96;106·94) |
| | **2019** | 10·27 (8·76;11·71) | 0·04 (0·02;0·06) | 47·73 (30·87; 70·13) | 1587·46 (1023·62; 2389·27) | 457·33 (392·49;515·97) | 2·15 (1·26;3·39) | 1·6464 (1·0662; 2·4589) | 60·82 (39·08;91·37) |
| | **Change (%) 2010-2019** | -25·61 (-32·71;-17·80) | -23·19 (-32·68;-11·86) | -23·05 (-38·33;-4·61) | -25·86 (-40·47;-7·94) | -23·42 (-30·83;-15·47) | -13·24 (-23·66;-0·98) | -20·11 (-36·15;-0·52) | -20·63 (-36·66;-1·46) |
| **Lithuania** | **2010** | 19·37 (15·91;22·43) | 0·11 (0·06;0·17) | 152·65 (105·87;212·67) | 5534·57 (3812·31; 7838·90) | 567·07 (460·73;662·74) | 3·62 (2·13;5·80) | 3·6036 (2·4877; 5·0355) | 138·30 (94·80;195·79) |
| | **2019** | 14·35 (12·15;16·78) | 0·07 (0·04;0·12) | 100·85 (66·83;150·62) | 3502·56 (2289·54; 5315·83) | 431·42 (362·08;501·93) | 2·94 (1·73;4·77) | 2·4682 (1·6178; 3·7153) | 94·41 (61·00;142·11) |
| | **Change (%) 2010-2019** | -25·92 (-33·12;-18·02) | -30·38 (-39·07;-19·84) | -33·93 (-47·74;-17·60) | -36·71 (-49·68;-20·72) | -23·92 (-31·20;-16·32) | -18·98 (-29·01;-6·29) | -31·51 (-45·69;-15·11) | -31·73 (-45·74;-15·47) |
| **Republic of Moldova** | **2010** | 49·27 (41·93;55·77) | 0·55 (0·35;0·84) | 458·87 (316·40;635·67) | 14757·24 (10157·22; 20661·33) | 1115·05 (946·23;1269·93) | 11·92 (7·46;17·98) | 8·9194 (6·2355;12·3405) | 290·26 (200·31;403·52) |
| | **2019** | 42·61 (36·45;48·28) | 0·46 (0·29;0·70) | 349·94 (238·66;498·54) | 10837·69 (7399·62; 15329·23) | 970·36 (827·66;1093·66) | 10·32 (6·46;15·43) | 6·3362 (4·3693; 8·8518) | 204·00 (141·01;289·17) |
| | **Change (%) 2010-2019** | -13·51 (-20·43;-5·97) | -16·55 (-26·08;-4·50) | -23·74 (-35·27;-10·91) | -26·56 (-38·16;-13·54) | -12·98 (-19·79;-5·34) | -13·42 (-22·30;-1·85) | -28·96 (-39·40;-17·01) | -29·72 (-40·44;-17·14) |
| **Russian Federation** | **2010** | 1230·04 (1096·62;1366·35) | 7·37 (4·54;10·77) | 10619·68 (8978·39;12491·81) | 401867·55 (336904·59; 475680·53) | 782·51 (693·98;873·12) | 4·67 (2·87;6·90) | 5·5168 (4·6831; 6·4397) | 216·70 (181·07;255·74) |
| | **2019** | 886·27 (790·29;982·53) | 4·61 (2·72;7·08) | 5546·64 (4355·71;6944·50) | 202874·32 (156982·36; 254707·80) | 532·93 (473·03;592·58) | 2·84 (1·70;4·32) | 2·7255 (2·1299; 3·4049) | 105·28 (81·17;132·50) |
| | **Change (%) 2010-2019** | -27·95 (-29·66;-25·83) | -37·42 (-45·24;-30·95) | -47·77 (-55·65;-39·02) | -49·52 (-57·28;-41·05) | -31·89 (-33·55;-29·89) | -39·29 (-44·07;-34·86) | -50·60 (-58·09;-42·41) | -51·42 (-58·94;-43·29) |

| | | | | | | | | | |
|---|---|---|---|---|---|---|---|---|---|
| **Ukraine** | **2010** | 762·81 (674·62;846·44) | 2·97 (1·71;4·58) | 4466·51 (3770·79;5278·47) | 171019·71 (143468·14; 203300·19) | 1579·27 (1393·76;1760·58) | 6·13 (3·57;9·48) | 7·2001 (6·1142; 8·4935) | 288·75 (241·91;342·86) |
| | **2019** | 572·97 (503·64;646·57) | 1·91 (0·96;3·17) | 3292·01 (2511·54;4213·22) | 128396·77 (97111·66; 165526·07) | 1177·03 (1033·27;1324·80) | 4·04 (2·12;6·53) | 5·3961 (4·1002; 6·9334) | 221·93 (167·28;285·89) |
| | **Change (%) 2010-2019** | -24·89 (-30·08;-19·21) | -35·57 (-48·59;-24·50) | -26·30 (-40·27;-10·52) | -24·92 (-39·07;-8·79) | -25·47 (-30·58;-19·87) | -34·05 (-45·28;-24·28) | -25·06 (-38·82;-8·91) | -23·14 (-37·36;-6·64) |
| **Liver cancer due to hepatitis** | | | | | | | | | |
| **Belarus** | **2010** | 0·11 (0·08;0·15) | 0·09 (0·07;0·13) | 88·13 (63·59;121·71) | 2623·42 (1927·07; 3595·60) | 0·80 (0·59;1·08) | 0·67 (0·50;0·92) | 0·6357 (0·4626;0·8761) | 19·86 (14·90;26·81) |
| | **2019** | 0·09 (0·06;0·15) | 0·08 (0·05;0·12) | 72·00 (43·56;112·94) | 2037·83 (1235·61; 3213·13) | 0·65 (0·40;1·00) | 0·52 (0·32;0·80) | 0·4743 (0·2916;0·7377) | 14·35 (8·70;22·20) |
| | **Change (%) 2010-2019** | -11·76 (-42·04;27·31) | -16·39 (-44·21;19·05) | -18·31 (-44·99;15·66) | -22·32 (-49·07;11·79) | -17·93 (-45·88;18·34) | -23·10 (-48·57;10·72) | -25·38 (-49·65;6·45) | -27·75 (-52·15;5·25) |
| **Estonia** | **2010** | 0·02 (0·01;0·02) | 0·01 (0·01;0·02) | 14·03 (9·88; 19·50) | 360·56 (262·26; 491·58) | 0·82 (0·61;1·11) | 0·67 (0·49;0·92) | 0·6361 (0·4612;0·8697) | 18·00 (13·49;24·40) |
| | **2019** | 0·02 (0·01;0·03) | 0·02 (0·01;0·02) | 15·86 (10·00; 23·45) | 394·52 (247·63; 593·12) | 0·95 (0·61;1·39) | 0·73 (0·47;1·08) | 0·6728 (0·4302;0·9933) | 18·95 (12·14;28·17) |
| | **Change (%) 2010-2019** | 21·23 (-10·49;57·42) | 15·52 (-13·69;47·90) | 13·00 (-15·27;44·19) | 9·42 (-19·01;41·91) | 15·66 (-14·27;50·06) | 9·10 (-18·70;40·82) | 5·77 (-20·87;35·94) | 5·24 (-22·32;37·05) |
| **Latvia** | **2010** | 0·02 (0·02;0·03) | 0·02 (0·01;0·03) | 20·13(14·14; 28·27) | 537·46(394·87; 724·22) | 0·69 (0·51;0·92) | 0·60 (0·44;0·80) | 0·5776(0·4196;0·7881) | 17·10 (12·79;22·60) |
| | **2019** | 0·02 (0·01;0·03) | 0·02 (0·01;0·03) | 18·50(12·34; 27·16) | 477·38(323·30; 693·15) | 0·68 (0·46;0·97) | 0·56 (0·38;0·81) | 0·5316(0·3593;0·7670) | 15·80 (10·88;22·54) |
| | **Change (%) 2010-2019** | -5·53 (-26·54;19·76) | -7·56 (-27·55;16·25) | -8·09 (-27·45;14·94) | -11·18 (-30·99;13·37) | -2·37 (-24·31;25·20) | -6·24 (-26·45;19·66) | -7·96 (-27·35;16·82) | -7·62 (-28·29;19·22) |
| **Lithuania** | **2010** | 0·04 (0·03;0·05) | 0·03 (0·02;0·04) | 27·54 (19·29; 38·06) | 756·33 (540·62; 1020·77) | 0·81 (0·59;1·07) | 0·62 (0·45;0·83) | 0·5624 (0·3998;0·7561) | 16·95 (12·41;22·50) |
| | **2019** | 0·04 (0·03;0·06) | 0·03 (0·02;0·05) | 30·41 (20·01; 44·94) | 806·45 (532·28; 1190·03) | 0·95 (0·63;1·38) | 0·69 (0·46;1·01) | 0·6064 (0·4030;0·8987) | 18·16 (12·09;26·43) |
| | **Change (%) 2010-2019** | 16·94 (-11·81;47·63) | 12·40 (-14·39;41·56) | 10·43 (-15·37;38·33) | 6·63 (-19·07;36·37) | 17·32 (-11·50;49·11) | 10·89 (-15·45;40·02) | 7·82 (-17·22;36·44) | 7·10 (-18·64;37·90) |
| **Republic of Moldova** | **2010** | 0·03 (0·02;0·04) | 0·03 (0·02;0·04) | 27·34 (19·52; 38·11) | 786·67 (573·17; 1084·81) | 0·59 (0·43;0·81) | 0·54 (0·39;0·74) | 0·5323 (0·3823;0·7346) | 15·48 (11·37;21·23) |
| | **2019** | 0·03 (0·02;0·04) | 0·02 (0·02;0·03) | 23·91 (16·22; 34·87) | 652·44 (450·29; 936·20) | 0·49 (0·34;0·68) | 0·43 (0·30;0·61) | 0·4241 (0·2914;0·6113) | 12·12 (8·55;16·94) |
| | **Change (%) 2010-2019** | -11·88 (-29·49;10·06) | -12·78 (-30·22;8·13) | -12·55 (-29·90;8·32) | -17·06 (-33·63;4·10) | -17·68 (-33·40;3·54) | -19·77 (-35·00;-0·28) | -20·33 (-35·37;-1·17) | -21·73 (-37·20;-1·47) |
| **Russian Federation** | **2010** | 1·59 (1·37;1·84) | 1·43 (1·23;1·66) | 1404·54 (1207·72;1630·11) | 42588·10 (36659·10; 49387·15) | 0·80 (0·69;0·92) | 0·71 (0·61;0·82) | 0·6883 (0·5940;0·7965) | 21·61 (18·68;24·87) |
| | **2019** | 1·73 (1·33;2·24) | 1·53 (1·19;1·99) | 1503·27 (1149·52;1934·72) | 44069·89 (34085·81; 57445·55) | 0·82 (0·63;1·05) | 0·70 (0·55;0·91) | 0·6785 (0·5258;0·8765) | 21·19 (16·48;27·44) |
| | **Change (%) 2010-2019** | 8·52 (-12·73;37·16) | 6·84 (-13·92;34·20) | 7·03 (-12·94;36·43) | 3·48 (-16·94;35·25) | 2·02 (-18·28;29·43) | -0·94 (-20·39;24·93) | -1·41 (-19·53;26·55) | -1·94 (-21·01;27·79) |

| | | | | | | | | | |
|---|---|---|---|---|---|---|---|---|---|
| **Ukraine** | **2010** | 0·56 (0·48;0·65) | 0·49 (0·42;0·57) | 472·40 (403·29;554·71) | 13803·85 (11857·95; 15991·75) | 0·84 (0·73;0·97) | 0·72 (0·62;0·83) | 0·6821 (0·5853;0·7938) | 21·12 (18·29;24·38) |
| | **2019** | 0·53 (0·43;0·67) | 0·47 (0·37;0·58) | 451·32 (355·43;565·63) | 12887·24 (10054·95; 16272·53) | 0·82 (0·65;1·03) | 0·68 (0·55;0·86) | 0·6436 (0·5060;0·8075) | 20·15 (15·77;25·16) |
| | **Change (%) 2010-2019** | -4·95 (-22·75;16·88) | -4·66 (-21·78;16·56) | -4·46 (-21·77;15·47) | -6·64 (-24·50;14·18) | -3·15 (-21·39;20·34) | -4·73 (-22·04;17·50) | -5·65 (-23·24;13·53) | -4·61 (-23·46;17·61) |

UI= Uncertainty Interval

**Supplement Table E·** Prevalence, incidence, Mortality and DALYs attributable to HBV in 2010 and 2019, by Western Europe countries·

| | | **N in thousands (95%UI)** | | **N (95%UI)** | | **Age- standardized rate x 100,000 (95%UI)** | | | |
|---|---|---|---|---|---|---|---|---|---|
| | **Year** | **Prevalence** | **Incidence** | **Mortality** | **DALYs** | **Prevalence** | **Incidence** | **Mortality** | **DALYs** |
| **Acute hepatitis** | | | | | | | | | |
| **Andorra** | **2010** | 0·02 (0·02;0·03) | 0·20 (0·13;0·27) | 0·61 (0·45;0·82) | 18·84 (14·54; 24·24) | 24·10 (16·80;31·90) | 208·89 (145·56;276·47) | 0·5449 (0·4150;0·7253) | 18·88 (14·68;23·85) |
| | **2019** | 0·02 (0·01;0·02) | 0·15 (0·10;0·21) | 0·48 (0·35;0·66) | 13·60 (9·78; 18·59) | 17·30 (11·91;23·72) | 149·90 (103·24;205·58) | 0·3778 (0·2705;0·5164) | 12·35 (8·98;16·72) |
| | **Change (%) 2010-2019** | -23·06 (-41·67;-0·99) | -23·06 (-41·67;-0·99) | -20·56 (-39·98;6·48) | -27·84 (-45·20;-6·11) | -28·24 (-44·68;-9·06) | -28·24 (-44·68;-9·06) | -30·67 (-47·55;-8·94) | -34·57 (-49·36;-15·61) |
| **Austria** | **2010** | 1·76 (1·18;2·42) | 15·28 (10·22;20·94) | 1·50 (1·20;2·34) | 84·69 (66·54; 111·60) | 21·70 (15·63;29·72) | 188·07 (135·50;257·53) | 0·0130(0·0108;0·0186) | 0·86 (0·68;1·14) |
| | **2019** | 1·53 (1·00;2·11) | 13·30 (8·64;18·32) | 1·72 (1·42;2·22) | 85·85 (69·48; 107·67) | 16·63 (11·41;22·71) | 144·13 (98·87;196·81) | 0·0130(0·0111;0·0156) | 0·79 (0·64;0·99) |
| | **Change (%) 2010-2019** | -12·98 (-34·91;14·36) | -12·98 (-34·91;14·36) | 15·11 (-8·86;40·28) | 1·37 (-14·40;18·36) | -23·36 (-41·88;0·68) | -23·36 (-41·88;0·68) | 0·28 (-19·77;20·10) | -7·97 (-22·16;7·82) |
| **Belgium** | **2010** | 2·18 (1·72;2·74) | 18·88 (14·91;23·77) | 1·82 (1·34;2·86) | 96·92 (71·99; 132·63) | 19·42 (15·61;24·23) | 168·34 (135·32;210·00) | 0·0114 (0·0086;0·0174) | 0·74 (0·55;1·01) |
| | **2019** | 1·94 (1·44;2·47) | 16·86 (12·47;21·38) | 1·96 (1·37;3·11) | 96·37 (70·25; 130·21) | 15·53 (11·69;19·68) | 134·55 (101·28;170·55) | 0·0110 (0·0079;0·0164) | 0·68 (0·51;0·90) |
| | **Change (%) 2010-2019** | -10·73 (-29·18;11·48) | -10·73 (-29·18;11·48) | 7·60 (-13·43;29·85) | -0·57 (-14·70;15·18) | -20·07 (-36·62;-0·58) | -20·07 (-36·62;-0·58) | -3·69 (-22·02;16·09) | -7·44 (-20·76;7·74) |
| **Cyprus** | **2010** | 0·36 (0·30;0·44) | 3·15 (2·57;3·79) | 0·50 (0·44;0·58) | 24·13 (20·30; 29·02) | 27·72 (22·66;33·15) | 240·22 (196·40;287·30) | 0·0378 (0·0332;0·0438) | 1·84 (1·55;2·21) |
| | **2019** | 0·35 (0·29;0·42) | 3·06 (2·52;3·66) | 0·49 (0·41;0·58) | 23·26 (19·14; 28·07) | 21·04 (17·41;25·05) | 182·33 (150·89;217·11) | 0·0285 (0·0242;0·0332) | 1·42 (1·18;1·72) |
| | **Change (%) 2010-2019** | -3·06 (-21·49;20·86) | -3·06 (-21·49;20·86) | -1·99 (-18·49;17·19) | -3·62 (-17·90;13·06) | -24·10 (-38·51;-6·14) | -24·10 (-38·51;-6·14) | -24·47 (-36·88;-9·95) | -22·72 (-34·37;-9·16) |
| **Denmark** | **2010** | 1·34 (1·02;1·77) | 11·65 (8·82;15·32) | 1·56 (1·37;1·88) | 74·45 (62·50; 90·17) | 27·65 (21·50;35·02) | 239·62 (186·29;303·50) | 0·0206 (0·0180;0·0243) | 1·18 (0·98;1·44) |
| | **2019** | 1·37 (1·01;1·77) | 11·85 (8·78;15·32) | 1·53 (1·31;1·78) | 70·86 (58·63; 87·34) | 27·41 (21·22;35·13) | 237·57 (183·94;304·45) | 0·0183 (0·0157;0·0209) | 1·09 (0·89;1·35) |
| | **Change (%) 2010-2019** | 1·70 (-18·38;27·26) | 1·70 (-18·38;27·26) | -1·90 (-14·69;12·32) | -4·83 (-16·63;7·95) | -0·86 (-18·75;23·82) | -0·86 (-18·75;23·82) | -10·86 (-23·14;2·75) | -8·20 (-20·26;4·66) |
| **Finland** | **2010** | 1·28 (0·96;1·68) | 11·11 (8·34;14·52) | 1·17 (1·02;1·43) | 60·04 (49·28; 74·91) | 27·84 (21·76;35·24) | 241·31 (188·63;305·39) | 0·0153 (0·0135;0·0178) | 0·99 (0·80;1·24) |
| | **2019** | 1·26 (0·94;1·64) | 10·96 (8·18;14·24) | 1·11 (0·93;1·35) | 55·90 (45·39; 70·06) | 27·58 (21·56;35·58) | 239·06 (186·87;308·32) | 0·0136 (0·0116;0·0156) | 0·91 (0·72;1·17) |
| | **Change (%) 2010-2019** | -1·40 (-21·23;23·05) | -1·40 (-21·23;23·05) | -5·50 (-17·12;7·92) | -6·91 (-17·63;5·20) | -0·93 (-20·04;22·23) | -0·93 (-20·04;22·23) | -11·31 (-22·70;1·62) | -7·71 (-19·04;5·08) |
| **France** | **2010** | 34·61 | 299·99 | 10·20 | 972·12 | 53·74 | 465·71 | 0·0115 | 1·33 |

| | | | | | | | | | |
|---|---|---|---|---|---|---|---|---|---|
| | | (28·32;42·04) | (245·41;364·34) | (8·37;13·86) | (689·14; 1361·25) | (44·66;64·16) | (387·02;556·03) | (0·0095;0·0153) | (0·94;1·88) |
| | **2019** | 30·93 (23·68;38·13) | 268·04 (205·18;330·50) | 10·28 (8·08;13·55) | 928·28 (654·87; 1294·69) | 45·02 (35·86;54·26) | 390·21 (310·76;470·28) | 0·0106 (0·0084;0·0137) | 1·21 (0·86;1·70) |
| | **Change (%) 2010-2019** | -10·65 (-27·02;11·36) | -10·65 (-27·02;11·36) | 0·71 (-12·87;19·86) | -4·51 (-21·24;16·47) | -16·21 (-31·66;2·93) | -16·21 (-31·66;2·93) | -7·52 (-20·01;9·81) | -8·95 (-25·45;12·42) |
| **Germany** | **2010** | 11·31 (8·73;14·30) | 98·04 (75·64;123·90) | 16·97 (13·92;22·43) | 703·42 (558·23; 890·43) | 13·25 (10·69;16·67) | 114·81 (92·68;144·49) | 0·0127 (0·0104;0·0167) | 0·67 (0·53;0·86) |
| | **2019** | 10·63 (8·36;13·49) | 92·14 (72·41;116·88) | 17·91 (13·88;24·63) | 707·97 (556·78; 918·80) | 11·42 (8·96;14·42) | 99·01 (77·68;125·02) | 0·0123 (0·0096;0·0165) | 0·65 (0·51;0·84) |
| | **Change (%) 2010-2019** | -6·02 (-25·00;17·65) | -6·02 (-25·00;17·65) | 5·53 (-12·30;23·06) | 0·65 (-11·92;14·45) | -13·76 (-31·60;7·23) | -13·76 (-31·60;7·23) | -2·96 (-18·73;12·57) | -3·96 (-16·00;8·82) |
| **Greece** | **2010** | 7·04 (5·45;9·02) | 61·01 (47·26;78·21) | 80·90 (61·35;95·67) | 1740·45 (1428·00; 1983·82) | 59·47 (46·78;74·42) | 515·44 (405·39;644·96) | 0·3771 (0·2981;0·4349) | 10·04 (8·56;11·23) |
| | **2019** | 5·74 (4·36;7·15) | 49·71 (37·83;61·93) | 32·78 (25·68;38·87) | 776·10 (650·78; 886·62) | 51·80 (40·88;64·06) | 448·93 (354·30;555·22) | 0·1448 (0·1195;0·1667) | 4·73 (4·09;5·43) |
| | **Change (%) 2010-2019** | -18·53 (-36·01;3·44) | -18·53 (-36·01;3·44) | -59·48 (-65·35;-52·38) | -55·41 (-61·25;-48·98) | -12·90 (-31·31;10·62) | -12·90 (-31·31;10·62) | -61·61 (-66·79;-55·35) | -52·88 (-59·02;-46·04) |
| **Iceland** | **2010** | 0·06 (0·04;0·07) | 0·50 (0·37;0·65) | 0·05 (0·04;0·06) | 2·50 (2·00;3·14) | 19·34 (14·76;24·78) | 167·60 (127·92;214·77) | 0·0127 (0·0107;0·0156) | 0·70 (0·56;0·89) |
| | **2019** | 0·06 (0·04;0·08) | 0·51 (0·37;0·67) | 0·06 (0·05;0·07) | 2·74 (2·17;3·41) | 18·81 (13·95;24·27) | 163·02 (120·87;210·32) | 0·0126 (0·0103;0·0151) | 0·70 (0·55;0·88) |
| | **Change (%) 2010-2019** | 1·78 (-16·51;26·08) | 1·78 (-16·51;26·08) | 16·21 (-1·95;38·27) | 9·75 (-4·34;26·16) | -2·73 (-19·68;18·74) | -2·73 (-19·68;18·74) | -1·12 (-16·50;17·34) | -0·65 (-13·77;14·04) |
| **Ireland** | **2010** | 0·89 (0·61;1·18) | 7·71 (5·28;10·24) | 0·84 (0·64;0·99) | 44·07 (34·02; 56·11) | 19·79 (13·98;25·98) | 171·50 (121·17;225·15) | 0·0154 (0·0117;0·0180) | 0·87 (0·67;1·10) |
| | **2019** | 0·76 (0·53;1·03) | 6·61 (4·59;8·97) | 0·81 (0·58;0·98) | 40·91 (31·09; 52·20) | 15·76 (10·87;21·01) | 136·56 (94·21;182·05) | 0·0127 (0·0091;0·0152) | 0·73 (0·56;0·93) |
| | **Change (%) 2010-2019** | -14·34 (-33·49;10·99) | -14·34 (-33·49;10·99) | -2·75 (-19·43;16·46) | -7·16 (-22·88;11·10) | -20·37 (-38·13;2·94) | -20·37 (-38·13;2·94) | -17·40 (-31·08;-0·91) | -16·01 (-29·81;0·14) |
| **Israel** | **2010** | 1·48 (1·07;1·99) | 12·83 (9·25;17·22) | 1·99 (1·64;2·24) | 92·43 (76·71; 110·40) | 19·00 (13·68;25·47) | 164·69 (118·55;220·76) | 0·0240 (0·0199;0·0269) | 1·16 (0·96;1·39) |
| | **2019** | 1·29 (0·86;1·78) | 11·18 (7·42;15·47) | 2·37 (1·90;2·71) | 102·70 (82·73; 124·74) | 13·97 (9·34;19·12) | 121·04 (80·93;165·67) | 0·0225 (0·0182;0·0257) | 1·06 (0·86;1·29) |
| | **Change (%) 2010-2019** | -12·85 (-35·07;14·93) | -12·85 (-35·07;14·93) | 18·92 (4·59;35·13) | 11·11 (-2·47;27·62) | -26·51 (-45·08;-2·80) | -26·51 (-45·08;-2·80) | -6·34 (-17·12;6·18) | -8·68 (-19·72;4·90) |
| **Italy** | **2010** | 11·39 (8·07;15·06) | 98·73 (69·91;130·55) | 33·02 (20·27;52·56) | 1144·36 (793·87; 1714·11) | 16·04 (11·69;21·06) | 139·01 (101·29;182·50) | 0·0332 (0·0204;0·0530) | 1·49 (1·00;2·27) |
| | **2019** | 7·91 (5·05;11·15) | 68·57 (43·80;96·66) | 34·19 (23·32;44·40) | 1047·13(773·72; 1354·67) | 9·95 (6·82;13·49) | 86·26 (59·10;116·88) | 0·0307(0·0214;0·0403) | 1·26 (0·93;1·65) |
| | **Change (%) 2010-2019** | -30·54 (-45·34;-13·31) | -30·54 (-45·34;-13·31) | 3·55 (-17·21;22·85) | -8·50 (-23·99;5·41) | -37·95 (-50·82;-22·70) | -37·95 (-50·82;-22·70) | -7·47 (-25·96;9·83) | -15·19 (-29·96;-1·88) |
| **Luxembourg** | **2010** | 0·12 (0·08;0·16) | 1·02 (0·71;1·39) | 0·06 (0·05;0·08) | 4·07(2·93; 5·59) | 22·79 (16·61;31·10) | 197·48 (143·94;269·56) | 0·0087(0·0070;0·0118) | 0·68 (0·49;0·93) |

| | | | | | | | | | |
|---|---|---|---|---|---|---|---|---|---|
| | **2019** | 0·13 (0·09;0·17) | 1·10 (0·77;1·49) | 0·06 (0·05;0·09) | 4·63(3·29; 6·41) | 18·55 (13·26;24·44) | 160·79 (114·88;211·85) | 0·0073(0·0055;0·0104) | 0·61 (0·43;0·84) |
| | **Change (%) 2010-2019** | 8·43 (-17·17;42·88) | 8·43 (-17·17;42·88) | 5·88 (-12·63;25·82) | 13·77 (-5·09;34·97) | -18·58 (-37·53;6·54) | -18·58 (-37·53;6·54) | -15·86 (-30·42;0·23) | -10·43 (-25·59;6·27) |
| **Malta** | **2010** | 0·06 (0·04;0·09) | 0·56 (0·38;0·78) | 0·08 (0·06;0·11) | 3·69(2·87; 4·87) | 15·21 (10·76;21·43) | 131·84 (93·29;185·77) | 0·0132 (0·0107;0·0179) | 0·73 (0·57;0·97) |
| | **2019** | 0·05 (0·04;0·07) | 0·46 (0·31;0·63) | 0·08 (0·06;0·11) | 3·43(2·66; 4·48) | 11·55 (8·20;15·54) | 100·08 (71·03;134·70) | 0·0115 (0·0088;0·0153) | 0·64 (0·49;0·84) |
| | **Change (%) 2010-2019** | -17·99 (-37·49;10·80) | -17·99 (-37·49;10·80) | -2·25 (-18·45;18·97) | -6·89 (-19·76;10·16) | -24·09 (-40·90;0·68) | -24·09 (-40·90;0·68) | -13·24 (-26·97;3·87) | -12·28 (-24·54;4·19) |
| **Monaco** | **2010** | 0·01 (0·00;0·01) | 0·06 (0·04;0·08) | 0·02 (0·01;0·02) | 0·63 (0·51; 0·78) | 18·00 (12·26;24·18) | 156·01 (106·25;209·60) | 0·0302 (0·0240;0·0377) | 1·44 (1·17;1·79) |
| | **2019** | 0·01 (0·00;0·01) | 0·05 (0·03;0·07) | 0·02 (0·01;0·02) | 0·57 (0·45; 0·74) | 12·00 (7·70;16·79) | 103·98 (66·76;145·48) | 0·0260 (0·0199;0·0328) | 1·20 (0·93;1·56) |
| | **Change (%) 2010-2019** | -18·00 (-38·25;6·01) | -18·00 (-38·25;6·01) | -4·89 (-27·52;25·30) | -9·09 (-27·31;11·63) | -33·35 (-50·98;-11·80) | -33·35 (-50·98;-11·80) | -13·84 (-34·18;9·87) | -16·99 (-33·89;2·71) |
| **Netherlands** | **2010** | 4·11 (3·07;5·46) | 35·66 (26·60;47·30) | 3·25 (2·83;3·80) | 179·5 3(143·66; 225·33) | 26·46 (20·41;33·72) | 229·31 (176·93;292·25) | 0·0142 (0·0125;0·0164) | 0·93 (0·75;1·17) |
| | **2019** | 3·74 (2·57;4·88) | 32·42 (22·23;42·28) | 3·32 (2·85;3·99) | 174·77 (139·79; 223·57) | 22·00 (15·58;28·95) | 190·66 (135·06;250·91) | 0·0132 (0·0115;0·0153) | 0·86 (0·68;1·11) |
| | **Change (%) 2010-2019** | -9·10 (-27·02;13·90) | -9·10 (-27·02;13·90) | 2·21 (-10·23;18·50) | -2·65 (-14·58;11·89) | -16·86 (-33·58;2·99) | -16·86 (-33·58;2·99) | -7·17 (-18·38;8·36) | -7·40 (-18·21;6·57) |
| **Norway** | **2010** | 0·76 (0·56;0·98) | 6·56 (4·82;8·52) | 1·44 (1·14;1·71) | 56·63 (48·04; 68·05) | 15·98 (12·11;20·67) | 138·46 (104·93;179·15) | 0·0210 (0·0172;0·0249) | 1·00 (0·84;1·19) |
| | **2019** | 0·58 (0·38;0·80) | 5·01 (3·26;6·93) | 1·35 (1·09;1·58) | 50·45 (43·22; 59·52) | 10·52 (6·94;14·39) | 91·18 (60·10;124·68) | 0·0171 (0·0144;0·0195) | 0·79 (0·68;0·93) |
| | **Change (%) 2010-2019** | -23·65 (-38·93;-6·49) | -23·65 (-38·93;-6·49) | -6·12 (-13·55;1·11) | -10·92 (-18·94;-3·00) | -34·15 (-47·57;-19·27) | -34·15 (-47·57;-19·27) | -18·57 (-25·60;-11·62) | -20·80 (-28·55;-13·13) |
| **Portugal** | **2010** | 2·67 (1·83;3·67) | 23·12 (15·82;31·80) | 2·73 (2·31;3·52) | 141·85 (110·93; 184·63) | 24·88 (17·29;34·05) | 215·65 (149·86;295·10) | 0·0182 (0·0154;0·0233) | 1·14 (0·89;1·50) |
| | **2019** | 2·01 (1·26;2·87) | 17·44 (10·93;24·92) | 2·51 (2·07;3·08) | 119·36 (93·29; 155·47) | 17·96 (12·04;24·96) | 155·70 (104·37;216·35) | 0·0152 (0·0126;0·0186) | 0·93 (0·72;1·19) |
| | **Change (%) 2010-2019** | -24·56 (-44·92;2·18) | -24·56 (-44·92;2·18) | -7·98 (-24·36;10·02) | -15·86 (-30·44;1·21) | -27·80 (-47·28;-1·36) | -27·80 (-47·28;-1·36) | -16·53 (-30·94;-0·04) | -18·44 (-32·69;-1·13) |
| **San Marino** | **2010** | 0·01 (0·00;0·01) | 0·06 (0·04;0·08) | 0·08 (0·05;0·12) | 2·33 (1·55; 3·39) | 20·47 (14·85;26·88) | 177·43 (128·67;232·96) | 0·1770 (0·1142;0·2640) | 6·08 (4·05;8·86) |
| | **2019** | 0·01 (0·00;0·01) | 0·06 (0·04;0·08) | 0·09 (0·05;0·14) | 2·33 (1·39; 3·69) | 18·63 (13·80;23·64) | 161·48 (119·57;204·84) | 0·1572 (0·0920;0·2510) | 5·36 (3·31;8·52) |
| | **Change (%) 2010-2019** | -1·83 (-22·56;24·27) | -1·83 (-22·56;24·27) | 3·08 (-31·96;53·65) | 0·25 (-31·98;46·08) | -8·99 (-28·35;16·00) | -8·99 (-28·35;16·00) | -11·18 (-41·15;31·38) | -11·80 (-39·67;26·56) |
| **Spain** | **2010** | 12·48 (9·22;16·25) | 108·20 (79·88;140·84) | 6·72 (5·04;9·00) | 472·54 (350·30; 640·25) | 23·79 (18·19;30·67) | 206·20 (157·67;265·77) | 0·0106 (0·0083;0·0141) | 0·87 (0·66;1·16) |
| | **2019** | 9·61 | 83·25 | 7·34 | 437·01 | 17·50 | 151·71 | 0·0106 | 0·78 |

| | | | | | | | | | |
|---|---|---|---|---|---|---|---|---|---|
| | | (6·93;12·66) | (60·03;109·72) | (4·97;9·06) | (319·23; 578·07) | (13·17;22·48) | (114·11;194·81) | (0·0074;0·0130) | (0·58;1·03) |
| | **Change (%) 2010-2019** | -23·06 (-41·80;-1·29) | -23·06 (-41·80;-1·29) | 9·26 (-11·01;30·58) | -7·52 (-23·40;12·21) | -26·43 (-43·24;-6·29) | -26·43 (-43·24;-6·29) | -0·10 (-18·97;20·31) | -9·88 (-25·81;9·35) |
| **Sweden** | **2010** | 1·26 (0·95;1·64) | 10·96 (8·20;14·18) | 2·00 (1·77;2·33) | 81·66 (70·26; 96·45) | 15·73 (12·24;19·75) | 136·34 (106·06;171·18) | 0·0146 (0·0130;0·0166) | 0·75 (0·64;0·89) |
| | **2019** | 1·24 (0·89;1·65) | 10·72 (7·70;14·33) | 2·19 (1·82;2·45) | 85·16(71·61; 101·73) | 13·50 (9·79;17·77) | 117·01 (84·86;154·03) | 0·0142 (0·0119;0·0158) | 0·72 (0·60;0·86) |
| | **Change (%) 2010-2019** | -2·16 (-20·59;19·32) | -2·16 (-20·59;19·32) | 9·37 (-5·31;23·68) | 4·29 (-7·79;15·65) | -14·18 (-29·53;3·76) | -14·18 (-29·53;3·76) | -2·51 (-15·46;9·47) | -4·82 (-15·42;6·07) |
| **Switzerland** | **2010** | 1·85 (1·35;2·50) | 16·06 (11·74;21·69) | 1·23 (0·92;2·16) | 72·51 (53·16; 103·35) | 25·42 (19·39;32·51) | 220·34 (168·05;281·76) | 0·0115 (0·0089;0·0184) | 0·86 (0·65;1·15) |
| | **2019** | 1·91 (1·40;2·47) | 16·55 (12·09;21·43) | 1·40 (1·03;2·28) | 78·00 (57·72; 107·91) | 23·50 (17·85;30·00) | 203·69 (154·67;260·02) | 0·0111 (0·0085;0·0168) | 0·82 (0·62;1·07) |
| | **Change (%) 2010-2019** | 3·04 (-18·95;31·50) | 3·04 (-18·95;31·50) | 13·99 (-3·82;40·63) | 7·58 (-8·45;27·07) | -7·56 (-25·11;15·26) | -7·56 (-25·11;15·26) | -3·31 (-17·61;17·80) | -4·67 (-18·70;13·32) |
| **United Kingdom** | **2010** | 12·54 (9·87;15·67) | 108·64 (85·57;135·81) | 14·50 (10·58;15·98) | 691·92 (532·68; 846·31) | 21·88 (17·75;26·79) | 189·63 (153·81;232·18) | 0·0172 (0·0124;0·0190) | 1·00 (0·74;1·23) |
| | **2019** | 12·85 (9·83;16·13) | 111·33 (85·21;139·82) | 13·71(10·70;15·25) | 661·77 (528·12; 807·10) | 20·58 (15·86;25·80) | 178·34 (137·43;223·57) | 0·0146 (0·0112;0·0163) | 0·88 (0·69;1·08) |
| | **Change (%) 2010-2019** | 2·47 (-12·90;20·19) | 2·47 (-12·90;20·19) | -5·46 (-13·23;4·62) | -4·36 (-12·88;6·13) | -5·95 (-19·72;9·19) | -5·95 (-19·72;9·19) | -14·91 (-22·15;-5·15) | -11·99 (-20·38;-1·14) |
| **Cirrhosis and other chronic liver diseases due to hepatitis** | | | | | | | | | |
| **Andorra** | **2010** | 0·86 (0·71;0·99) | 0·00 (0·00;0·00) A | 0·76 (0·50; 1·12) | 22·80 (14·74; 33·87) | 842·88 (696·86;980·75) | 1·37 (0·85;2·12) | 0·6192 (0·4058; 0·9238) | 18·64 (12·05;27·82) |
| | **2019** | 0·76 (0·62;0·89) | 0·00 (0·00;0·00) B | 0·77 (0·48; 1·18) | 22·08 (13·37; 34·93) | 672·23 (550·75;791·74) | 1·18 (0·73;1·77) | 0·5515 (0·3456; 0·8483) | 16·47 (10·04;25·96) |
| | **Change (%) 2010-2019** | -11·47 (-19·23;-3·85) | -17·91 (-26·90;-8·47) | 1·56 (-18·93;29·51) | -3·16 (-23·48;25·09) | -20·25 (-27·50;-13·36) | -14·16 (-23·95;-4·94) | -10·94 (-29·17;13·82) | -11·64 (-30·40;13·62) |
| **Austria** | **2010** | 69·88 (57·97;81·07) | 0·32 (0·20;0·49) | 166·81 (113·67;228·41) | 4705·77 (3134·75; 6590·81) | 742·05 (610·69;865·37) | 3·01 (1·87;4·58) | 1·2266 (0·8366; 1·7058) | 37·38 (24·83;52·47) |
| | **2019** | 67·53 (54·46;80·82) | 0·18 (0·11;0·28) | 115·60 (78·67;161·66) | 3084·45 (2077·41; 4412·89) | 621·78 (508·86;740·82) | 1·79 (1·10;2·75) | 0·7412 (0·5080; 1·0428) | 22·06 (14·93;31·49) |
| | **Change (%) 2010-2019** | -3·37 (-13·15;6·36) | -42·79 (-48·34;-36·44) | -30·70 (-36·57;-23·67) | -34·45 (-39·87;-28·17) | -16·21 (-24·78;-7·38) | -40·58 (-46·43;-34·29) | -39·57 (-44·32;-33·81) | -40·99 (-45·76;-35·59) |
| **Belgium** | **2010** | 67·09 (60·85;74·12) | 0·21 (0·12;0·32) | 121·52 (84·14;166·57) | 3192·31 (2208·69; 4521·01) | 539·03 (488·28;600·42) | 1·61 (0·97;2·48) | 0·6773 (0·4778; 0·9377) | 19·73 (13·62;28·07) |
| | **2019** | 61·65 (55·84;68·32) | 0·13 (0·08;0·20) | 90·22 (61·66;126·68) | 2252·12 (1518·17; 3240·67) | 437·95 (397·89;486·56) | 1·04 (0·64;1·62) | 0·4455 (0·3070; 0·6308) | 12·82 (8·55;18·44) |
| | **Change (%) 2010-2019** | -8·12 (-13·61;-1·67) | -36·42 (-44·60;-27·84) | -25·75 (-33·66;-17·79) | -29·45 (-36·72;-21·36) | -18·75 (-23·91;-12·86) | -35·23 (-43·54;-27·26) | -34·22 (-40·50;-27·19) | -35·03 (-41·58;-27·63) |
| **Cyprus** | **2010** | 8·89 (8·58;9·17) | 0·02 (0·01;0·02) | 7·26 (4·87; 10·62) | 174·78 (119·76; 247·67) | 669·14 (646·86;689·87) | 1·18 (0·75;1·79) | 0·5579 (0·3841; 0·8083) | 12·73 (8·76;18·05) |
| | **2019** | 9·16 | 0·02 | 8·31 | 194·67 | 531·92 | 1·07 | 0·4637 | 10·76 |

| | | | | | | | | | |
|---|---|---|---|---|---|---|---|---|---|
| | | (8·84;9·46) | (0·01;0·03) | (5·55; 12·32) | (135·65; 280·65) | (514·12;549·01) | (0·67;1·63) | (0·3182; 0·6733) | (7·53;15·30) |
| | **Change (%) 2010-2019** | 3·05<br>(0·64;5·45) | 11·01<br>(-6·42;28·90) | 14·43<br>(-3·54;32·90) | 11·38<br>(-5·90;31·39) | -20·51<br>(-22·28;-18·70) | -9·12<br>(-22·29;4·06) | -16·87<br>(-28·64;-3·46) | -15·43<br>(-28·34;-0·49) |
| **Denmark** | **2010** | 49·27<br>(40·17;58·63) | 0·19<br>(0·11;0·28) | 122·86<br>(84·93;173·10) | 3601·03<br>(2448·03; 5207·24) | 859·63<br>(687·62;1030·26) | 3·01<br>(1·84;4·56) | 1·4261<br>(0·9844; 2·0261) | 44·67<br>(30·48;63·23) |
| | **2019** | 51·44<br>(42·81;60·88) | 0·15<br>(0·09;0·23) | 107·73<br>(72·31;151·92) | 2961·61<br>(1987·73; 4151·95) | 857·05<br>(709·87;1027·72) | 2·54<br>(1·59;3·78) | 1·1157<br>(0·7547; 1·5470) | 34·14<br>(23·05;47·97) |
| | **Change (%) 2010-2019** | 4·41<br>(-6·69;16·53) | -18·65<br>(-30·34;-7·15) | -12·32<br>(-22·10;-0·88) | -17·76<br>(-26·41;-7·00) | -0·30<br>(-11·11;10·86) | -15·51<br>(-26·64;-4·73) | -21·76<br>(-29·96;-12·36) | -23·56<br>(-31·42;-14·39) |
| **Finland** | **2010** | 48·57<br>(40·37;57·17) | 0·16<br>(0·09;0·24) | 153·78<br>(103·37;216·77) | 4894·22<br>(3268·06; 7057·25) | 875·55<br>(721·16;1039·81) | 2·92<br>(1·76;4·52) | 1·8301<br>(1·2500; 2·6013) | 62·62<br>(42·19;89·48) |
| | **2019** | 48·96<br>(41·46;57·10) | 0·14<br>(0·09;0·22) | 132·60(<br>90·55;189·92) | 3946·22<br>(2695·09; 5567·54) | 858·90<br>(715·95;1006·55) | 2·62<br>(1·62;4·04) | 1·4887<br>(1·0211; 2·1018) | 49·89<br>(33·98;69·71) |
| | **Change (%) 2010-2019** | 0·81<br>(-10·46;14·11) | -9·97<br>(-20·08;1·78) | -13·77<br>(-23·84;-2·20) | -19·37<br>(-28·61;-8·89) | -1·90<br>(-13·27;11·22) | -10·49<br>(-20·21;0·81) | -18·66<br>(-27·03;-9·46) | -20·33<br>(-28·53;-11·11) |
| **France** | **2010** | 971·54<br>(926·18;1012·23) | 1·71<br>(1·03;2·59) | 1068·51<br>(738·03;1472·50) | 29625·61<br>(19896·30; 41580·31) | 1349·27<br>(1293·17;1409·77) | 2·40<br>(1·48;3·65) | 1·0663<br>(0·7401; 1·4776) | 32·72<br>(22·21;45·87) |
| | **2019** | 912·93<br>(861·31;951·23) | 1·20<br>(0·74;1·85) | 842·64<br>(588·01;1176·17) | 21582·27<br>(14740·30; 30766·94) | 1150·22<br>(1100·79;1198·76) | 1·73<br>(1·06;2·66) | 0·7322(0·5034;<br>1·0236) | 21·91<br>(14·96;31·06) |
| | **Change (%) 2010-2019** | -6·03<br>(-9·17;-2·86) | -29·70<br>(-37·73;-20·92) | -21·14<br>(-29·33;-11·00) | -27·15<br>(-34·74;-18·20) | -14·75<br>(-17·31;-11·92) | -28·04<br>(-36·18;-19·49) | -31·33<br>(-37·81;-23·89) | -33·02<br>(-39·60;-25·24) |
| **Germany** | **2010** | 319·30<br>(274·47;362·75) | 2·86<br>(1·69;4·38) | 1640·58<br>(1141·12;2280·83) | 45136·63<br>(31199·99; 63341·13) | 328·51<br>(279·45;377·65) | 2·86<br>(1·73;4·45) | 1·1320<br>(0·7858; 1·5763) | 34·62<br>(23·56;48·98) |
| | **2019** | 304·22<br>(266·94;342·42) | 1·61<br>(1·00;2·49) | 1269·66<br>(863·48;1781·11) | 32627·57<br>(21823·21; 47121·50) | 284·79<br>(248·62;323·64) | 1·77<br>(1·07;2·74) | 0·7942<br>(0·5422; 1·1354) | 23·80<br>(16·17;34·28) |
| | **Change (%) 2010-2019** | -4·72<br>(-12·13;3·42) | -43·67<br>(-50·96;-34·78) | -22·61<br>(-30·75;-14·69) | -27·71<br>(-35·41;-19·71) | -13·31<br>(-20·76;-5·59) | -37·94<br>(-45·07;-30·20) | -29·84<br>(-36·52;-23·15) | -31·25<br>(-38·10;-24·29) |
| **Greece** | **2010** | 207·67<br>(188·96;228·40) | 0·20<br>(0·12;0·30) | 87·82<br>(60·07;123·53) | 2180·36<br>(1522·91; 3008·37) | 1530·15<br>(1387·84;1679·96) | 1·49<br>(0·94;2·24) | 0·4595<br>(0·3194; 0·6424) | 13·00<br>(8·97;18·10) |
| | **2019** | 185·96<br>(170·08;203·81) | 0·16<br>(0·10;0·24) | 90·67<br>(63·27;126·69) | 2086·40<br>(1435·96; 2880·76) | 1374·57<br>(1261·09;1505·64) | 1·32<br>(0·82;1·98) | 0·4271<br>(0·2944; 0·5937) | 11·96<br>(8·30;16·54) |
| | **Change (%) 2010-2019** | -10·45<br>(-16·18;-3·76) | -18·45<br>(-27·67;-9·24) | 3·25<br>(-7·31;15·77) | -4·31<br>(-13·68;5·43) | -10·17<br>(-15·83;-3·28) | -11·13<br>(-20·53;-1·98) | -7·06<br>(-15·97;1·95) | -8·00<br>(-17·31;1·03) |
| **Iceland** | **2010** | 2·13<br>(1·72;2·54) | 0·00<br>(0·00;0·01) | 2·04<br>(1·41; 2·88) | 58·61<br>(39·90; 81·32) | 655·64<br>(523·39;787·86) | 1·41<br>(0·90;2·03) | 0·4887<br>(0·3368; 0·6912) | 14·62<br>(10·12;20·25) |
| | **2019** | 2·23<br>(1·83;2·64) | 0·01<br>(0·00;0·01) | 2·31<br>(1·60; 3·25) | 65·55<br>(44·64; 92·50) | 631·94<br>(517·25;753·46) | 1·38<br>(0·87;2·02) | 0·4633<br>(0·3191; 0·6495) | 14·27<br>(9·69;19·95) |
| | **Change (%) 2010-2019** | 4·68<br>(-6·91;16·50) | 1·18<br>(-10·72;13·21) | 13·21<br>(-2·15;31·97) | 11·85<br>(-2·73;29·55) | -3·61<br>(-14·82;7·19) | -2·27<br>(-13·79;9·15) | -5·20<br>(-17·92;10·79) | -2·36<br>(-15·45;12·44) |
| **Ireland** | **2010** | 32·22<br>(26·11;38·33) | 0·10<br>(0·06;0·14) | 43·80<br>(30·98; 59·81) | 1372·66<br>(960·23; 1916·32) | 678·70<br>(545·33;809·15) | 1·80<br>(1·13;2·67) | 0·7763<br>(0·5481; 1·0626) | 24·73<br>(17·29;34·63) |
| | **2019** | 30·49<br>(24·46;36·26) | 0·07<br>(0·04;0·10) | 31·02<br>(21·34; 44·10) | 896·59<br>(607·28; 1268·00) | 573·86<br>(456·18;688·09) | 1·19<br>(0·73;1·74) | 0·4474<br>(0·3089; 0·6317) | 13·67<br>(9·28;19·42) |

| | | | | | | | | | |
|---|---|---|---|---|---|---|---|---|---|
| | **Change (%) 2010-2019** | -5·36 (-15·95;6·74) | -30·30 (-38·75;-21·29) | -29·18 (-37·23;-20·13) | -34·68 (-42·20;-26·53) | -15·45 (-25·86;-4·04) | -34·01 (-41·27;-26·99) | -42·38 (-48·73;-35·60) | -44·73 (-51·00;-38·39) |
| **Israel** | **2010** | 50·14 (40·43;60·00) | 0·08 (0·05;0·12) | 37·91 (26·00; 53·44) | 868·76 (612·87; 1207·06) | 634·43 (512·90;761·13) | 1·05 (0·65;1·59) | 0·4311 (0·2995; 0·5977) | 10·39 (7·32;14·40) |
| | **2019** | 49·05 (39·90;57·51) | 0·09 (0·06;0·14) | 45·42 (30·37; 65·13) | 1003·25 (690·90; 1414·01) | 512·57 (419·08;600·18) | 0·99 (0·63;1·52) | 0·3961 (0·2693; 0·5683) | 9·52 (6·55;13·37) |
| | **Change (%) 2010-2019** | -2·17 (-11·16;8·09) | 13·80 (-0·02;29·04) | 19·84 (8·40;31·85) | 15·48 (4·70;27·30) | -19·21 (-26·46;-10·82) | -4·91 (-16·30;7·84) | -8·10 (-16·32;0·89) | -8·37 (-16·73;0·63) |
| **Italy** | **2010** | 512·69 (454·14;570·37) | 2·85 (2·24;3·57) | 1067·44 (920·59;1233·26) | 24591·23 (21413·09; 27625·51) | 639·74 (565·25;713·30) | 3·70 (2·93;4·60) | 0·9110 (0·7938; 1·0322) | 24·46 (21·45;27·59) |
| | **2019** | 430·18 (375·76;484·73) | 2·44 (1·81;3·21) | 1025·30 (870·69;1197·46) | 22427·46 (19548·46; 25686·63) | 463·96 (408·04;520·20) | 3·20 (2·43;4·15) | 0·7713 (0·6671; 0·8887) | 20·48 (17·97;23·30) |
| | **Change (%) 2010-2019** | -16·09 (-19·43;-12·70) | -14·42 (-22·63;-6·26) | -3·95 (-8·07;0·80) | -8·80 (-12·67;-4·23) | -27·48 (-30·31;-24·60) | -13·46 (-20·81;-7·34) | -15·33 (-18·95;-11·19) | -16·26 (-19·88;-12·03) |
| **Luxembourg** | **2010** | 4·09 (3·31;4·88) | 0·02 (0·01;0·03) | 6·48 (4·42; 9·08) | 182·67 (124·68; 256·54) | 723·35 (585·44;867·76) | 2·54 (1·55;3·89) | 0·8817 (0·6043; 1·2446) | 26·06 (17·63;36·42) |
| | **2019** | 4·66 (3·82;5·44) | 0·01 (0·01;0·02) | 5·71 (3·93; 8·13) | 160·12 (107·89; 227·19) | 615·65 (506·47;724·67) | 1·65 (1·02;2·55) | 0·6015 (0·4116; 0·8580) | 17·98 (12·06;25·40) |
| | **Change (%) 2010-2019** | 13·69 (3·73;24·23) | -22·51 (-31·93;-12·75) | -11·89 (-24·29;2·28) | -12·35 (-23·93;1·74) | -14·89 (-22·15;-7·18) | -35·09 (-42·35;-27·35) | -31·77 (-40·82;-20·93) | -30·99 (-40·32;-19·96) |
| **Malta** | **2010** | 2·90 (2·39;3·50) | 0·00 (0·00;0·01) | 2·77 (1·88; 3·93) | 77·85 (53·38; 110·01) | 606·40 (499·33;736·16) | 1·02 (0·63;1·55) | 0·4221 (0·2900; 0·5928) | 12·86 (8·77;17·97) |
| | **2019** | 2·66 (2·19;3·17) | 0·00 (0·00;0·01) | 2·83 (1·92; 4·16) | 75·62 (51·69; 108·19) | 496·65 (404·68;590·80) | 0·91 (0·57;1·38) | 0·3695 (0·2523; 0·5298) | 11·46 (7·77;16·43) |
| | **Change (%) 2010-2019** | -8·04 (-17·50;2·01) | -8·20 (-17·92;3·28) | 2·14 (-11·06;18·59) | -2·87 (-15·95;12·10) | -18·10 (-27·01;-8·80) | -10·60 (-19·32;-0·04) | -12·46 (-23·29;0·13) | -10·89 (-21·89;2·40) |
| **Monaco** | **2010** | 0·27 (0·22;0·32) | 0·00 (0·00;0·00) C | 0·44 (0·29; 0·64) | 11·24 (7·26; 16·20) | 605·31 (499·62;715·20) | 1·42 (0·84;2·20) | 0·6164 (0·3992; 0·8877) | 19·11 (12·25;28·03) |
| | **2019** | 0·25 (0·20;0·29) | 0·00 (0·00;0·00) D | 0·44 (0·28; 0·65) | 11·07 (6·92; 16·53) | 462·86 (378·22;539·08) | 1·39 (0·83;2·13) | 0·5735 (0·3636; 0·8476) | 17·77 (11·15;26·85) |
| | **Change (%) 2010-2019** | -7·96 (-17·40;1·75) | -9·71 (-21·03;3·22) | 0·40 (-20·41;22·71) | -1·51 (-22·01;24·05) | -23·53 (-31·96;-15·21) | -1·83 (-12·46;9·12) | -6·96 (-26·83;16·73) | -7·02 (-27·20;19·00) |
| **Netherlands** | **2010** | 150·30 (121·42;178·79) | 0·39 (0·24;0·58) | 181·89 (128·14;247·00) | 4216·00 (2976·41; 5823·97) | 860·50 (686·45;1039·63) | 1·93 (1·20;2·87) | 0·6680 (0·4719; 0·9054) | 16·77 (11·92;23·13) |
| | **2019** | 148·65 (120·61;178·49) | 0·29 (0·18;0·43) | 174·67 (121·28;244·55) | 3724·81 (2598·24; 5252·72) | 777·26 (623·09;944·32) | 1·52 (0·95;2·32) | 0·5377 (0·3785; 0·7343) | 13·21 (9·27;18·25) |
| | **Change (%) 2010-2019** | -1·10 (-9·55;8·00) | -26·30 (-35·51;-15·61) | -3·97 (-14·03;7·41) | -11·65 (-20·83;-0·77) | -9·67 (-18·10;-1·29) | -21·28 (-30·15;-11·69) | -19·51 (-27·09;-10·46) | -21·23 (-28·80;-12·61) |
| **Norway** | **2010** | 30·29 (26·16;34·60) | 0·11 (0·08;0·15) | 38·92 (32·18; 47·14) | 1005·82 (831·67; 1214·10) | 581·11 (497·88;667·52) | 1·98 (1·40;2·66) | 0·5108 (0·4241; 0·6179) | 14·66 (12·16;17·61) |
| | **2019** | 27·90 (24·02;31·86) | 0·05 (0·03;0·07) | 21·63 (17·64; 26·65) | 530·08 (430·82; 650·32) | 451·96 (387·54;518·60) | 0·79 (0·52;1·14) | 0·2422 (0·1977; 0·2966) | 6·73 (5·46;8·22) |
| | **Change (%)** | -7·90 | -57·18 | -44·41 | -47·30 | -22·22 | -60·01 | -52·58 | -54·11 |

| | | | | | | | | | |
|---|---|---|---|---|---|---|---|---|---|
| | **2010-2019** | (-10·39;-5·29) | (-64·85;-50·01) | (-47·84;-40·27) | (-50·72;-43·25) | (-24·59;-19·72) | (-66·71;-54·08) | (-55·45;-49·25) | (-57·08;-50·57) |
| **Portugal** | **2010** | 113·32<br>(94·55;133·66) | 0·22<br>(0·13;0·34) | 168·06<br>(115·95;231·81) | 4904·85<br>(3299·17; 6925·37) | 915·56<br>(765·34;1083·90) | 1·74<br>(1·04;2·71) | 0·9790<br>(0·6697; 1·3594) | 31·60<br>(21·09;45·12) |
| | **2019** | 96·49<br>(78·36;116·12) | 0·15<br>(0·09;0·23) | 130·66<br>(90·33;183·43) | 3536·34<br>(2402·53; 4993·15) | 706·41<br>(580·52;845·94) | 1·16<br>(0·69;1·81) | 0·6673<br>(0·4586; 0·9417) | 20·86<br>(14·06;30·10) |
| | **Change (%) 2010-2019** | -14·86<br>(-22·36;-6·05) | -34·58<br>(-43·10;-24·89) | -22·25<br>(-30·70;-13·06) | -27·90<br>(-35·51;-18·82) | -22·84<br>(-29·83;-15·00) | -33·56<br>(-41·73;-24·90) | -31·84<br>(-38·65;-24·03) | -33·97<br>(-40·67;-25·95) |
| **San Marino** | **2010** | 0·23<br>(0·18;0·27) | 0·00<br>(0·00;0·00) E | 0·41<br>(0·24; 0·64) | 8·76<br>(5·33; 13·64) | 611·27<br>(491·73;726·24) | 2·06<br>(1·22;3·19) | 0·7553<br>(0·4512; 1·2029) | 19·10<br>(11·35;30·45) |
| | **2019** | 0·24<br>(0·19;0·28) | 0·00<br>(0·00;0·00) F | 0·51(<br>0·28; 0·83) | 10·83<br>(6·10; 17·67) | 577·14<br>(469·47;683·22) | 2·13<br>(1·27;3·31) | 0·8046<br>(0·4494; 1·2938) | 20·57<br>(11·31;34·13) |
| | **Change (%) 2010-2019** | 4·67<br>(-4·38;14·96) | 4·24<br>(-6·63;17·23) | 25·45<br>(-7·23;69·69) | 23·62<br>(-10·55;69·68) | -5·58<br>(-13·89;3·75) | 3·31<br>(-6·61;14·98) | 6·52<br>(-22·60;46·36) | 7·71<br>(-23·94;48·31) |
| **Spain** | **2010** | 401·91<br>(358·74;447·51) | 1·20<br>(0·88;1·63) | 560·45<br>(421·40;727·96) | 13920·86<br>(10745·70; 17942·10) | 697·86<br>(622·20;776·34) | 1·99<br>(1·46;2·69) | 0·7208<br>(0·5528; 0·9245) | 20·06<br>(15·60;26·02) |
| | **2019** | 345·69<br>(307·91;382·21) | 0·86<br>(0·62;1·20) | 479·60<br>(353·12;643·91) | 11004·06<br>(8435·37; 14443·76) | 548·45<br>(489·14;605·02) | 1·45<br>(1·05;1·99) | 0·5270<br>(0·3972; 0·6927) | 14·15<br>(10·84;18·54) |
| | **Change (%) 2010-2019** | -13·99<br>(-20·49;-6·59) | -28·18<br>(-36·41;-17·55) | -14·43<br>(-23·03;-5·46) | -20·95<br>(-28·19;-13·23) | -21·41<br>(-27·43;-14·84) | -27·50<br>(-35·06;-18·54) | -26·88<br>(-33·11;-19·60) | -29·48<br>(-35·90;-22·67) |
| **Sweden** | **2010** | 53·07<br>(45·32;60·61) | 0·15<br>(0·10;0·21) | 90·45<br>(75·19;108·09) | 2189·71<br>(1824·20; 2637·69) | 559·60<br>(473·82;645·48) | 1·44<br>(0·99;1·98) | 0·5467<br>(0·4535; 0·6547) | 15·10<br>(12·51;18·19) |
| | **2019** | 55·37<br>(47·29;63·69) | 0·15<br>(0·10;0·21) | 93·89<br>(76·87;113·15) | 2148·52<br>(1761·23; 2603·59) | 522·37<br>(442·08;603·25) | 1·34<br>(0·90;1·87) | 0·4993<br>(0·4118; 0·6008) | 13·36<br>(10·93;16·29) |
| | **Change (%) 2010-2019** | 4·33<br>(-1·25;9·83) | -3·35<br>(-13·14;5·89) | 3·80<br>(-5·04;12·99) | -1·88<br>(-10·03;6·45) | -6·65<br>(-12·01;-1·21) | -6·92<br>(-15·74;1·15) | -8·67<br>(-15·81;-1·24) | -11·52<br>(-18·57;-4·34) |
| **Switzerland** | **2010** | 64·26<br>(53·97;73·89) | 0·24<br>(0·15;0·36) | 115·28<br>(80·89;160·29) | 2947·72<br>(2086·75; 4044·14) | 762·18<br>(630·17;889·17) | 2·36<br>(1·52;3·48) | 0·8610<br>(0·6098; 1·1833) | 24·27<br>(17·01;33·43) |
| | **2019** | 68·96<br>(57·47;80·05) | 0·22<br>(0·14;0·32) | 117·95<br>(81·62;162·92) | 2827·16<br>(1997·77; 3868·46) | 730·53<br>(604·07;853·54) | 2·10<br>(1·34;3·09) | 0·7329<br>(0·5132; 1·0169) | 20·09<br>(14·12;27·50) |
| | **Change (%) 2010-2019** | 7·31<br>(-2·75;18·24) | -9·85<br>(-21·63;1·90) | 2·32<br>(-7·61;12·78) | -4·09<br>(-13·92;5·18) | -4·15<br>(-12·64;6·40) | -11·00<br>(-22·30;0·19) | -14·88<br>(-22·77;-7·02) | -17·21<br>(-25·32;-9·21) |
| **United Kingdom** | **2010** | 459·15<br>(397·82;521·43) | 1·36<br>(0·99;1·81) | 762·76<br>(631·08;926·10) | 23873·15<br>(19540·68; 29101·93) | 703·57<br>(605·22;804·62) | 1·95<br>(1·42;2·60) | 0·8339<br>(0·6886; 1·0134) | 28·45<br>(23·06;34·75) |
| | **2019** | 474·83<br>(413·06;539·31) | 1·38<br>(0·97;1·88) | 825·25<br>(679·97;1005·43) | 24702·21<br>(20223·35; 30079·65) | 672·46<br>(579·65;770·61) | 1·99<br>(1·39;2·68) | 0·8141<br>(0·6737; 0·9909) | 27·33<br>(22·15;33·03) |
| | **Change (%) 2010-2019** | 3·41<br>(1·61;5·35) | 1·49<br>(-7·09;11·13) | 8·19<br>(4·81;11·62) | 3·47<br>(0·40;6·82) | -4·42<br>(-6·21;-2·69) | 1·95<br>(-6·44;10·71) | -2·38<br>(-5·00;0·33) | -3·91<br>(-6·45;-1·36) |
| **Liver cancer due to hepatitis** | | | | | | | | | |
| **Andorra** | **2010** | 0·00<br>(0·00;0·00) G | 0·00<br>(0·00;0·00) H | 1·30<br>(0·84; 2·01) | 35·84<br>(22·90; 56·15) | 2·62<br>(1·73;4·04) | 1·43<br>(0·92;2·19) | 1·1021<br>(0·7047;1·6968) | 30·83<br>(19·92;47·96) |
| | **2019** | 0·00<br>(0·00;0·01) | 0·00<br>(0·00;0·00) I | 1·54<br>(0·95; 2·39) | 40·66<br>(25·15; 63·13) | 2·71<br>(1·71;4·07) | 1·46<br>(0·92;2·22) | 1·1224<br>(0·6969;1·7461) | 30·82<br>(19·18;47·12) |
| | **Change (%)** | 14·54 | 16·45 | 18·01 | 13·44 | 3·38 | 2·14 | 1·84 | -0·04 |

| | | | | | | | | | |
|---|---|---|---|---|---|---|---|---|---|
| | **2010-2019** | (-13·32;48·42) | (-10·59;50·74) | (-9·09;51·53) | (-13·16;47·83) | (-20·52;34·89) | (-20·83;32·07) | (-21·15;30·73) | (-23·38;30·03) |
| **Austria** | **2010** | 0·10 (0·07;0·15) | 0·06 (0·04;0·09) | 49·00 (31·98; 73·39) | 1244·24 (832·17; 1831·41) | 0·82 (0·57;1·19) | 0·46 (0·31;0·67) | 0·3495 (0·2288;0·5141) | 9·74 (6·68;14·28) |
| | **2019** | 0·11 (0·07;0·17) | 0·06 (0·04;0·10) | 49·5 7(31·51; 74·99) | 1205·31 (779·82; 1840·79) | 0·76 (0·48;1·14) | 0·41 (0·26;0·62) | 0·3057 (0·1990;0·4585) | 8·28 (5·53;12·35) |
| | **Change (%) 2010-2019** | 3·79 (-20·65;35·46) | 1·18 (-21·12;30·72) | 1·15 (-14·40;17·45) | -3·13 (-18·23;13·54) | -7·34 (-29·26;20·68) | -11·38 (-31·33;15·74) | -12·51 (-25·55;1·85) | -14·93 (-27·78;-0·54) |
| **Belgium** | **2010** | 0·14 (0·10;0·20) | 0·10 (0·07;0·13) | 84·05 (57·39;121·40) | 2112·62 (1495·82; 2897·80) | 0·90 (0·65;1·20) | 0·56 (0·40;0·76) | 0·4652 (0·3240;0·6537) | 13·03 (9·46;17·56) |
| | **2019** | 0·18 (0·11;0·26) | 0·11 (0·07;0·17) | 96·29 (64·47;139·21) | 2315·58 (1577·25; 3330·05) | 1·01 (0·68;1·48) | 0·59 (0·37;0·87) | 0·4702 (0·3194;0·6673) | 12·89 (9·17;18·11) |
| | **Change (%) 2010-2019** | 22·08 (-8·14;58·80) | 16·61 (-11·07;50·28) | 14·57 (1·99;28·69) | 9·61 (-3·15;24·62) | 12·04 (-16·69;46·83) | 4·50 (-20·62;35·24) | 1·08 (-9·94;13·62) | -1·11 (-12·11;12·13) |
| **Cyprus** | **2010** | 0·01 (0·01;0·01) | 0·01 (0·00;0·01) | 5·71 (3·79; 8·40) | 149·67 (102·73; 213·21) | 0·74 (0·53;1·04) | 0·48 (0·33;0·69) | 0·4107 (0·2789;0·6002) | 10·66 (7·41;14·98) |
| | **2019** | 0·01 (0·01;0·02) | 0·01 (0·01;0·01) | 7·33 (4·78; 10·87) | 184·39 (125·58; 265·37) | 0·78 (0·54;1·09) | 0·47 (0·32;0·67) | 0·3898 (0·2615;0·5691) | 10·12 (6·87;14·45) |
| | **Change (%) 2010-2019** | 34·79 (9·16;63·42) | 29·79 (6·26;55·32) | 28·42 (7·00;52·07) | 23·20 (3·05;46·38) | 4·67 (-15·17;26·08) | -2·12 (-20·03;16·37) | -5·08 (-19·43;11·81) | -5·07 (-20·88;12·23) |
| **Denmark** | **2010** | 0·09 (0·06;0·13) | 0·05 (0·04;0·07) | 40·59 (27·52; 58·88) | 1065·39 (732·17; 1505·98) | 1·09 (0·78;1·51) | 0·60 (0·42;0·84) | 0·4533 (0·3098;0·6406) | 12·86 (9·03;17·85) |
| | **2019** | 0·10 (0·06;0·16) | 0·06 (0·04;0·09) | 46·64 (30·60; 69·17) | 1161·53 (779·10; 1674·46) | 1·13 (0·74;1·72) | 0·61 (0·38;0·93) | 0·4525 (0·3031;0·6525) | 12·66 (8·71;17·84) |
| | **Change (%) 2010-2019** | 14·44 (-14·50;50·32) | 13·94 (-13·15;48·22) | 14·90 (0·23;30·64) | 9·02 (-5·40;24·23) | 4·36 (-21·11;38·83) | 0·95 (-22·59;32·16) | -0·19 (-12·52;13·29) | -1·53 (-14·34;12·42) |
| **Finland** | **2010** | 0·13 (0·09;0·18) | 0·07 (0·05;0·10) | 47·74 (31·64; 69·17) | 1162·20 (796·66; 1631·28) | 1·57 (1·13;2·13) | 0·73 (0·51;1·02) | 0·5000 (0·3400;0·7144) | 13·65 (9·79;18·67) |
| | **2019** | 0·16 (0·10;0·25) | 0·08 (0·05;0·13) | 56·76 (36·66; 86·11) | 1279·41 (860·07; 1890·11) | 1·69 (1·11;2·55) | 0·76 (0·49;1·17) | 0·5088 (0·3459;0·7349) | 13·51 (9·57;18·93) |
| | **Change (%) 2010-2019** | 20·46 (-8·51;59·71) | 18·87 (-9·24;56·51) | 18·89 (3·28;36·30) | 10·09 (-4·41;25·85) | 7·70 (-18·89;42·41) | 3·76 (-21·15;35·83) | 1·75 (-10·23;14·87) | -1·07 (-13·40;12·39) |
| **France** | **2010** | 1·58 (1·13;2·13) | 1·02 (0·72;1·42) | 872·82 (604·45;1239·33) | 22307·18 (15991·53; 30803·66) | 1·72 (1·26;2·30) | 1·04 (0·75;1·43) | 0·8467 (0·5919;1·1794) | 23·88 (17·25;32·27) |
| | **2019** | 1·68 (1·05;2·58) | 1·06 (0·65;1·65) | 897·35 (585·65;1331·06) | 21936·66 (14247·43; 32085·36) | 1·67 (1·08;2·50) | 0·96 (0·60;1·47) | 0·7598 (0·4988;1·1121) | 21·14 (14·30;30·43) |
| | **Change (%) 2010-2019** | 6·78 (-22·74;43·72) | 3·27 (-23·82;38·48) | 2·81 (-12·34;20·24) | -1·66 (-16·95;15·18) | -2·45 (-29·69;32·57) | -8·19 (-32·86;24·81) | -10·27 (-23·33;4·85) | -11·48 (-24·87;4·77) |
| **Germany** | **2010** | 1·40 (0·98;1·94) | 0·82 (0·56;1·15) | 651·16 (443·89;923·49) | 15388·64 (10771·05; 21435·86) | 1·02 (0·74;1·37) | 0·55 (0·39;0·76) | 0·4169 (0·2892;0·5837) | 11·13 (8·02;15·33) |
| | **2019** | 1·57 (1·00;2·36) | 0·89 (0·56;1·36) | 693·17 (458·19;1016·61) | 16019·99 (10844·49; 23195·30) | 1·06 (0·69;1·57) | 0·55 (0·35;0·83) | 0·4019 (0·2720;0·5877) | 10·74 (7·55;15·34) |
| | **Change (%) 2010-2019** | 12·07 (-16·11;46·57) | 8·25 (-18·22;41·20) | 6·45 (-7·26;21·15) | 4·10 (-9·39;18·77) | 3·93 (-22·66;35·67) | -0·79 (-25·18;29·33) | -3·60 (-15·73;9·46) | -3·54 (-15·84;9·74) |

| | | | | | | | | | |
|---|---|---|---|---|---|---|---|---|---|
| **Greece** | **2010** | 0·32 (0·24;0·40) | 0·23 (0·17;0·29) | 204·43 (151·20;267·09) | 4497·64 (3450·69; 5701·04) | 1·78 (1·39;2·21) | 1·16 (0·90;1·47) | 1·0005 (0·7556;1·2910) | 24·89 (19·42;31·05) |
| | **2019** | 0·37 (0·26;0·52) | 0·27 (0·18;0·38) | 249·03 (182·39;333·31) | 5306·95 (3972·34; 6905·90) | 2·00 (1·41;2·77) | 1·31 (0·92;1·84) | 1·1311 (0·8455;1·4805) | 28·39 (21·97;35·89) |
| | **Change (%) 2010-2019** | 16·19 (-10·65;49·66) | 19·55 (-7·28;54·05) | 21·82 (8·14;35·34) | 17·99 (6·01;30·89) | 12·18 (-13·62;45·82) | 13·01 (-12·62;46·06) | 13·06 (1·19;25·78) | 14·07 (2·90;26·81) |
| **Iceland** | **2010** | 0·00 (0·00;0·01) | 0·00 (0·00;0·00) L | 1·68 (1·17; 2·43) | 44·30 (31·58; 61·65) | 1·02 (0·75;1·38) | 0·53 (0·38;0·74) | 0·4016 (0·2803;0·5821) | 11·07 (7·91;15·41) |
| | **2019** | 0·01 (0·00;0·01) | 0·00 (0·00;0·00) M | 2·17 (1·46; 3·20) | 55·79 (39·00; 78·45) | 1·11 (0·77;1·56) | 0·58 (0·40;0·83) | 0·4215 (0·2921;0·6101) | 11·69 (8·27;16·23) |
| | **Change (%) 2010-2019** | 28·26 (4·85;56·48) | 32·26 (9·21;59·72) | 29·25 (10·50;51·54) | 25·93 (6·80;48·25) | 9·19 (-10·25;33·20) | 9·36 (-9·20;31·43) | 4·97 (-10·51;22·35) | 5·62 (-10·19;24·51) |
| **Ireland** | **2010** | 0·05 (0·04;0·07) | 0·03 (0·02;0·04) | 25·3 1(17·13; 36·12) | 669·60 (475·97; 928·43) | 0·95 (0·69;1·29) | 0·55 (0·38;0·76) | 0·4421 (0·3009;0·6273) | 11·98 (8·58;16·58) |
| | **2019** | 0·07 (0·04;0·10) | 0·04 (0·02;0·06) | 29·64 (19·75; 43·82) | 737·42(505·38; 1069·15) | 1·00 (0·64;1·53) | 0·54 (0·34;0·82) | 0·4110(0·2767;0·6 047) | 10·87 (7·58;15·49) |
| | **Change (%) 2010-2019** | 25·63 (-7·78;65·65) | 21·41 (-8·23;58·76) | 17·11 (1·92;32·34) | 10·13 (-4·54;26·93) | 5·23 (-21·83;39·35) | -1·62 (-25·94;28·47) | -7·05 (-18·60;4·74) | -9·30 (-21·23;3·70) |
| **Israel** | **2010** | 0·06 (0·04;0·07) | 0·04 (0·03;0·05) | 35·16 (24·27; 48·90) | 942·72 (670·92; 1288·75) | 0·67 (0·48;0·89) | 0·46 (0·33;0·63) | 0·4114 (0·2861;0·5704) | 11·38 (8·14;15·63) |
| | **2019** | 0·07 (0·05;0·11) | 0·05 (0·03;0·08) | 44·78 (30·08; 65·64) | 1164·42 (814·88; 1654·34) | 0·72 (0·47;1·10) | 0·48 (0·31;0·73) | 0·4092 (0·2793;0·5893) | 11·27 (7·92;16·02) |
| | **Change (%) 2010-2019** | 34·69 (1·06;77·65) | 30·02 (-1·45;72·30) | 27·38 (12·72;43·35) | 23·52 (8·62;39·96) | 8·33 (-18·30;43·91) | 2·73 (-21·94;35·87) | -0·54 (-11·82;11·37) | -0·95 (-12·49;12·76) |
| **Italy** | **2010** | 1·41 (1·21;1·66) | 0·74 (0·63;0·87) | 568·14 (482·20;675·02) | 13684·65 (11713·88; 15963·38) | 1·53 (1·30;1·79) | 0·72 (0·62;0·83) | 0·5057 (0·4305;0·5937) | 13·97 (12·01;16·21) |
| | **2019** | 1·63 (1·20;2·15) | 0·85 (0·63;1·10) | 654·36 (535·99;798·62) | 15464·68 (12765·47; 18745·19) | 1·63 (1·21;2·13) | 0·75 (0·56;0·98) | 0·5249 (0·4329;0·6370) | 14·44 (12·00;17·28) |
| | **Change (%) 2010-2019** | 14·97 (-9·91;45·86) | 15·00 (-7·34;44·13) | 15·18 (5·06;24·18) | 13·01 (2·52;23·28) | 6·47 (-16·32;35·26) | 4·85 (-16·24;32·63) | 3·79 (-5·61;12·40) | 3·34 (-6·39;12·73) |
| **Luxembourg** | **2010** | 0·01 (0·00;0·01) | 0·00 (0·00;0·01) | 3·58 (2·43; 5·17) | 91·73 (62·99; 129·36) | 1·04 (0·74;1·45) | 0·60 (0·42;0·86) | 0·4858 (0·3311;0·6941) | 13·18 (9·17;18·53) |
| | **2019** | 0·01 (0·01;0·01) | 0·00 (0·00;0·01) | 3·94 (2·46; 6·01) | 99·90 (64·79; 150·35) | 0·93 (0·60;1·37) | 0·51 (0·33;0·77) | 0·4143 (0·2625;0·6266) | 11·19 (7·37;16·58) |
| | **Change (%) 2010-2019** | 12·89 (-12·69;46·70) | 8·86 (-15·69;41·91) | 10·02 (-11·35;36·13) | 8·90 (-12·79;35·04) | -10·76 (-30·35;15·53) | -14·98 (-34·21;10·59) | -14·72 (-31·29;6·06) | -15·14 (-31·46;5·56) |
| **Malta** | **2010** | 0·00 (0·00;0·00) N | 0·00 (0·00;0·00) O | 2·09 (1·42; 3·10) | 54·06 (37·76; 77·53) | 0·55 (0·39;0·76) | 0·35 (0·25;0·50) | 0·3017 (0·2097;0·4394) | 8·39 (6·02;11·60) |
| | **2019** | 0·00 (0·00;0·01) | 0·00 (0·00;0·00) P | 2·40 (1·59; 3·56) | 58·49 (39·64; 85·77) | 0·59 (0·41;0·84) | 0·36 (0·25;0·52) | 0·2875 (0·1946;0·4172) | 8·04 (5·63;11·32) |
| | **Change (%) 2010-2019** | 21·01 (-2·63;48·50) | 18·27 (-4·89;44·30) | 14·94 (-3·84;34·49) | 8·18 (-9·62;26·56) | 8·21 (-12·58;32·54) | 1·06 (-17·30;22·35) | -4·70 (-18·85;11·49) | -4·25 (-19·07;12·31) |
| **Monaco** | **2010** | 0·00 | 0·00 | 0·80 | 19·17 | 2·52 | 1·40 | 1·0781 | 30·42 |

| | | | | | | | | | |
|---|---|---|---|---|---|---|---|---|---|
| | | (0·00;0·00) Q | (0·00;0·00) R | (0·51; 1·21) | (12·43; 28·43) | (1·68;3·55) | (0·92;2·03) | (0·7010;1·6001) | (20·19;43·73) |
| | **2019** | 0·00<br>(0·00;0·00) S | 0·00<br>(0·00;0·00) T | 0·83<br>(0·53; 1·23) | 19·81<br>(12·58; 29·54) | 2·52<br>(1·67;3·59) | 1·35<br>(0·88;1·97) | 1·0203<br>(0·6598;1·5003) | 28·81<br>(18·50;41·83) |
| | **Change (%) 2010-2019** | 6·93<br>(-17·14;39·15) | 5·28<br>(-17·19;34·20) | 3·96<br>(-18·40;31·47) | 3·36<br>(-19·73;33·97) | 0·02<br>(-23·27;30·57) | -3·29<br>(-24·64;24·33) | -5·36<br>(-26·35;21·34) | -5·27<br>(-27·01;23·18) |
| **Netherlands** | **2010** | 0·18<br>(0·13;0·25) | 0·10<br>(0·07;0·15) | 83·29<br>(58·36;119·52) | 2178·85<br>(1575·74; 3025·66) | 0·80<br>(0·60;1·07) | 0·42<br>(0·31;0·58) | 0·3180<br>(0·2266;0·4479) | 9·08<br>(6·71;12·29) |
| | **2019** | 0·23<br>(0·15;0·35) | 0·13<br>(0·08;0·20) | 104·52<br>(69·95;151·38) | 2588·80<br>(1811·20; 3647·73) | 0·94<br>(0·63;1·38) | 0·47<br>(0·31;0·70) | 0·3413<br>(0·2397;0·4802) | 9·71<br>(7·05;13·22) |
| | **Change (%) 2010-2019** | 27·27<br>(-5·43;64·34) | 26·12<br>(-4·35;61·84) | 25·49<br>(11·71;39·26) | 18·82<br>(5·11;33·41) | 17·50<br>(-12·83;51·65) | 11·23<br>(-15·91;42·72) | 7·30<br>(-3·37;18·89) | 6·93<br>(-4·53;19·97) |
| **Norway** | **2010** | 0·06<br>(0·05;0·07) | 0·03<br>(0·03;0·04) | 26·07<br>(21·90; 30·96) | 696·32<br>(589·60; 825·90) | 0·89<br>(0·75;1·04) | 0·47<br>(0·40;0·56) | 0·3580<br>(0·3023;0·4234) | 10·49<br>(8·89;12·30) |
| | **2019** | 0·06<br>(0·05;0·08) | 0·04<br>(0·03;0·05) | 28·56<br>(22·91; 35·66) | 745·31<br>(604·99; 921·12) | 0·88<br>(0·67;1·13) | 0·45<br>(0·35;0·58) | 0·3340<br>(0·2692;0·4136) | 9·76<br>(7·96;12·00) |
| | **Change (%) 2010-2019** | 11·80<br>(-10·39;37·03) | 9·26<br>(-10·29;32·51) | 9·57<br>(-0·62;23·64) | 7·04<br>(-3·22;21·89) | -1·45<br>(-21·58;21·48) | -5·72<br>(-23·12;14·74) | -6·69<br>(-14·86;5·71) | -6·95<br>(-15·70;6·69) |
| **Portugal** | **2010** | 0·15<br>(0·11;0·22) | 0·12<br>(0·08;0·16) | 106·09<br>(72·06;150·45) | 2918·23<br>(2002·77; 4118·58) | 0·98<br>(0·69;1·35) | 0·68<br>(0·48;0·95) | 0·6000<br>(0·4158;0·8441) | 18·37<br>(12·82;25·73) |
| | **2019** | 0·19<br>(0·12;0·30) | 0·14<br>(0·08;0·21) | 124·99<br>(84·17;183·11) | 3281·38<br>(2216·85; 4809·11) | 1·12<br>(0·71;1·72) | 0·74<br>(0·46;1·14) | 0·6294<br>(0·4315;0·9090) | 18·92<br>(13·09;27·06) |
| | **Change (%) 2010-2019** | 23·18<br>(-8·82;61·22) | 19·05<br>(-10·61;54·43) | 17·82<br>(3·73;33·18) | 12·44<br>(-2·47;28·78) | 14·27<br>(-15·15;50·04) | 7·63<br>(-19·39;40·69) | 4·91<br>(-8·06;18·81) | 2·98<br>(-10·65;18·41) |
| **San Marino** | **2010** | 0·00<br>(0·00;0·00) U | 0·00<br>(0·00;0·00) V | 0·16<br>(0·09; 0·26) | 3·98<br>(2·35; 6·50) | 0·76<br>(0·51;1·11) | 0·41<br>(0·27;0·63) | 0·3255<br>(0·1911;0·5348) | 9·18<br>(5·49;14·75) |
| | **2019** | 0·00<br>(0·00;0·00) Z | 0·00<br>(0·00;0·00) AA | 0·20<br>(0·11; 0·34) | 4·89<br>(2·73; 8·17) | 0·87<br>(0·57;1·30) | 0·46<br>(0·29;0·70) | 0·3484<br>(0·1919;0·5832) | 9·88<br>(5·57;16·60) |
| | **Change (%) 2010-2019** | 28·69<br>(-1·17;65·66) | 26·64<br>(-2·45;60·62) | 24·10<br>(-11·62;71·66) | 23·13<br>(-14·68;71·13) | 14·24<br>(-13·06;48·24) | 10·28<br>(-14·78;40·61) | 7·04<br>(-25·31;48·00) | 7·57<br>(-26·59;50·38) |
| **Spain** | **2010** | 1·00<br>(0·70;1·38) | 0·54<br>(0·38;0·76) | 425·55<br>(291·98;609·32) | 11125·94<br>(7819·50; 15557·39) | 1·51<br>(1·08;2·08) | 0·77<br>(0·54;1·07) | 0·5698<br>(0·3904;0·7997) | 16·48<br>(11·58;22·75) |
| | **2019** | 1·02<br>(0·65;1·55) | 0·58<br>(0·36;0·90) | 467·28<br>(310·04;685·01) | 11798·48<br>(7929·99; 16934·19) | 1·43<br>(0·92;2·14) | 0·74<br>(0·47;1·14) | 0·5560<br>(0·3727;0·8094) | 15·80<br>(10·92;22·51) |
| | **Change (%) 2010-2019** | 2·58<br>(-24·34;37·67) | 6·45<br>(-20·29;39·14) | 9·80<br>(-3·18;25·23) | 6·04<br>(-8·26;22·82) | -5·35<br>(-30·33;27·47) | -4·17<br>(-28·80;25·83) | -2·42<br>(-14·08;11·32) | -4·10<br>(-16·26;9·91) |
| **Sweden** | **2010** | 0·03<br>(0·02;0·04) | 0·03<br>(0·02;0·03) | 25·43<br>(19·28; 32·78) | 656·08<br>(499·14; 841·77) | 0·23<br>(0·18;0·29) | 0·17<br>(0·13;0·22) | 0·1618<br>(0·1232;0·2056) | 4·72<br>(3·67;5·98) |
| | **2019** | 0·04<br>(0·03;0·06) | 0·03<br>(0·02;0·04) | 32·70(24·64; 42·28) | 807·22(616·13;<br>1048·33) | 0·27<br>(0·20;0·36) | 0·20<br>(0·15;0·27) | 0·1836(0·1402;0·2<br>364) | 5·27<br>(4·12;6·81) |
| | **Change (%) 2010-2019** | 30·47<br>(7·98;56·86) | 29·57<br>(7·13;56·41) | 28·56<br>(16·18;41·03) | 23·04<br>(10·65;35·90) | 18·72<br>(-1·46;42·61) | 15·70<br>(-4·32;39·71) | 13·48<br>(2·75;24·92) | 11·47<br>(0·26;22·68) |
| **Switzerland** | **2010** | 0·22<br>(0·15;0·31) | 0·11<br>(0·08;0·16) | 81·53<br>(55·86;117·66) | 2096·25<br>(1468·25; 2944·05) | 1·83<br>(1·29;2·54) | 0·89<br>(0·62;1·25) | 0·6239<br>(0·4288;0·8926) | 17·40<br>(12·34;24·18) |

| | | | | | | | | | |
|---|---|---|---|---|---|---|---|---|---|
| | **2019** | 0·25<br>(0·15;0·38) | 0·13<br>(0·08;0·19) | 93·05<br>(61·03;135·98) | 2264·97<br>(1514·97; 3239·04) | 1·76<br>(1·10;2·71) | 0·84<br>(0·53;1·29) | 0·5898<br>(0·3929;0·8514) | 15·95<br>(10·89;22·56) |
| | **Change (%) 2010-2019** | 12·98<br>(-19·08;52·12) | 13·29<br>(-15·54;51·36) | 14·12<br>(-0·15;30·75) | 8·05<br>(-6·42;25·15) | -3·48<br>(-30·37;29·34) | -4·95<br>(-29·73;27·62) | -5·47<br>(-17·25;8·23) | -8·31<br>(-20·64;5·52) |
| **United Kingdom** | **2010** | 1·14<br>(0·97;1·34) | 0·62<br>(0·52;0·73) | 472·78<br>(392·19;561·62) | 11892·21<br>(9977·89; 14157·47) | 1·28<br>(1·09;1·50) | 0·65<br>(0·55;0·76) | 0·4714<br>(0·3948;0·5580) | 13·25<br>(11·14;15·69) |
| | **2019** | 1·52<br>(1·14;1·95) | 0·80<br>(0·61;1·03) | 608·12<br>(499·02;729·81) | 14663·41<br>(12193·80; 17520·22) | 1·54<br>(1·19;1·96) | 0·75<br>(0·57;0·95) | 0·5309<br>(0·4420;0·6335) | 14·63<br>(12·26;17·29) |
| | **Change (%) 2010-2019** | 33·43<br>(8·36;63·51) | 30·25<br>(6·84;57·32) | 28·63<br>(22·15;35·48) | 23·30<br>(16·83;29·89) | 20·42<br>(-2·54;48·12) | 15·47<br>(-5·30;40·20) | 12·64<br>(6·88;18·59) | 10·39<br>(4·58;16·46) |

UI= Uncertainty Interval

A: 1·69(1·03;2·65); B:1·39(0·84;2·15); C:0·58(0·34;0·92); D: 0·53(0·31;0·81): E: 0·77(0·45;1·19); F: 0·80(0·47;1·24);  G: 3·02(1·95;4·71); H: 1·67(1·07;2·59): I: 1·94(1·21;3·01); L: 2·16(1·53;3·05); M: 2·86(1·98;4·12); N: 3·41(2·43;4·84); O: 2·37(1·65;3·44); P: 2·80(1·84;4·18); Q: 1·51(0·99;2·21); R: 0·96(0·62;1·42); S: 1·62(1·03;2·37); T: 1·01(0·64;1·48); U: 0·32(0·21;0·48); V: 0·19(0·12;0·29); Z: 0·41(0·26;0·62); AA: 0·24(0·15;0·38)

**Supplement Table F**. Prevalence, incidence, Mortality and DALYs attributable to HCV in 2010 and 2019, by European region.

| | | N in thousand (95%UI) | | | | Age- standardized rate x 100,000 ( 95%UI) | | | |
|---|---|---|---|---|---|---|---|---|---|
| **Acute hepatitis** | | | | | | | | | |
| | **Year** | **Prevalence** | **Incidence** | **Mortality** | **DALYs** | **Prevalence** | **Incidence** | **Mortality** | **DALYs** |
| **Eastern Europe** | **2010** | 17·49 | 151·54 | 0·02 | 1·37 | 9·84 | 85·27 | 0·01 | 0·68 |
| | | (15·00;20·68) | (129·99;179·21) | (0·01;0·03) | (0·82;1·98) | ( 8·55;11·53) | (74·08; 99·94) | (0·01;0·02) | (0·46;1·06) |
| | **2019** | 17·69 | 153·29 | 0·01 | 0·88 | 9·71 | 84·16 | 0·01 | 0·44 |
| | | (15·14;20·99) | (131·25;181·89) | (0·01;0·02) | (0·54;1·40) | ( 8·44;11·41) | (73·19; 98·86) | (0·00;0·01) | (0·28;0·71) |
| | **Change (%) 2010-2019** | 1·15 | 1·15 | -44·52 | -35·72 | -1·30 | -1·30 | -44·17 | -34·30 |
| | | ( -2·46; 4·26) | ( -2·46; 4·26) | (-65·21;-22·67) | (-54·62;-17·60) | ( -2·35; -0·23) | ( -2·35; -0·23) | (-65·29;-23·63) | (-52·31;-17·17) |
| **Central Europe** | **2010** | 5·76 | 49·93 | 0·00 | 0·23 | 5·51 | 47·77 | 0·00 | 0·22 |
| | | ( 5·00; 6·70) | ( 43·35; 58·05) | (0·00;0·01) A | (0·16;0·35) | ( 4·91; 6·27) | (42·60; 54·30) | (0·00;0·01) | (0·16;0·35) |
| | **2019** | 5·45 | 47·25 | 0·00 | 0·18 | 5·15 | 44·66 | 0·00 | 0·17 |
| | | ( 4·69; 6·35) | ( 40·64; 55·05) | (0·00;0·00) B | (0·12;0·28) | ( 4·55; 5·92) | (39·45; 51·31) | (0·00;0·00) C | (0·12;0·27) |
| | **Change (%) 2010-2019** | -5·37 | -5·37 | -23·88 | -21·21 | -6·51 | -6·51 | -28·02 | -21·71 |
| | | ( -8·64; -2·13) | ( -8·64; -2·13) | (-38·50; -6·48) | (-35·28; -8·49) | ( -8·84; -3·90) | ( -8·84; -3·90) | (-44·21; -9·25) | (-38·99; -6·68) |
| **Western Europe** | **2010** | 26·67 | 231·15 | 0·04 | 1·29 | 5·38 | 46·59 | 0·01 | 0·24 |
| | | (22·91;30·92) | (198·55;268·01) | (0·02;0·07) | (0·92;2·05) | ( 4·74; 6·16) | (41·10; 53·41) | (0·00;0·01) | (0·17;0·38) |
| | **2019** | 25·86 | 224·08 | 0·03 | 1·02 | 4·72 | 40·91 | 0·00 | 0·18 |
| | | (22·08;30·15) | (191·32;261·30) | (0·02;0·05) | (0·71;1·65) | ( 4·13; 5·45) | (35·83; 47·24) | (0·00;0·01) | (0·12;0·28) |
| | **Change (%) 2010-2019** | -3·06 | -3·06 | -24·76 | -20·87 | -12·20 | -12·20 | -32·28 | -26·03 |
| | | ( -6·36; 0·39) | ( -6·36; 0·39) | (-35·70;-11·86) | (-29·47;-10·49) | (-14·08;-10·27) | (-14·08;-10·27) | (-41·79;-19·77) | (-34·69;-16·47) |
| **Cirrhosis and other chronic liver diseases due to hepatitis** | | | | | | | | | |
| | **Year** | **Prevalence** | **Incidence** | **Mortality** | **DALYs** | **Prevalence** | **Incidence** | **Mortality** | **DALYs** |
| **Eastern Europe** | **2010** | 4872·94 | 10·29 | 13·86 | 523·02 | 2026·94 | 4·51 | 4·93 | 193·99 |
| | | (3945·61; 5990·63) | ( 6·65;14·71) | (11·70;16·50) | (442·14; 613·96) | (1635·00;2497·29) | ( 2·91; 6·52) | (4·17; 5·81) | (163·35;228·06) |
| | **2019** | 4836·09 | 11·00 | 13·25 | 490·16 | 2000·76 | 4·80 | 4·53 | 177·85 |
| | | (3917·88; 5950·30) | ( 6·66;16·49) | (10·78;16·12) | (399·69; 597·53) | (1614·93;2474·14) | ( 2·98; 7·08) | (3·71; 5·52) | (145·20;216·73) |
| | **Change (%) 2010-2019** | -0·76 | 6·89 | -4·43 | -6·28 | -1·29 | 6·61 | -8·17 | -8·32 |
| | | ( -2·21; 0·76) | ( -6·29; 18·61) | (-15·10; 7·11) | (-17·01; 5·05) | ( -2·62; 0·09) | ( -3·00; 14·92) | (-18·34; 2·72) | (-18·37; 2·29) |
| **Central Europe** | **2010** | 1439·80 | 8·94 | 6·59 | 203·21 | 1069·45 | 6·77 | 3·79 | 123·08 |
| | | (1191·19; 1721·31) | ( 6·15;12·35) | ( 5·04; 8·66) | (156·90; 264·94) | ( 879·82;1288·13) | ( 4·67; 9·34) | (2·95; 4·92) | ( 95·96;158·49) |
| | **2019** | 1346·81 | 8·16 | 6·07 | 180·02 | 991·12 | 6·06 | 3·26 | 105·03 |
| | | (1100·12; 1640·50) | ( 5·56;11·39) | ( 4·51; 8·08) | (133·24; 236·38) | ( 801·70;1222·11) | ( 4·20; 8·47) | (2·43; 4·29) | ( 79·23;137·02) |
| | **Change (%) 2010-2019** | -6·46 | -8·70 | -7·81 | -11·41 | -7·32 | -10·41 | -14·01 | -14·66 |
| | | ( -9·28; -2·88) | (-14·87; -3·21) | (-19·91; 4·06) | (-23·32; 0·40) | (-10·54; -3·73) | (-16·11; -5·17) | (-25·07; -2·91) | (-25·92; -3·55) |
| **Western Europe** | **2010** | 4642·25 | 31·61 | 14·80 | 372·86 | 823·41 | 6·26 | 2·06 | 58·55 |
| | | (3842·30; 5612·94) | (23·03;41·60) | (11·71;18·58) | (294·63; 477·84) | ( 680·12; 997·83) | ( 4·63; 8·26) | (1·65; 2·60) | ( 45·83; 74·98) |
| | **2019** | 4360·49 | 26·97 | 14·72 | 346·39 | 710·17 | 5·45 | 1·79 | 49·60 |
| | | (3595·84; 5272·33) | (19·57;35·78) | (11·48;18·40) | (272·37; 440·86) | ( 582·63; 871·33) | ( 3·98; 7·22) | (1·41; 2·25) | ( 38·73; 63·24) |
| | **Change (%) 2010-2019** | -6·07 | -14·70 | -0·52 | -7·10 | -13·75 | -13·06 | -13·44 | -15·29 |
| | | ( -8·36; -3·75) | (-19·60; -9·73) | ( -4·65; 4·29) | (-10·90; -2·90) | (-15·48;-12·13) | (-17·83; -8·86) | (-16·49;-10·05) | (-18·41;-11·94) |

| | Year | Liver cancer due to hepatitis | | | | | | | |
|---|---|---|---|---|---|---|---|---|---|
| | | Prevalence | Incidence | Mortality | DALYs | Prevalence | Incidence | Mortality | DALYs |
| **Eastern Europe** | **2010** | 2·11<br>( 1·81; 2·42) | 2·15<br>( 1·87; 2·45) | 2·32<br>( 2·02; 2·64) | 48·31<br>( 41·04; 55·57) | 0·66<br>(0·57;0·76) | 0·68<br>(0·59;0·77) | 0·73<br>(0·64;0·83) | 15·30<br>(12·98;17·71) |
| | **2019** | 2·48<br>( 2·02; 2·98) | 2·51<br>( 2·07; 3·01) | 2·72<br>( 2·28; 3·21) | 54·37<br>( 44·57; 65·51) | 0·71<br>(0·58;0·85) | 0·71<br>(0·59;0·85) | 0·77<br>(0·65;0·91) | 15·78<br>(13·01;18·96) |
| | **Change (%) 2010-2019** | 17·36<br>( 4·10; 33·51) | 16·49<br>( 3·91; 31·98) | 17·02<br>( 4·21; 32·01) | 12·54<br>( -0·40; 29·39) | 7·06<br>( -4·91; 22·35) | 5·47<br>( -6·06; 19·75) | 5·19<br>( -6·09; 18·90) | 3·12<br>( -9·06; 18·60) |
| **Central Europe** | **2010** | 1·74<br>( 1·27; 2·24) | 1·75<br>( 1·28; 2·24) | 1·88<br>( 1·39; 2·40) | 37·25<br>( 26·95; 48·25) | 0·91<br>(0·66;1·17) | 0·90<br>(0·67;1·15) | 0·97<br>(0·73;1·23) | 19·58<br>(14·30;25·44) |
| | **2019** | 1·77<br>( 1·27; 2·41) | 1·78<br>( 1·30; 2·39) | 1·93<br>( 1·41; 2·57) | 36·48<br>( 25·65; 49·75) | 0·82<br>(0·58;1·11) | 0·81<br>(0·59;1·08) | 0·86<br>(0·63;1·15) | 17·19<br>(12·07;23·31) |
| | **Change (%) 2010-2019** | 1·82<br>(-11·54; 17·58) | 1·76<br>(-10·97; 16·49) | 2·40<br>(-10·77; 17·84) | -2·07<br>(-15·43; 14·43) | -9·55<br>(-21·67; 4·57) | -10·75<br>(-22·22; 2·53) | -11·05<br>(-22·42; 2·41) | -12·23<br>(-24·34; 2·47) |
| **Western Europe** | **2010** | 22·97<br>(19·08;26·99) | 16·48<br>(13·76;19·30) | 15·25<br>(12·76;17·86) | 277·01<br>(229·31;326·96) | 3·04<br>(2·51;3·60) | 2·06<br>(1·72;2·42) | 1·84<br>(1·54;2·14) | 36·80<br>(30·11;43·73) |
| | **2019** | 26·71<br>(21·28;33·09) | 18·95<br>(15·19;23·28) | 17·57<br>(14·48;20·79) | 308·84<br>(250·96;370·80) | 3·11<br>(2·47;3·86) | 2·05<br>(1·63;2·55) | 1·81<br>(1·49;2·15) | 36·01<br>(28·98;43·60) |
| | **Change (%) 2010-2019** | 16·29<br>( 0·94; 32·29) | 15·00<br>( 0·70; 31·02) | 15·17<br>( 9·77; 20·90) | 11·49<br>( 5·67; 17·79) | 2·37<br>(-11·32; 17·09) | -0·32<br>(-13·35; 14·26) | -1·41<br>( -6·22; 3·62) | -2·14<br>( -7·38; 3·61) |

UI= Uncertainty Interval

A: 3·83 (2·67;6·55); B: 2·92 (1·77;4·86); C: 0·0021 (0·0014;0·0035)

**Supplemental Table G·** Prevalence, incidence, mortality and DALYs attributable to HCV in 2010 and 2019, by Central Europe countries·

| | | N in thousands (95%UI) | | N in thousands (95%UI) | | Age- standardized rate x 100,000 (95%UI) | | | |
|---|---|---|---|---|---|---|---|---|---|
| | **Year** | **Prevalence** | **Incidence** | **Mortality** | **DALYs** | **Prevalence** | **Incidence** | **Mortality** | **DALYs** |
| **Acute hepatitis** | | | | | | | | | |
| **Albania** | **2010** | 0·13 (0·11;0·15) | 1·13 (0·98;1·33) | 0·17 (0·07;0·26) | 8·63 (4·40; 12·16) | 4·90 (4·24;5·67) | 42·46 (36·73;49·13) | 0·0056 (0·0024;0·0081) | 0·32 (0·16;0·45) |
| | **2019** | 0·13 (0·11;0·15) | 1·10 (0·95;1·29) | 0·14 (0·06;0·22) | 6·90 (3·43; 10·14) | 4·77 (4·19;5·54) | 41·35 (36·29;48·02) | 0·0044 (0·0019;0·0067) | 0·26 (0·13;0·40) |
| | **Change (%) 2010-2019** | -2·59 (-9·71;4·44) | -2·59 (-9·71;4·44) | -14·34 (-42·80;23·27) | -20·01 (-40·92;6·45) | -2·62 (-8·05;3·14) | -2·62 (-8·05;3·14) | -21·68 (-46·95;11·96) | -17·61 (-39·74;11·88) |
| **Bosnia and Herzegovina** | **2010** | 0·19 (0·16;0·22) | 1·63 (1·40;1·92) | 0·25 (0·11;0·41) | 15·20 (6·65; 23·09) | 5·49 (4·81;6·30) | 47·55 (41·65;54·58) | 0·0070 (0·0027;0·0115) | 0·51 (0·20;0·82) |
| | **2019** | 0·17 (0·15;0·20) | 1·48 (1·26;1·77) | 0·13 (0·06;0·21) | 8·23 (3·94; 12·64) | 5·45 (4·79;6·45) | 47·25 (41·50;55·88) | 0·0038 (0·0014;0·0061) | 0·30 (0·12;0·48) |
| | **Change (%) 2010-2019** | -8·80 (-14·71;-2·14) | -8·80 (-14·71;-2·14) | -47·88 (-67·49;-17·63) | -45·86 (-63·55;-23·45) | -0·65 (-5·92;5·46) | -0·65 (-5·92;5·46) | -45·99 (-67·12;-15·85) | -41·45 (-64·75;-11·78) |
| **Bulgaria** | **2010** | 0·33 (0·28;0·39) | 2·87 (2·40;3·37) | 0·96 (0·50;2·39) | 42·85 (20·81; 111·67) | 4·81 (4·23;5·46) | 41·67 (36·69;47·32) | 0·0114 (0·0052;0·0310) | 0·70 (0·30;1·97) |
| | **2019** | 0·32 (0·27;0·38) | 2·76 (2·34;3·28) | 0·70 (0·37;1·66) | 29·82 (16·18; 67·92) | 4·87 (4·29;5·60) | 42·17 (37·20;48·51) | 0·0081 (0·0039;0·0199) | 0·48 (0·23;1·12) |
| | **Change (%) 2010-2019** | -3·81 (-14·11;5·64) | -3·81 (-14·11;5·64) | -26·49 (-46·34;11·16) | -30·41 (-51·73;7·62) | 1·22 (-6·92;9·08) | 1·22 (-6·92;9·08) | -29·02 (-51·41;14·11) | -30·96 (-55·43;15·91) |
| **Croatia** | **2010** | 0·18 (0·15;0·21) | 1·55 (1·33;1·81) | 0·09 (0·03;0·13) | 4·95 (2·89; 7·71) | 4·16 (3·68;4·75) | 36·09 (31·92;41·13) | 0·0013 (0·0006;0·0019) | 0·11 (0·07;0·17) |
| | **2019** | 0·17 (0·15;0·20) | 1·51 (1·27;1·76) | 0·05 (0·02;0·09) | 3·91 (2·27; 6·55) | 4·08 (3·58;4·68) | 35·34 (31·07;40·53) | 0·0008 (0·0003;0·0013) | 0·09 (0·05;0·15) |
| | **Change (%) 2010-2019** | -2·62 (-11·48;6·76) | -2·62 (-11·48;6·76) | -38·74 (-59·36;-12·11) | -21·10 (-36·13;-7·09) | -2·06 (-8·98;5·17) | -2·06 (-8·98;5·17) | -38·71 (-59·32;-11·51) | -17·52 (-34·18;1·00) |
| **Czechia** | **2010** | 0·38 (0·33;0·45) | 3·32 (2·82;3·93) | 0·32 (0·09;0·44) | 14·92( 6·93; 21·85) | 3·79 (3·32;4·43) | 32·81 (28·81;38·40) | 0·0021 (0·0006;0·0029) | 0·13 (0·06;0·19) |
| | **2019** | 0·39 (0·33;0·45) | 3·38 (2·90;3·93) | 0·22 (0·07;0·33) | 11·89 (6·06; 17·90) | 3·65 (3·20;4·20) | 31·63 (27·75;36·40) | 0·0013 (0·0004;0·0020) | 0·10 (0·05;0·16) |
| | **Change (%) 2010-2019** | 1·97 (-7·04;11·07) | 1·97 (-7·04;11·07) | -31·69 (-50·14;-7·24) | -20·32 (-35·84;-3·86) | -3·58 (-9·84;3·21) | -3·58 (-9·84;3·21) | -36·71 (-53·03;-16·82) | -20·74 (-34·84;-6·11) |
| **Hungary** | **2010** | 0·51 (0·43;0·60) | 4·39 (3·75;5·18) | 0·11 (0·03;0·16) | 10·30 (5·40; 17·81) | 5·75 (5·04;6·61) | 49·84 (43·72;57·29) | 0·0008 (0·0002;0·0012) | 0·11 (0·06;0·20) |
| | **2019** | 0·45 (0·39;0·54) | 3·94 (3·38;4·66) | 0·12 (0·02;0·20) | 9·71 (4·62; 16·71) | 5·16 (4·54;6·03) | 44·71 (39·32;52·30) | 0·0008 (0·0001;0·0014) | 0·11 (0·05;0·18) |
| | **Change (%) 2010-2019** | -10·26 (-16·58;-3·28) | -10·26 (-16·58;-3·28) | 8·56 (-31·53;51·57) | -5·73 (-18·49;11·69) | -10·29 (-16·10;-4·28) | -10·29 (-16·10;-4·28) | 2·31 (-38·10;43·39) | -6·88 (-19·92;12·65) |

| | | | | | | | | | |
|---|---|---|---|---|---|---|---|---|---|
| **Montenegro** | **2010** | 0·02 (0·02;0·03) | 0·22 (0·19;0·26) | 0·02 (0·01;0·02) | 1.05 (0.72; 1.46) | 4·19 (3·68;4·89) | 36·33 (31·86;42·42) | 0·0024 (0·0016;0·0033) | 0·17 (0·12;0·24) |
| | **2019** | 0·03 (0·02;0·03) | 0·22 (0·19;0·26) | 0·01 (0·01;0·02) | 0.88 (0.56; 1.32) | 4·28 (3·70;4·99) | 37·05 (32·05;43·26) | 0·0019 (0·0011;0·0029) | 0·14 (0·09;0·22) |
| | **Change (%) 2010-2019** | 1·14 (-6·23;8·25) | 1·14 (-6·23;8·25) | -17·17 (-42·88;24·43) | -16·36 (-37·24;10·72) | 2·00 (-4·52;8·44) | 2·00 (-4·52;8·44) | -23·04 (-48·41;17·57) | -17·89 (-39·25;10·30) |
| **North Macedonia** | **2010** | 0·09 (0·08;0·11) | 0·82 (0·70;0·96) | 0·05 (0·02;0·07) | 2·56 (1·72; 3·93) | 4·91 (4·29;5·67) | 42·55 (37·21;49·14) | 0·0025 (0·0010;0·0036) | 0·14 (0·09;0·21) |
| | **2019** | 0·10 (0·08;0·12) | 0·84 (0·71;1·02) | 0·05 (0·02;0·08) | 2·62 (1·67; 4·17) | 4·79 (4·14;5·62) | 41·54 (35·91;48·73) | 0·0023 (0·0008;0·0035) | 0·14 (0·09;0·22) |
| | **Change (%) 2010-2019** | 2·58 (-4·99;10·91) | 2·58 (-4·99;10·91) | 5·54 (-31·22;58·95) | 2·07 (-20·10;29·17) | -2·38 (-8·36;3·90) | -2·38 (-8·36;3·90) | -9·59 (-39·86;30·41) | -3·29 (-28·14;30·17) |
| **Poland** | **2010** | 1·66 (1·41;1·98) | 14·43 (12·25;17·20) | 1·08 (0·63;1·90) | 61·30 (43·53; 95·19) | 5·05 (4·36;5·95) | 43·76 (37·82;51·57) | 0·0023 (0·0015;0·0043) | 0·17 (0·12;0·28) |
| | **2019** | 1·74 (1·47;2·08) | 15·10 (12·77;18·06) | 0·84 (0·46;1·52) | 51·62 (33·09; 80·26) | 4·91 (4·24;5·78) | 42·55 (36·70;50·06) | 0·0016 (0·0010;0·0030) | 0·14 (0·09;0·22) |
| | **Change (%) 2010-2019** | 4·67 (0·69;8·81) | 4·67 (0·69;8·81) | -22·03 (-37·39;-5·07) | -15·78 (-30·17;-3·27) | -2·77 (-4·32;-1·16) | -2·77 (-4·32;-1·16) | -29·86 (-44·80;-12·13) | -19·95 (-37·29;-6·96) |
| **Romania** | **2010** | 1·48 (1·30;1·68) | 12·80 (11·23;14·56) | 0·65 (0·43;1·28) | 50·19 (33·87; 81·69) | 8·13 (7·47;8·87) | 70·49 (64·73;76·85) | 0·0031 (0·0020;0·0059) | 0·29 (0·19;0·48) |
| | **2019** | 1·19 (1·02;1·40) | 10·28 (8·88;12·12) | 0·54 (0·28;0·95) | 40·22 (25·80; 63·28) | 7·08 (6·21;8·10) | 61·39 (53·80;70·17) | 0·0027 (0·0015;0·0047) | 0·26 (0·16;0·41) |
| | **Change (%) 2010-2019** | -19·69 (-26·04;-12·81) | -19·69 (-26·04;-12·81) | -16·80 (-41·74;19·94) | -19·87 (-36·80;1·70) | -12·92 (-20·49;-4·21) | -12·92 (-20·49;-4·21) | -11·18 (-40·31;32·14) | -10·39 (-32·78;19·62) |
| **Serbia** | **2010** | 0·43 (0·37;0·52) | 3·76 (3·21;4·51) | 0·07 (0·04;0·10) | 8·59 (5·24; 14·52) | 5·15 (4·45;6·14) | 44·67 (38·58;53·17) | 0·0006 (0·0004;0·0009) | 0·10 (0·06;0·17) |
| | **2019** | 0·42 (0·35;0·49) | 3·61 (3·06;4·24) | 0·05 (0·03;0·07) | 7·32 (4·33; 12·90) | 4·97 (4·33;5·80) | 43·04 (37·57;50·26) | 0·0004 (0·0002;0·0006) | 0·09 (0·05;0·15) |
| | **Change (%) 2010-2019** | -3·94 (-10·96;3·91) | -3·94 (-10·96;3·91) | -34·98 (-57·80;-2·63) | -14·79 (-28·24;-3·37) | -3·65 (-9·56;2·76) | -3·65 (-9·56;2·76) | -39·35 (-61·35;-5·40) | -15·00 (-30·57;-0·69) |
| **Slovakia** | **2010** | 0·25 (0·21;0·30) | 2·14 (1·84;2·58) | 0·04 (0·02;0·07) | 5·59 (3·55; 9·27) | 5·14 (4·52;6·00) | 44·53 (39·20;52·00) | 0·0008 (0·0005;0·0013) | 0·12 (0·08;0·20) |
| | **2019** | 0·24 (0·21;0·29) | 2·11 (1·81;2·47) | 0·03 (0·01;0·06) | 4·91 (2·88; 8·51) | 4·89 (4·28;5·66) | 42·37 (37·12;49·04) | 0·0006 (0·0003;0·0011) | 0·11 (0·06;0·18) |
| | **Change (%) 2010-2019** | -1·32 (-7·82;5·33) | -1·32 (-7·82;5·33) | -26·19 (-55·82;14·74) | -12·32 (-30·14;6·47) | -4·84 (-10·61;0·57) | -4·84 (-10·61;0·57) | -26·96 (-58·98;28·47) | -14·11 (-35·22;10·99) |
| **Slovenia** | **2010** | 0·10 (0·09;0·12) | 0·89 (0·77;1·04) | 0·04 (0·01;0·06) | 2·59 (1·58; 4·16) | 5·46 (4·79;6·24) | 47·33 (41·50;54·05) | 0·0014 (0·0006;0·0021) | 0·15 (0·09;0·23) |
| | **2019** | 0·11 (0·09;0·13) | 0·92 (0·78;1·09) | 0·03 (0·01;0·05) | 2·19 (1·25; 3·73) | 5·25 (4·58;6·08) | 45·51 (39·74;52·71) | 0·0009 (0·0002;0·0014) | 0·11 (0·06;0·19) |
| | **Change (%) 2010-2019** | 3·07 (-5·70;12·23) | 3·07 (-5·70;12·23) | -20·46 (-51·40;24·03) | -15·46 (-35·08;1·08) | -3·84 (-11·00;2·47) | -3·84 (-11·00;2·47) | -38·66 (-65·50;-3·67) | -25·89 (-46·83;-6·29) |
| **Cirrhosis and other chronic liver diseases due to hepatitis** | | | | | | | | | |

| | | | | | | | | | |
|---|---|---|---|---|---|---|---|---|---|
| **Albania** | **2010** | 27·95 (22·45;34·57) | 0·16 (0·11;0·23) | 54·63 (37·75; 75·96) | 1480·40 (1039·14; 2104·69) | 903·75 (720·71;1118·88) | 4·97 (3·35;7·05) | 1·6251 (1·1353; 2·2248) | 43·58 (31·03;61·04) |
| | **2019** | 27·47 (22·37;33·83) | 0·15 (0·10;0·22) | 64·09 (40·23; 95·24) | 1603·14 (1026·26; 2396·60) | 879·11 (710·92;1090·49) | 5·11 (3·41;7·28) | 1·5785 (1·0197; 2·3051) | 42·39 (27·85;62·20) |
| | **Change (%) 2010-2019** | -1·71 (-8·37;6·03) | -6·55 (-15·80;3·71) | 17·33 (-13·91;53·16) | 8·29 (-20·49;42·43) | -2·73 (-9·33;4·35) | 2·76 (-6·43;13·71) | -2·87 (-27·59;26·10) | -2·73 (-28·01;27·14) |
| **Bosnia and Herzegovina** | **2010** | 44·15 (35·68;54·36) | 0·22 (0·15;0·32) | 108·16 (77·67;150·87) | 3083·11 (2201·43; 4284·82) | 1029·43 (824·21;1265·39) | 4·81 (3·21;6·89) | 1·9702 (1·4276; 2·7154) | 57·33 (41·34;78·45) |
| | **2019** | 40·30 (33·00;50·05) | 0·19 (0·12;0·27) | 111·53 (70·44;164·99) | 3052·10 (1934·07; 4468·73) | 1018·26 (815·67;1263·96) | 4·80 (3·18;6·83) | 1·9636( 1·2656; 2·8462) | 57·05 (37·07;81·59) |
| | **Change (%) 2010-2019** | -8·72 (-14·35;-2·15) | -15·27 (-21·87;-7·95) | 3·12 (-20·83;34·05) | -1·01 (-24·54;29·69) | -1·08 (-7·38;5·87) | -0·15 (-7·76;8·39) | -0·33 (-23·06;29·61) | -0·49 (-23·08;30·00) |
| **Bulgaria** | **2010** | 78·92 (64·64;95·46) | 0·55 (0·35;0·81) | 480·74 (352·67;663·35) | 14817·20 (10794·22; 20303·00) | 892·72 (728·81;1085·22) | 6·62 (4·20;9·78) | 4·0451 (3·0058; 5·5152) | 135·20 (99·79;184·69) |
| | **2019** | 76·50 (62·59;93·58) | 0·54 (0·35;0·78) | 494·49 (318·52;718·08) | 14922·59 (9765·75; 21545·14) | 906·64 (732·50;1116·59) | 6·78 (4·44;9·89) | 4·1933(2·7571; 6·0791) | 140·15 (92·61;200·80) |
| | **Change (%) 2010-2019** | -3·06 (-11·39;5·49) | -2·72 (-12·80;7·85) | 2·86 (-21·48;31·42) | 0·71 (-23·81;28·79) | 1·56 (-7·71;10·97) | 2·43 (-7·77;13·15) | 3·66 (-21·31;33·16) | 3·66 (-21·41;32·15) |
| **Croatia** | **2010** | 39·40 (32·25;47·44) | 0·42 (0·27;0·61) | 249·27 (176·31;344·51) | 7036·17 (4999·09; 9672·74) | 753·78 (611·91;909·78) | 8·33 (5·35;12·20) | 3·4716 (2·4838; 4·7695) | 104·94 (75·70;143·08) |
| | **2019** | 39·13 (32·12;47·80) | 0·35 (0·22;0·51) | 209·19 (134·85;302·60) | 5607·59 (3648·48; 8217·49) | 740·74 (596·75;905·76) | 7·11 (4·58;10·38) | 2·7167 (1·7739; 3·9219) | 80·53 (52·77;116·84) |
| | **Change (%) 2010-2019** | -0·67 (-8·07;7·61) | -18·65 (-26·16;-10·91) | -16·08 (-35·40;8·40) | -20·30 (-39·75;3·75) | -1·73 (-9·30;6·99) | -14·69 (-22·74;-6·76) | -21·74 (-39·75;1·43) | -23·26 (-41·49;0·13) |
| **Czechia** | **2010** | 86·30 (70·23;105·50) | 0·50 (0·31;0·75) | 370·32 (261·52;515·18) | 11461·51 (8063·09; 16075·88) | 693·09 (558·63;852·27) | 4·22 (2·65;6·22) | 2·2877 (1·6301; 3·1468) | 74·84 (53·65;102·77) |
| | **2019** | 86·86 (70·78;105·28) | 0·59 (0·37;0·86) | 423·89 (283·01;602·47) | 12530·05( 8456·88; 17727·65) | 664·81 (533·75;810·95) | 4·63 (2·97;6·76) | 2·4016 (1·6117; 3·4402) | 77·30 (52·45;109·60) |
| | **Change (%) 2010-2019** | 0·64 (-6·26;8·31) | 16·23 (0·26;34·03) | 14·47 (-9·30;45·49) | 9·32 (-13·43;38·94) | -4·08 (-10·82;3·14) | 9·91 (-3·32;24·69) | 4·98 (-16·32;32·89) | 3·28 (-18·00;30·18) |
| **Hungary** | **2010** | 129·56 (105·56;157·93) | 0·74 (0·46;1·08) | 665·54 (461·39;944·61) | 20847·84 (14680·29; 29388·47) | 1118·44 (902·15;1378·20) | 6·68 (4·20;9·69) | 4·3122 (3·0794; 6·0803) | 142·72 (100·85;199·53) |
| | **2019** | 116·96 (94·97;143·82) | 0·64 (0·40;0·94) | 512·54 (337·00;740·77) | 15129·01 (9826·32; 21976·06) | 1003·19 (799·38;1243·64) | 5·34 (3·43;7·77) | 3·1213 (2·0266; 4·5085) | 99·51 (65·43;142·55) |
| | **Change (%) 2010-2019** | -9·73 (-16·36;-2·29) | -13·43 (-26·20;0·57) | -22·99 (-38·97;-4·52) | -27·43 (-42·29;-10·30) | -10·30 (-17·23;-2·62) | -20·08 (-30·28;-9·15) | -27·62 (-42·35;-10·37) | -30·28 (-44·14;-13·90) |
| **Montenegro** | **2010** | 5·52 (4·48;6·84) | 0·01 (0·01;0·02) | 8·10 (5·63; 11·26) | 255·66 (178·11; 357·20) | 784·95 (630·01;975·44) | 1·81 (1·18;2·63) | 0·9547 (0·6728; 1·3167) | 30·95 (21·79;43·08) |
| | **2019** | 5·75 (4·64;7·07) | 0·01 (0·01;0·02) | 9·54 (6·38; 13·91) | 292·16 (193·07; 426·45) | 803·61 (641·74;1001·22) | 2·16 (1·40;3·17) | 1·0382 (0·6975; 1·4860) | 33·41 (22·50;48·75) |
| | **Change (%) 2010-2019** | 4·15 (-2·35;11·25) | 18·13 (4·75;34·48) | 17·88 (-6·67;49·83) | 14·28 (-9·81;45·46) | 2·38 (-4·38;9·70) | 19·18 (6·09;35·53) | 8·75 (-13·49;37·10) | 7·93 (-14·79;36·41) |
| **North** | **2010** | 21·06 | 0·10 | 48·94 | 1439·86 | 898·05 | 3·82 | 1·7924 | 51·76 |

| | | | | | | | | | |
|---|---|---|---|---|---|---|---|---|---|
| **Macedonia** | | (16·84;25·99) | (0·06;0·14) | (34·41; 68·26) | (1020·77; 1982·25) | (716·75;1114·60) | (2·51;5·60) | (1·3013; 2·4630) | (37·43;70·07) |
| | **2019** | 21·56 (17·29;26·95) | 0·12 (0·08;0·17) | 61·48 (38·55; 90·60) | 1762·30 (1140·96; 2603·55) | 869·41 (688·88;1091·54) | 4·43 (2·92;6·35) | 1·9520 (1·2735; 2·8303) | 56·47 (37·27;82·43) |
| | **Change (%) 2010-2019** | 2·39 (-4·74;9·04) | 19·83 (6·83;33·65) | 25·62 (-5·70;63·09) | 22·39 (-8·41;60·41) | -3·19 (-9·98;3·17) | 16·07 (3·59;29·26) | 8·90 (-17·44;40·60) | 9·11 (-17·86;42·09) |
| **Poland** | **2010** | 439·77 (352·19;546·22) | 2·11 (1·47;2·88) | 1712·76 (1463·24;2000·98) | 56608·07 (48280·68; 66344·46) | 1005·24 (802·05;1251·86) | 5·10 (3·55;6·96) | 3·1539 (2·7162; 3·6794) | 108·80 (93·22;126·92) |
| | **2019** | 432·58 (348·97;537·08) | 1·81 (1·17;2·59) | 1583·17 (1235·20;2026·68) | 50682·86 (39261·71; 65305·11) | 941·67 (749·44;1169·48) | 4·10 (2·72;5·73) | 2·6784 (2·0822; 3·4246) | 92·57 (71·55;118·75) |
| | **Change (%) 2010-2019** | -1·63 (-4·04;0·85) | -14·30 (-24·16;-6·26) | -7·57 (-24·05;12·68) | -10·47 (-26·20;9·08) | -6·32 (-8·10;-4·70) | -19·56 (-27·49;-13·85) | -15·08 (-30·02;2·89) | -14·92 (-29·70;3·16) |
| **Romania** | **2010** | 381·43 (326·48;445·79) | 3·02 (1·95;4·37) | 2166·57 (1563·69;2974·48) | 64025·70 (46360·10; 87864·71) | 1609·51 (1365·60;1894·66) | 12·53 (8·13;18·02) | 6·9446 (5·0340; 9·4933) | 218·25 (158·68;295·93) |
| | **2019** | 318·79 (259·83;390·87) | 2·74 (1·73;4·00) | 1901·77 (1285·22;2743·12) | 53843·65 (36401·33; 77323·45) | 1424·94 (1137·46;1771·45) | 11·33 (7·32;16·25) | 5·8177 (3·9641; 8·3397) | 179·60 (122·52;257·67) |
| | **Change (%) 2010-2019** | -16·42 (-25·38;-6·67) | -9·14 (-21·13;2·67) | -12·22 (-28·41;7·67) | -15·90 (-32·02;3·24) | -11·47 (-22·13;-0·54) | -9·56 (-19·02;0·17) | -16·23 (-31·86;2·55) | -17·71 (-33·66;1·39) |
| **Serbia** | **2010** | 101·26 (80·95;125·98) | 0·37 (0·24;0·53) | 249·85 (178·36;355·22) | 7101·59 (5024·88; 9985·34) | 979·64 (780·11;1213·13) | 3·73 (2·45;5·39) | 1·8125 (1·3299; 2·5321) | 54·26 (39·10;74·95) |
| | **2019** | 96·86 (78·94;120·00) | 0·38 (0·25;0·54) | 269·84 (177·39;392·45) | 7450·72 (4920·33; 11019·83) | 939·27 (754·39;1170·18) | 3·95 (2·60;5·64) | 1·8905 (1·2544; 2·7218) | 56·67 (37·77;82·65) |
| | **Change (%) 2010-2019** | -4·34 (-10·79;3·29) | 2·50 (-8·76;14·44) | 8·00 (-17·87;42·29) | 4·92 (-20·75;39·80) | -4·12 (-10·77;4·06) | 5·77 (-5·19;18·26) | 4·30 (-20·29;38·19) | 4·43 (-20·71;39·83) |
| **Slovakia** | **2010** | 59·45 (48·35;72·84) | 0·50 (0·32;0·72) | 346·79 (246·21;477·27) | 11414·72 (8085·48; 15678·58) | 972·70 (785·39;1189·71) | 8·10 (5·11;11·71) | 4·6067 (3·3277; 6·2718) | 155·05 (111·20;211·24) |
| | **2019** | 58·73 (47·34;72·00) | 0·47 (0·30;0·69) | 322·50 (210·23;477·23) | 10180·13 (6624·35; 15035·50) | 923·80 (742·16;1136·88) | 7·09 (4·53;10·38) | 3·8295 (2·5192; 5·6070) | 127·47 (83·39;187·56) |
| | **Change (%) 2010-2019** | -1·21 (-8·74;5·63) | -6·36 (-16·15;4·68) | -7·00 (-29·88;22·29) | -10·82 (-32·72;18·92) | -5·03 (-11·91;1·61) | -12·52 (-20·90;-2·88) | -16·87 (-36·81;9·57) | -17·79 (-37·65;9·45) |
| **Slovenia** | **2010** | 25·04 (20·40;30·31) | 0·21 (0·14;0·31) | 124·68 (89·72;171·80) | 3635·89 (2622·37; 5030·38) | 1039·23 (839·66;1275·30) | 8·46 (5·40;12·14) | 3·8029 (2·7447; 5·2599) | 117·14 (84·96;161·17) |
| | **2019** | 25·31 (20·63;31·28) | 0·17 (0·11;0·25) | 108·08 (69·50;161·02) | 2966·25 (1914·29; 4440·61) | 996·68 (801·09;1225·73) | 6·92 (4·47;10·20) | 2·8894 (1·8872; 4·3298) | 87·21 (57·15;128·58) |
| | **Change (%) 2010-2019** | 1·06 (-6·89;8·29) | -19·52 (-27·46;-11·38) | -13·32 (-33·87;12·62) | -18·42 (-38·59;7·29) | -4·09 (-11·30;3·17) | -18·11 (-26·68;-9·94) | -24·02 (-42·45;-0·29) | -25·55 (-44·17;-1·91) |
| **Liver cancer due to hepatitis** | | | | | | | | | |
| **Albania** | **2010** | 0·05 (0·03;0·07) | 0·05 (0·03;0·07) | 55·93 (38·26; 78·66) | 1144·66 (765·26; 1635·60) | 1·48 (1·01;2·09) | 1·51 (1·03;2·10) | 1·6576 (1·1563;2·3003) | 32·52 (22·16;45·96) |
| | **2019** | 0·07 (0·05;0·11) | 0·07 (0·05;0·11) | 80·62 (51·11;119·92) | 1566·83 (977·06; 2348·42) | 1·68 (1·06;2·51) | 1·69 (1·08;2·52) | 1·8473 (1·1928;2·7266) | 36·02 (22·59;54·01) |
| | **Change (%) 2010-2019** | 43·09 (4·87;87·39) | 43·55 (6·23;87·09) | 44·14 (7·26;87·46) | 36·88 (0·06;80·16) | 13·88 (-16·65;48·66) | 12·36 (-16·79;45·45) | 11·44 (-16·85;44·57) | 10·79 (-19·23;45·62) |
| **Bosnia and** | **2010** | 0·13 | 0·13 | 145·68 | 2953·02 | 2·34 | 2·43 | 2·6756 | 52·11 |

| | | | | | | | | | |
|---|---|---|---|---|---|---|---|---|---|
| **Herzegovina** | | (0·09;0·18) | (0·09;0·18) | (102·94;194·98) | (2049·27; 3973·14) | (1·65;3·11) | (1·74;3·21) | (1·9379;3·5146) | (36·90;69·39) |
| | **2019** | 0·13 (0·09;0·19) | 0·14 (0·09;0·19) | 150·46 (103·11;214·02) | 2873·24 (1881·75; 4128·75) | 2·18 (1·45;3·08) | 2·26 (1·55;3·18) | 2·5017 (1·7352;3·5036) | 47·44 (31·33;67·96) |
| | **Change (%) 2010-2019** | 0·81 (-21·99;28·71) | 1·94 (-20·70;29·67) | 3·28 (-18·98;31·49) | -2·70 (-24·83;24·42) | -6·81 (-27·08;18·04) | -6·91 (-26·80;17·94) | -6·50 (-26·38;18·63) | -8·96 (-29·44;15·60) |
| **Bulgaria** | **2010** | 0·18 (0·12;0·24) | 0·18 (0·13;0·24) | 199·31 (138·86;265·75) | 4064·13 (2780·57; 5492·51) | 1·28 (0·88;1·71) | 1·29 (0·91;1·73) | 1·4015 (0·9942;1·8583) | 29·86 (20·73;40·44) |
| | **2019** | 0·14 (0·09;0·20) | 0·14 (0·09;0·21) | 155·31 (102·76;226·55) | 3062·78 (1975·99; 4522·76) | 0·97 (0·63;1·41) | 0·97 (0·64;1·41) | 1·0475 (0·6948;1·5144) | 22·13 (14·38;32·60) |
| | **Change (%) 2010-2019** | -22·38 (-39·53;-2·86) | -22·39 (-38·88;-3·57) | -22·08 (-38·47;-3·76) | -24·64 (-41·52;-5·40) | -24·24 (-41·14;-4·34) | -25·04 (-41·24;-6·88) | -25·26 (-41·03;-7·39) | -25·88 (-42·37;-6·66) |
| **Croatia** | **2010** | 0·07 (0·05;0·10) | 0·07 (0·04;0·09) | 66·18 (45·20; 90·00) | 1233·37 (834·55; 1704·73) | 0·90 (0·62;1·22) | 0·81 (0·56;1·09) | 0·8173 (0·5626;1·1008) | 15·56 (10·67;21·61) |
| | **2019** | 0·07 (0·04;0·10) | 0·06 (0·04;0·09) | 61·86 (39·52; 89·08) | 1112·49 (701·86; 1649·14) | 0·77 (0·49;1·12) | 0·67 (0·43;0·96) | 0·6657 (0·4339;0·9568) | 12·77 (7·98;19·04) |
| | **Change (%) 2010-2019** | -5·09 (-26·68;22·50) | -6·42 (-27·21;18·48) | -6·53 (-27·25;18·32) | -9·80 (-30·19;15·72) | -14·94 (-34·38;10·24) | -17·49 (-35·93;5·70) | -18·55 (-36·39;3·19) | -17·95 (-36·64;6·18) |
| **Czechia** | **2010** | 0·15 (0·10;0·20) | 0·14 (0·10;0·19) | 148·42 (102·80;200·57) | 2821·05 (1885·71; 3865·62) | 0·80 (0·55;1·08) | 0·77 (0·53;1·04) | 0·8084 (0·5648;1·0819) | 15·67 (10·69;21·24) |
| | **2019** | 0·14 (0·09;0·21) | 0·14 (0·09;0·21) | 150·76 (100·61;215·62) | 2771·70 (1763·67; 4113·39) | 0·68 (0·44;0·99) | 0·65 (0·42;0·94) | 0·6868 (0·4542;0·9827) | 13·33 (8·64;19·49) |
| | **Change (%) 2010-2019** | -0·78 (-19·91;24·41) | 0·32 (-19·10;25·66) | 1·58 (-17·84;26·03) | -1·75 (-22·15;23·85) | -14·83 (-31·66;5·74) | -15·14 (-31·35;4·93) | -15·05 (-31·09;4·20) | -14·95 (-32·47;5·83) |
| **Hungary** | **2010** | 0·18 (0·13;0·24) | 0·18 (0·13;0·24) | 198·78 (142·35;260·47) | 3922·73 (2701·57; 5242·88) | 1·03 (0·72;1·37) | 1·03 (0·73;1·35) | 1·1042 (0·7953;1·4424) | 22·71 (15·80;30·33) |
| | **2019** | 0·12 (0·08;0·17) | 0·12 (0·08;0·17) | 130·85 (87·56;183·81) | 2354·11 (1499·07; 3392·72) | 0·60 (0·38;0·86) | 0·59 (0·39;0·84) | 0·6434 (0·4285;0·8983) | 12·37 (7·92;17·96) |
| | **Change (%) 2010-2019** | -35·81 (-48·82;-20·89) | -35·18 (-47·88;-20·44) | -34·17 (-46·91;-19·37) | -39·99 (-52·25;-25·29) | -42·11 (-53·78;-28·28) | -42·15 (-53·38;-28·88) | -41·73 (-52·99;-28·56) | -45·54 (-56·44;-31·97) |
| **Montenegro** | **2010** | 0·01 (0·01;0·02) | 0·01 (0·01;0·02) | 15·14 (10·50; 20·37) | 314·13 (216·65; 427·84) | 1·59 (1·12;2·14) | 1·59 (1·12;2·12) | 1·7213 (1·2195;2·2764) | 35·06 (24·49;47·28) |
| | **2019** | 0·01 (0·01;0·02) | 0·01 (0·01;0·02) | 15·31 (10·44; 21·82) | 313·41 (203·02; 453·95) | 1·48 (0·99;2·10) | 1·46 (0·99;2·05) | 1·5550 (1·0667;2·1823) | 31·65 (20·82;45·25) |
| | **Change (%) 2010-2019** | 3·41 (-16·81;27·75) | 1·95 (-18·39;25·94) | 1·10 (-19·02;25·17) | -0·23 (-19·82;23·17) | -6·99 (-25·55;14·30) | -8·64 (-26·74;12·09) | -9·66 (-27·29;11·29) | -9·71 (-27·72;10·79) |
| **North Macedonia** | **2010** | 0·07 (0·05;0·09) | 0·07 (0·05;0·09) | 70·56 (49·36; 94·45) | 1507·84 (1017·71; 2055·69) | 2·42 (1·70;3·23) | 2·49 (1·77;3·27) | 2·7395 (1·9738;3·5507) | 54·13 (37·64;72·78) |
| | **2019** | 0·07 (0·05;0·11) | 0·07 (0·05;0·11) | 77·64 (50·74;111·82) | 1631·02 (1035·19; 2390·40) | 2·28 (1·51;3·24) | 2·32 (1·56;3·27) | 2·5466 (1·7286;3·5521) | 49·98 (32·70;72·05) |
| | **Change (%) 2010-2019** | 10·82 (-13·02;42·40) | 10·00 (-13·90;40·45) | 10·04 (-13·39;39·66) | 8·17 (-15·82;39·23) | -5·87 (-25·81;19·83) | -6·88 (-26·08;17·33) | -7·04 (-25·46;16·37) | -7·67 (-27·73;17·98) |
| **Poland** | **2010** | 0·25 (0·22;0·29) | 0·27 (0·23;0·30) | 297·98 (257·75;338·71) | 5481·60 (4709·82; 6307·32) | 0·42 (0·36;0·48) | 0·44 (0·38;0·50) | 0·4924 (0·4262;0·5589) | 9·16 (7·87;10·53) |

| | | | | | | | | | |
|---|---|---|---|---|---|---|---|---|---|
| | **2019** | 0·31 (0·25;0·39) | 0·33 (0·27;0·41) | 376·19 (296·74;460·26) | 6657·24 (5241·23; 8147·88) | 0·44 (0·35;0·54) | 0·46 (0·37;0·56) | 0·5115 (0·4051;0·6248) | 9·45 (7·47;11·56) |
| | **Change (%) 2010-2019** | 25·52 (7·67;46·59) | 25·51 (8·00;46·37) | 26·25 (7·37;46·05) | 21·45 (1·37;41·57) | 5·70 (-9·49;24·75) | 4·33 (-10·44;22·42) | 3·87 (-11·68;20·30) | 3·21 (-13·65;20·86) |
| **Romania** | **2010** | 0·22 (0·15;0·29) | 0·22 (0·15;0·30) | 241·00 (167·41;323·06) | 4959·37 (3464·55; 6689·33) | 0·64 (0·45;0·86) | 0·64 (0·46;0·86) | 0·6951 (0·4961;0·9162) | 14·74 (10·28;19·98) |
| | **2019** | 0·26 (0·17;0·36) | 0·26 (0·17;0·37) | 283·76 (192·93;393·65) | 5574·84(3620·48; 7901·32) | 0·70 (0·45;0·98) | 0·69 (0·46;0·97) | 0·7394 (0·5023;1·0323) | 15·59 (9·99;22·10) |
| | **Change (%) 2010-2019** | 17·29 (-4·01;43·21) | 17·17 (-3·86;42·15) | 17·75 (-2·88;42·49) | 12·41 (-8·10;38·54) | 9·29 (-10·59;33·88) | 7·35 (-12·16;30·91) | 6·37 (-12·76;29·76) | 5·79 (-14·01;30·82) |
| **Serbia** | **2010** | 0·30 (0·22;0·39) | 0·30 (0·21;0·39) | 315·21 (226·67;412·05) | 6359·44 (4482·17; 8314·80) | 2·00 (1·44;2·60) | 1·97 (1·44;2·54) | 2·0979 (1·5319;2·7083) | 42·32 (30·26;55·15) |
| | **2019** | 0·30 (0·20;0·42) | 0·29 (0·19;0·41) | 304·99 (209·45;426·82) | 5930·60 (3936·77; 8402·04) | 1·83 (1·23;2·57) | 1·76 (1·22;2·43) | 1·8668 (1·2965;2·5604) | 37·09 (24·75;52·77) |
| | **Change (%) 2010-2019** | -2·29 (-23·86;25·38) | -3·23 (-24·41;23·14) | -3·24 (-24·45;22·49) | -6·74 (-27·67;19·86) | -8·55 (-29·10;17·56) | -10·50 (-29·78;13·84) | -11·02 (-30·17;11·98) | -12·34 (-31·99;12·77) |
| **Slovakia** | **2010** | 0·08 (0·05;0·10) | 0·07 (0·05;0·10) | 76·32 (53·70; 99·53) | 1537·78 (1062·59; 2080·01) | 0·97 (0·68;1·30) | 0·93 (0·66;1·22) | 0·9847 (0·6986;1·2825) | 19·77 (13·82;26·62) |
| | **2019** | 0·08 (0·05;0·11) | 0·07 (0·05;0·11) | 76·85 (49·73;110·60) | 1527·47 (974·41; 2283·61) | 0·83 (0·54;1·22) | 0·79 (0·51;1·14) | 0·8247 (0·5420;1·1777) | 16·53 (10·60;24·48) |
| | **Change (%) 2010-2019** | 2·30 (-21·25;30·70) | 1·02 (-21·45;28·61) | 0·70 (-21·57;27·97) | -0·67 (-23·50;27·99) | -14·09 (-33·10;9·67) | -15·73 (-34·57;6·99) | -16·25 (-34·72;5·84) | -16·34 (-35·50;7·13) |
| **Slovenia** | **2010** | 0·05 (0·04;0·07) | 0·05 (0·03;0·07) | 50·36 (35·80; 66·20) | 952·02 (653·59; 1283·97) | 1·47 (1·03;1·97) | 1·32 (0·94;1·76) | 1·3459 (0·9573;1·7714) | 26·65 (18·48;35·99) |
| | **2019** | 0·07 (0·04;0·10) | 0·06 (0·04;0·09) | 61·35 (40·02; 86·55) | 1104·48 (698·35; 1617·96) | 1·54 (1·00;2·24) | 1·33 (0·86;1·92) | 1·3337 (0·8580;1·9005) | 26·14 (16·27;38·90) |
| | **Change (%) 2010-2019** | 25·55 (-3·87;63·02) | 22·62 (-5·92;57·21) | 21·84 (-6·79;56·12) | 16·01 (-12·95;51·59) | 4·36 (-20·79;36·73) | 0·75 (-23·22;30·36) | -0·91 (-24·12;28·01) | -1·94 (-26·78;29·61) |

UI= Uncertainty Interval; A:

**Supplement Table H·** Prevalence, incidence, mortality and DALYs attributable to HCV in 2010 and 2019, by Eastern Europe countries·

| | | N in thousands (95%UI) | | N (95%UI) | | Age- standardized rate x 100,000 (95%UI) | | | |
|---|---|---|---|---|---|---|---|---|---|
| | **Year** | **Prevalence** | **Incidence** | **Mortality** | **DALYs** | **Prevalence** | **Incidence** | **Mortality** | **DALYs** |
| | | | | **Acute hepatitis** | | | | | |
| **Belarus** | **2010** | 0·77 (0·67;0·90) | 6·65 (5·80;7·81) | 1·39 (0·67;2·44) | 80·70 (44·65; 145·35) | 9·69 (8·46;11·22) | 84·00 (73·29;97·21) | 0·0146 (0·0073;0·0275) | 1·00 (0·54;1·90) |
| | **2019** | 0·74 (0·64;0·88) | 6·41 (5·57;7·59) | 0·80 (0·31;1·58) | 49·99 (24·34; 97·54) | 9·07 (7·90;10·60) | 78·64 (68·50;91·83) | 0·0081 (0·0031;0·0171) | 0·60 (0·28;1·26) |
| | **Change (%) 2010-2019** | -3·59 (-9·33;2·89) | -3·59 (-9·33;2·89) | -42·08 (-72·66;-3·18) | -38·05 (-66·55;-0·41) | -6·38 (-12·23;-0·49) | -6·38 (-12·23;-0·49) | -44·80 (-74·42;-1·00) | -40·30 (-68·87;1·79) |
| **Estonia** | **2010** | 0·10 (0·09;0·12) | 0·87 (0·75;1·00) | 0·02 (0·01;0·04) | 2·23 (1·36; 3·72) | 8·64 (7·51;10·03) | 74·84 (65·12;86·89) | 0·0014 (0·0006;0·0021) | 0·19 (0·11;0·32) |
| | **2019** | 0·10 (0·08;0·11) | 0·84 (0·73;0·99) | 0·02 (0·01;0·03) | 1·94 (1·12; 3·41) | 8·38 (7·34;9·83) | 72·67 (63·59;85·18) | 0·0010 (0·0003;0·0016) | 0·16 (0·09;0·29) |
| | **Change (%) 2010-2019** | -3·10 (-8·86;3·31) | -3·10 (-8·86;3·31) | -12·96 (-53·31;38·93) | -13·01 (-30·40;1·47) | -2·90 (-8·68;2·51) | -2·90 (-8·68;2·51) | -25·71 (-59·67;13·16) | -14·50 (-31·25;0·74) |
| **Latvia** | **2010** | 0·14 (0·12;0·16) | 1·21 (1·05;1·41) | 0·03 (0·01;0·05) | 3·29 (1·82; 5·28) | 7·73 (6·74;8·95) | 67·01 (58·45;77·59) | 0·0015 (0·0004;0·0022) | 0·19 (0·11;0·31) |
| | **2019** | 0·13 (0·11;0·15) | 1·12 (0·96;1·32) | 0·02 (0·00;0·04) | 2·69 (1·31; 4·49) | 7·57 (6·60;8·82) | 65·58 (57·22;76·47) | 0·0010 (0·0002;0·0018) | 0·16 (0·08;0·27) |
| | **Change (%) 2010-2019** | -6·86 (-13·82;-0·74) | -6·86 (-13·82;-0·74) | -31·11 (-73·48;9·64) | -18·24 (-39·55;1·24) | -2·13 (-8·18;3·53) | -2·13 (-8·18;3·53) | -34·60 (-76·66;11·25) | -17·65 (-44·56;8·86) |
| **Lithuania** | **2010** | 0·25 (0·22;0·30) | 2·18 (1·91;2·56) | 0·05 (0·01;0·08) | 4·99 (2·86; 8·68) | 10·06 (8·88;11·60) | 87·23 (76·94;100·57) | 0·0012 (0·0003;0·0018) | 0·19 (0·11;0·33) |
| | **2019** | 0·22 (0·19;0·26) | 1·94 (1·67;2·25) | 0·04 (0·01;0·07) | 4·04 (2·22; 7·28) | 9·62 (8·39;11·20) | 83·42 (72·69;97·10) | 0·0008 (0·0002;0·0014) | 0·17 (0·09;0·30) |
| | **Change (%) 2010-2019** | -10·93 (-16·87;-4·46) | -10·93 (-16·87;-4·46) | -26·82 (-60·46;14·19) | -19·15 (-31·00;-7·99) | -4·37 (-10·83;2·53) | -4·37 (-10·83;2·53) | -33·79 (-61·36;-1·06) | -12·99 (-27·42;0·93) |
| **Republic of Moldova** | **2010** | 0·41 (0·35;0·49) | 3·55 (3·04;4·21) | 0·18 (0·07;0·24) | 13·69 (7·80; 20·81) | 12·93 (11·27;14·95) | 112·03 (97·65;129·58) | 0·0042 (0·0018;0·0056) | 0·38 (0·23;0·59) |
| | **2019** | 0·34 (0·29;0·40) | 2·95 (2·53;3·47) | 0·09( 0·03;0·13) | 8·43 (4·62; 13·54) | 11·46 (9·95;13·24) | 99·32 (86·27;114·78) | 0·0020 (0·0007;0·0030) | 0·26 (0·15;0·43) |
| | **Change (%) 2010-2019** | -16·75 (-22·07;-11·34) | -16·75 (-22·07;-11·34) | -51·98 (-70·07;-32·21) | -38·44 (-51·46;-25·63) | -11·34 (-16·77;-6·19) | -11·34 (-16·77;-6·19) | -51·72 (-70·46;-32·17) | -32·17 (-47·26;-17·88) |
| **Russian Federation** | **2010** | 11·74 (10·04;13·93) | 101·71 (87·01;120·72) | 3·09 (1·70;5·45) | 328·32 (213·36; 545·87) | 9·47 (8·17;11·14) | 82·11 (70·77;96·58) | 0·0022 (0·0014;0·0045) | 0·27 (0·17;0·48) |
| | **2019** | 12·19 (10·43;14·49) | 105·65 (90·40;125·56) | 1·94 (0·88;3·25) | 269·05 (166·92; 446·40) | 9·36 (8·10;11·04) | 81·15 (70·22;95·66) | 0·0013 (0·0007;0·0026) | 0·21 (0·13;0·36) |
| | **Change (%) 2010-2019** | 3·88 (0·15;7·35) | 3·88 (0·15;7·35) | -37·18 (-55·10;-17·44) | -18·05 (-35·81;-5·23) | -1·17 (-1·93;-0·37) | -1·17 (-1·93;-0·37) | -38·67 (-55·46;-18·69) | -20·48 (-37·85;-7·72) |

| | | | | | | | | | |
|---|---|---|---|---|---|---|---|---|---|
| **Ukraine** | **2010** | 4·08 (3·48;4·86) | 35·39 (30·14;42·12) | 19·93 (8·88;28·40) | 941·71 (488·60;1351·49) | 10·95 (9·46;12·99) | 94·90 (81·95;112·59) | 0·0376 (0·0192;0·0555) | 2·02 (1·24;3·14) |
| | **2019** | 3·97 (3·38;4·74) | 34·38 (29·31;41·04) | 10·79 (5·33;18·66) | 547·65(302·28; 938·75) | 11·20 (9·61;13·18) | 97·07 (83·26;114·24) | 0·0213 (0·0111;0·0386) | 1·26 (0·71;2·27) |
| | **Change (%) 2010-2019** | -2·86 (-7·33;1·15) | -2·86 (-7·33;1·15) | -45·87 (-70·58;-14·68) | -41·85 (-67·14;-12·09) | 2·30 (-1·33;6·02) | 2·30 (-1·33;6·02) | -43·17 (-70·11;-12·72) | -37·65 (-64·38;-10·03) |
| **Cirrhosis and other chronic liver diseases due to hepatitis** | | | | | | | | | |
| **Belarus** | **2010** | 223·44 (179·29;272·85) | 0·49 (0·30;0·73) | 570·56 (407·80;794·80) | 20581·17 (14715·11; 28525·88) | 2043·88 (1620·67;2507·68) | 4·76 (2·93;7·20) | 4·3614 (3·1639; 6·0300) | 163·62 (118·91;225·73) |
| | **2019** | 206·91 (168·82;253·62) | 0·44 (0·26;0·67) | 396·94 (256·88;593·87) | 13580·71 (8632·55; 20051·64) | 1899·83 (1543·50;2341·28) | 4·30 (2·60;6·51) | 2·8163 (1·8311; 4·1323) | 103·04 (66·24;152·28) |
| | **Change (%) 2010-2019** | -7·40 (-14·11;-0·59) | -10·62 (-22·02;1·51) | -30·43 (-47·11;-8·69) | -34·01 (-49·75;-12·59) | -7·05 (-13·88;-0·11) | -9·77 (-20·49;1·48) | -35·43 (-50·49;-15·42) | -37·02 (-51·87;-17·05) |
| **Estonia** | **2010** | 27·49 (22·20;33·33) | 0·05 (0·03;0·07) | 46·94 (33·43; 65·40) | 1588·57 (1134·37; 2213·61) | 1780·25 (1422·38;2172·14) | 3·38 (2·02;5·13) | 2·4684 (1·7499; 3·4266) | 89·49 (64·31;124·08) |
| | **2019** | 26·88 (21·92;32·93) | 0·05 (0·03;0·07) | 48·49 (31·18; 73·11) | 1615·13 (1023·66; 2376·20) | 1727·11 (1397·92;2152·51) | 3·48 (2·03;5·27) | 2·5001 (1·5860; 3·7116) | 90·94 (58·22;133·55) |
| | **Change (%) 2010-2019** | -2·23 (-8·27;3·81) | 1·72 (-10·49;14·40) | 3·32 (-21·39;33·48) | 1·67 (-22·82;31·10) | -2·98 (-9·42;2·86) | 3·10 (-8·99;16·14) | 1·29 (-22·87;30·70) | 1·62 (-22·96;30·72) |
| **Latvia** | **2010** | 38·64 (31·70;46·77) | 0·08 (0·05;0·12) | 87·18 (63·16;119·84) | 3007·49 (2160·95; 4140·13) | 1565·14 (1270·46;1897·04) | 3·70 (2·26;5·66) | 2·9025 (2·0893; 4·0138) | 108·05 (77·18;147·54) |
| | **2019** | 35·19 (28·85;43·03) | 0·06 (0·04;0·09) | 67·82 (45·79; 98·93) | 2250·27 (1487·23; 3254·07) | 1530·45 (1227·50;1883·49) | 3·20 (1·93;4·87) | 2·3386 (1·5509; 3·3849) | 86·35 (56·94;124·65) |
| | **Change (%) 2010-2019** | -8·92 (-14·99;-3·33) | -23·54 (-32·75;-12·90) | -22·20 (-37·65;-2·31) | -25·18 (-40·11;-6·60) | -2·22 (-8·78;4·42) | -13·45 (-23·96;-1·80) | -19·43 (-35·22;0·32) | -20·08 (-35·97;-0·53) |
| **Lithuania** | **2010** | 74·99 (61·84;91·80) | 0·16 (0·10;0·25) | 211·89 (151·70;293·11) | 7654·50 (5495·55; 10582·51) | 2125·54 (1735·92;2607·99) | 5·45 (3·24;8·35) | 5·0161 (3·6209; 6·8861) | 192·22 (139·21;265·11) |
| | **2019** | 66·77 (54·58;81·88) | 0·12 (0·07;0·18) | 147·23 (96·72;212·39) | 5094·46 (3327·26; 7328·15) | 2026·02 (1636·20;2509·72) | 4·57 (2·71;7·00) | 3·5994 (2·3574; 5·1540) | 137·52 (88·80;198·37) |
| | **Change (%) 2010-2019** | -10·96 (-17·19;-3·80) | -27·67 (-37·40;-18·21) | -30·52 (-44·62;-12·88) | -33·44 (-46·79;-16·75) | -4·68 (-11·78;3·37) | -16·21 (-27·99;-4·55) | -28·24 (-42·33;-10·83) | -28·46 (-42·65;-11·16) |
| **Republic of Moldova** | **2010** | 116·40 (93·97;142·24) | 0·78 (0·47;1·17) | 629·55 (449·03;890·58) | 20200·07 (14248·79; 28411·21) | 2726·67 (2187·55;3352·31) | 16·91 (10·37;25·42) | 12·2685 (8·8177;17·1401) | 398·44 (284·10;553·99) |
| | **2019** | 100·93 (81·55;124·07) | 0·59 (0·36;0·90) | 439·70 (297·19;635·60) | 13561·61 (9101·67; 19330·46) | 2394·85 (1907·83;2953·05) | 13·23 (8·10;20·24) | 7·9341 (5·4585;11·2456) | 254·55 (174·02;355·71) |
| | **Change (%) 2010-2019** | -13·29 (-19·88;-6·98) | -24·51 (-32·91;-13·80) | -30·16 (-40·42;-18·28) | -32·86 (-42·83;-21·32) | -12·17 (-18·86;-5·67) | -21·78 (-29·64;-12·21) | -35·33 (-44·49;-24·67) | -36·11 (-45·49;-24·95) |
| **Russian Federation** | **2010** | 3188·69 (2569·70;3948·99) | 6·54 (4·08;9·66) | 9090·36 (7738·49;10652·47) | 346063·32 (293248·56; 404134·97) | 1934·69 (1555·30;2398·06) | 4·16 (2·58;6·18) | 4·7503 (4·0614; 5·5527) | 187·88 (159·38;221·16) |
| | **2019** | 3209·77 (2585·30;3956·41) | 7·47 (4·45;11·21) | 8434·54 (6720·10;10370·81) | 309091·54 (246496·71; 379544·72) | 1906·02 (1532·45;2358·18) | 4·64 (2·85;6·85) | 4·1417 (3·3268; 5·0662) | 160·77 (129·09;197·88) |
| | **Change (%) 2010-2019** | 0·66 (-0·45;1·87) | 14·27 (-0·03;26·30) | -7·21 (-21·49;8·57) | -10·68 (-24·35;4·59) | -1·48 (-2·39;-0·44) | 11·62 (2·29;19·88) | -12·81 (-25·79;1·53) | -14·43 (-27·33;-0·48) |

| | | | | | | | | | |
|---|---|---|---|---|---|---|---|---|---|
| **Ukraine** | **2010** | 1203·29<br>(971·01;1487·85) | 2·19<br>(1·26;3·30) | 3226·13<br>(2721·58;3820·06) | 123929·38<br>(104636·41; 146318·77) | 2277·26<br>(1825·35;2809·21) | 4·53<br>(2·55;6·79) | 5·2267<br>(4·4356; 6·1627) | 210·60<br>(176·82;248·77) |
| | **2019** | 1189·65<br>(958·38;1471·03) | 2·27<br>(1·12;3·65) | 3713·92<br>(2877·62;4681·66) | 144961·93<br>(111782·73; 182435·75) | 2332·52<br>(1868·93;2889·33) | 4·86<br>(2·50;7·65) | 6·0935<br>(4·7202; 7·6708) | 251·62<br>(193·30;317·41) |
| | **Change (%) 2010-2019** | -1·13<br>(-5·48;2·87) | 3·73<br>(-17·19;22·09) | 15·12<br>(-6·45;39·77) | 16·97<br>(-5·64;42·79) | 2·43<br>(-2·37;6·85) | 7·14<br>(-11·04;23·47) | 16·59<br>(-5·62;41·50) | 19·48<br>(-3·40;45·24) |
| **Liver cancer due to hepatitis** | | | | | | | | | |
| **Belarus** | **2010** | 0·10<br>(0·07;0·13) | 0·10<br>(0·07;0·13) | 107·36<br>(75·58;140·52) | 2230·57<br>(1556·68; 2982·93) | 0·68<br>(0·47;0·91) | 0·68<br>(0·48;0·89) | 0·7219<br>(0·5174;0·9444) | 15·35<br>(10·64;20·65) |
| | **2019** | 0·10<br>(0·06;0·15) | 0·09<br>(0·06;0·14) | 99·42<br>(64·50;142·04) | 1974·44<br>(1236·31; 2978·98) | 0·60<br>(0·38;0·90) | 0·58<br>(0·37;0·85) | 0·6081<br>(0·3983;0·8719) | 12·35<br>(7·80;18·42) |
| | **Change (%) 2010-2019** | -2·28<br>(-27·63;29·51) | -6·16<br>(-29·21;22·85) | -7·40<br>(-29·76;19·77) | -11·48<br>(-34·51;18·51) | -11·15<br>(-33·68;17·74) | -14·59<br>(-35·64;11·51) | -15·76<br>(-36·08;8·66) | -19·55<br>(-40·20;6·25) |
| **Estonia** | **2010** | 0·02<br>(0·01;0·03) | 0·02<br>(0·01;0·03) | 20·67<br>(14·83; 27·49) | 372·84<br>(261·57; 498·36) | 0·78<br>(0·55;1·04) | 0·77<br>(0·55;1·01) | 0·8254<br>(0·5987;1·0805) | 15·64<br>(10·95;20·87) |
| | **2019** | 0·03<br>(0·02;0·04) | 0·02<br>(0·02;0·03) | 26·04<br>(16·91; 36·43) | 456·20<br>(290·70; 660·26) | 0·94<br>(0·60;1·37) | 0·87<br>(0·56;1·26) | 0·9096<br>(0·5889;1·2888) | 17·66<br>(11·25;25·67) |
| | **Change (%) 2010-2019** | 33·53<br>(3·61;68·16) | 27·96<br>(-0·01;60·49) | 25·97<br>(-1·16;57·71) | 22·36<br>(-5·81;54·80) | 21·36<br>(-6·42;53·20) | 13·97<br>(-11·38;42·97) | 10·20<br>(-14·01;38·01) | 12·90<br>(-13·98;44·48) |
| **Latvia** | **2010** | 0·03<br>(0·02;0·04) | 0·03<br>(0·02;0·04) | 30·49<br>(21·35; 39·82) | 577·87<br>(408·77; 764·84) | 0·68<br>(0·49;0·91) | 0·70<br>(0·50;0·91) | 0·7578<br>(0·5392;0·9803) | 15·24<br>(10·83;20·39) |
| | **2019** | 0·03<br>(0·02;0·04) | 0·03<br>(0·02;0·04) | 29·63<br>(20·34; 40·76) | 532·81<br>(353·82; 750·04) | 0·64<br>(0·43;0·91) | 0·64<br>(0·43;0·89) | 0·6881<br>(0·4649;0·9475) | 13·75<br>(8·93;19·52) |
| | **Change (%) 2010-2019** | -2·54<br>(-21·39;18·23) | -3·21<br>(-21·62;16·70) | -2·81<br>(-21·00;17·31) | -7·80<br>(-25·76;12·01) | -5·79<br>(-24·05;14·64) | -8·23<br>(-25·82;11·59) | -9·20<br>(-26·33;9·75) | -9·80<br>(-27·24;10·69) |
| **Lithuania** | **2010** | 0·04<br>(0·03;0·05) | 0·04<br>(0·03;0·05) | 40·42<br>(29·02; 53·23) | 771·86<br>(544·19; 1024·21) | 0·71<br>(0·50;0·95) | 0·68<br>(0·49;0·89) | 0·7129<br>(0·5167;0·9358) | 14·51<br>(10·29;19·39) |
| | **2019** | 0·04<br>(0·03;0·06) | 0·04<br>(0·03;0·06) | 45·00<br>(29·52; 61·59) | 830·40<br>(540·20; 1144·10) | 0·78<br>(0·50;1·09) | 0·71<br>(0·47;0·98) | 0·7331<br>(0·4803;0·9995) | 15·02<br>(9·68;20·86) |
| | **Change (%) 2010-2019** | 16·10<br>(-8·89;42·00) | 12·49<br>(-10·72;36·48) | 11·33<br>(-10·97;35·42) | 7·58<br>(-15·04;32·65) | 10·67<br>(-13·58;36·15) | 5·43<br>(-16·72;28·67) | 2·83<br>(-18·17;24·86) | 3·57<br>(-19·33;28·20) |
| **Republic of Moldova** | **2010** | 0·03<br>(0·02;0·04) | 0·03<br>(0·02;0·04) | 34·80<br>(24·78; 46·73) | 732·27<br>(506·21; 997·03) | 0·60<br>(0·42;0·81) | 0·63<br>(0·45;0·84) | 0·6852<br>(0·4917;0·9138) | 14·13<br>(9·82;19·21) |
| | **2019** | 0·03<br>(0·02;0·04) | 0·03<br>(0·02;0·04) | 33·22<br>(22·59; 46·35) | 648·68<br>(431·64; 938·70) | 0·50<br>(0·33;0·71) | 0·52<br>(0·35;0·73) | 0·5680<br>(0·3908;0·7881) | 11·13<br>(7·44;15·86) |
| | **Change (%) 2010-2019** | -5·87<br>(-23·00;14·40) | -5·89<br>(-22·56;13·30) | -4·53<br>(-21·45;14·60) | -11·42<br>(-28·21;7·82) | -16·90<br>(-31·73;0·36) | -17·60<br>(-32·02;-1·06) | -17·11<br>(-31·41;-1·16) | -21·20<br>(-35·79;-4·50) |
| **Russian Federation** | **2010** | 1·39<br>(1·19;1·58) | 1·43<br>(1·25;1·63) | 1559·32<br>(1357·67;1765·07) | 32613·23<br>(27934·48; 37413·74) | 0·65<br>(0·56;0·75) | 0·68<br>(0·59;0·77) | 0·7418<br>(0·6474;0·8380) | 15·44<br>(13·24;17·75) |
| | **2019** | 1·73<br>(1·38;2·10) | 1·76<br>(1·42;2·12) | 1913·29<br>(1585·14;2318·48) | 38784·18<br>(31502·80; 47722·89) | 0·73<br>(0·58;0·88) | 0·74<br>(0·60;0·89) | 0·8024<br>(0·6641;0·9659) | 16·51<br>(13·38;20·20) |
| | **Change (%) 2010-2019** | 24·39<br>(5·96;46·90) | 22·57<br>(5·05;44·06) | 22·70<br>(6·20;43·61) | 18·92<br>(1·42;42·04) | 11·27<br>(-5·02;31·02) | 8·82<br>(-6·52;27·34) | 8·18<br>(-6·32;26·68) | 6·94<br>(-8·68;27·64) |

| | | | | | | | | | |
|---|---|---|---|---|---|---|---|---|---|
| **Ukraine** | **2010** | 0·51<br>(0·44;0·58) | 0·50<br>(0·44;0·57) | 530·76<br>(462·46;600·88) | 11015·26<br>(9437·73; 12645·32) | 0·68<br>(0·59;0·78) | 0·68<br>(0·59;0·77) | 0·7168<br>(0·6286;0·8075) | 15·09<br>(12·93;17·42) |
| | **2019** | 0·53<br>(0·43;0·65) | 0·53<br>(0·44;0·65) | 572·81<br>(466·88;690·17) | 11146·36<br>(8957·69; 13728·43) | 0·68<br>(0·55;0·84) | 0·68<br>(0·56;0·84) | 0·7311<br>(0·5965;0·8859) | 14·68<br>(11·86;18·11) |
| | **Change (%) 2010-2019** | 3·97<br>(-11·49;23·20) | 6·05<br>(-9·70;24·67) | 7·92<br>(-8·68;27·16) | 1·19<br>(-15·11;19·60) | -0·18<br>(-15·74;18·38) | 1·01<br>(-13·96;18·98) | 1·99<br>(-13·62;20·00) | -2·73<br>(-18·30;15·94) |

UI= Uncertainty Interval

**Supplement Table I·** Prevalence, incidence, mortality and DALYs attributable to HCV in 2010 and 2019, by Western Europe countries·

| | | N in thousand (95%UI s | | N (95%UI) | | Age- standardized rate x 100,000 (95%UI) | | | |
|---|---|---|---|---|---|---|---|---|---|
| | **Year** | **Prevalence** | **Incidence** | **Mortality** | **DALYs** | **Prevalence** | **Incidence** | **Mortality** | **DALYs** |
| | | | | **Acute hepatitis** | | | | | |
| **Andorra** | **2010** | 0·00 (0·00;0·01) | 0·04 (0·03;0·05) | 0·10 (0·06;0·16) | 2·71 (1·72; 4·14) | 4·69 (4·08;5·52) | 40·63 (35·32;47·84) | 0·0876 (0·0555;0·1340) | 2·68 (1·73;4·07) |
| | **2019** | 0·00 (0·00;0·01) | 0·04 (0·04;0·05) | 0·07 (0·04;0·12) | 1·75 (1·05; 2·83) | 4·80 (4·17;5·64) | 41·61 (36·13;48·91) | 0·0529 (0·0318;0·0865) | 1·53 (0·93;2·49) |
| | **Change (%) 2010-2019** | 9·05 (1·98;16·57) | 9·05 (1·98;16·57) | -30·58 (-54·73;3·21) | -35·53 (-56·32;-6·43) | 2·39 (-3·20;8·05) | 2·39 (-3·20;8·05) | -39·65 (-61·12;-10·30) | -42·98 (-61·00;-19·40) |
| **Austria** | **2010** | 0·45 (0·38;0·53) | 3·90 (3·33;4·62) | 0·17 (0·09;0·51) | 11·71 (6·95; 21·33) | 4·55 (3·98;5·25) | 39·42 (34·47;45·51) | 0·0015 (0·0009;0·0038) | 0·12 (0·08;0·21) |
| | **2019** | 0·49 (0·41;0·58) | 4·24 (3·57;5·05) | 0·19 (0·11;0·44) | 12·08 (7·40; 20·75) | 4·44 (3·86;5·22) | 38·52 (33·46;45·20) | 0·0013 (0·0008;0·0030) | 0·11 (0·07;0·19) |
| | **Change (%) 2010-2019** | 8·65 (0·07;17·26) | 8·65 (0·07;17·26) | 9·54 (-25·29;72·66) | 3·17 (-16·83;21·68) | -2·29 (-8·16;3·54) | -2·29 (-8·16;3·54) | -8·37 (-33·83;34·92) | -7·99 (-24·53;10·84) |
| **Belgium** | **2010** | 0·58 (0·49;0·67) | 4·99 (4·23;5·83) | 1·58 (0·67;2·11) | 45·09 (23·08; 60·11) | 4·41 (3·83;5·11) | 38·21 (33·21;44·25) | 0·0085 (0·0037;0·0112) | 0·30 (0·16;0·40) |
| | **2019** | 0·63 (0·54;0·75) | 5·47 (4·65;6·50) | 1·65 (0·61;2·31) | 45·09 (21·36; 62·37) | 4·44 (3·90;5·13) | 38·47 (33·81;44·42) | 0·0078 (0·0030;0·0108) | 0·27 (0·14;0·38) |
| | **Change (%) 2010-2019** | 9·65 (0·73;20·01) | 9·65 (0·73;20·01) | 4·23 (-19·33;27·34) | 0·01 (-18·19;18·10) | 0·70 (-5·95;7·83) | 0·70 (-5·95;7·83) | -8·45 (-28·36;11·28) | -8·90 (-24·81;6·91) |
| **Cyprus** | **2010** | 0·04 (0·04;0·05) | 0·38 (0·32;0·45) | 0·02 (0·01;0·05) | 1·38 (0·95; 2·18) | 3·70 (3·21;4·32) | 32·10 (27·78;37·44) | 0·0017 (0·0011;0·0036) | 0·13 (0·09;0·19) |
| | **2019** | 0·06 (0·05;0·07) | 0·48 (0·40;0·57) | 0·02 (0·01;0·05) | 1·58 (1·03; 2·48) | 3·67 (3·15;4·31) | 31·81 (27·27;37·33) | 0·0014 (0·0009;0·0027) | 0·12 (0·07;0·18) |
| | **Change (%) 2010-2019** | 24·64 (15·61;34·25) | 24·64 (15·61;34·25) | 5·18 (-25·67;66·21) | 14·44 (-10·41;50·40) | -0·91 (-7·52;5·32) | -0·91 (-7·52;5·32) | -18·02 (-44·36;31·10) | -7·85 (-31·87;28·89) |
| **Denmark** | **2010** | 0·27 (0·23;0·32) | 2·34 (1·96;2·76) | 0·20 (0·10;0·33) | 9·58 (6·18; 14·62) | 4·22 (3·65;4·90) | 36·55 (31·66;42·50) | 0·0024 (0·0013;0·0041) | 0·14 (0·09;0·22) |
| | **2019** | 0·28 (0·24;0·33) | 2·42 (2·06;2·90) | 0·18 (0·09;0·30) | 8·97 (5·43; 13·79) | 3·98 (3·46;4·71) | 34·49 (29·98;40·81) | 0·0020 (0·0010;0·0032) | 0·13 (0·08;0·19) |
| | **Change (%) 2010-2019** | 3·45 (-6·15;13·20) | 3·45 (-6·15;13·20) | -8·55 (-30·16;23·17) | -6·40 (-22·33;11·23) | -5·63 (-12·84;1·41) | -5·63 (-12·84;1·41) | -19·01 (-37·16;6·08) | -12·56 (-26·97;2·57) |
| **Finland** | **2010** | 0·29 (0·25;0·34) | 2·53 (2·17;2·99) | 0·14 (0·08;0·34) | 8·00 (4·94; 13·99) | 4·54 (3·97;5·25) | 39·38 (34·40;45·49) | 0·0017 (0·0010;0·0040) | 0·13 (0·08;0·21) |
| | **2019** | 0·31 (0·26;0·37) | 2·69 (2·28;3·18) | 0·12 (0·07;0·27) | 7·60 (4·63; 13·09) | 4·45 (3·90;5·21) | 38·54 (33·77;45·13) | 0·0014 (0·0008;0·0030) | 0·11 (0·07;0·19) |
| | **Change (%) 2010-2019** | 6·34 (-2·44;15·51) | 6·34 (-2·44;15·51) | -7·87 (-32·28;38·96) | -4·99 (-21·72;11·09) | -2·15 (-8·31;4·79) | -2·15 (-8·31;4·79) | -18·34 (-38·70;14·17) | -10·54 (-26·33;6·62) |

| | | | | | | | | | |
|---|---|---|---|---|---|---|---|---|---|
| **France** | **2010** | 3·42<br>(2·92;4·03) | 29·61<br>(25·33;34·89) | 2·53<br>(1·35;4·33) | 125·13<br>(75·60; 187·97) | 4·44<br>(3·88;5·10) | 38·49<br>(33·61;44·20) | 0·0027<br>(0·0014;0·0044) | 0·17<br>(0·10;0·25) |
| | **2019** | 3·59<br>(3·07;4·24) | 31·09<br>(26·59;36·71) | 2·32<br>(1·09;3·89) | 117·03<br>(70·07; 180·56) | 4·25<br>(3·72;4·89) | 36·85<br>(32·23;42·39) | 0·0023<br>(0·0011;0·0036) | 0·15<br>(0·08;0·22) |
| | **Change (%) 2010-2019** | 5·01<br>(-4·70;13·91) | 5·01<br>(-4·70;13·91) | -8·47<br>(-28·26;16·08) | -6·48<br>(-20·48;8·51) | -4·27<br>(-10·93;2·43) | -4·27<br>(-10·93;2·43) | -16·40<br>(-34·76;6·70) | -11·41<br>(-25·24;4·31) |
| **Germany** | **2010** | 3·78<br>(3·28;4·43) | 32·77<br>(28·44;38·39) | 4·34<br>(2·33;6·99) | 3781·32<br>(3281·93; 4429·23) | 3·57<br>(3·19;4·01) | 30·90<br>(27·67;34·73) | 0·0031<br>(0·0017;0·0050) | 0·16<br>(0·10;0·22) |
| | **2019** | 4·21<br>(3·55;5·08) | 36·45<br>(30·75;44·04) | 2·74<br>(1·50;7·23) | 4205·51<br>(3548·38; 5081·24) | 3·77<br>(3·30;4·43) | 32·67<br>(28·63;38·41) | 0·0018<br>(0·0010;0·0045) | 0·12<br>(0·07;0·20) |
| | **Change (%) 2010-2019** | 11·22<br>(1·62;22·74) | 11·22<br>(1·62;22·74) | -36·77<br>(-63·70;8·75) | -24·49<br>(-46·50;4·66) | 5·72<br>(-2·06;13·49) | 5·72<br>(-2·06;13·49) | -41·73<br>(-64·43;-3·75) | -26·19<br>(-46·40;-0·74) |
| **Greece** | **2010** | 0·39<br>(0·32;0·46) | 3·35<br>(2·80;4·00) | 5·75<br>(2·25;16·59) | 110·62<br>(54·43; 278·43) | 2·77<br>(2·38;3·26) | 24·01<br>(20·60;28·27) | 0·0256<br>(0·0116;0·0683) | 0·68<br>(0·39;1·46) |
| | **2019** | 0·39<br>(0·32;0·48) | 3·42<br>(2·80;4·13) | 2·46<br>(1·32;5·98) | 49·58<br>(29·97; 106·42) | 2·78<br>(2·38;3·24) | 24·06<br>(20·65;28·12) | 0·0102<br>(0·0059;0·0232) | 0·31<br>(0·20;0·57) |
| | **Change (%) 2010-2019** | 2·24<br>(-6·86;11·92) | 2·24<br>(-6·86;11·92) | -57·27<br>(-70·22;-21·93) | -55·18<br>(-68·53;-27·78) | 0·19<br>(-5·74;7·33) | 0·19<br>(-5·74;7·33) | -60·14<br>(-72·44;-31·63) | -53·92<br>(-68·20;-26·16) |
| **Iceland** | **2010** | 0·01<br>(0·01;0·01) | 0·10<br>(0·08;0·12) | 0·00<br>(0·00;0·00) | 0·22<br>(0·13; 0·39) | 3·33<br>(2·88;3·97) | 28·84<br>(24·95;34·38) | 0·0004<br>(0·0002;0·0009) | 0·06<br>(0·04;0·11) |
| | **2019** | 0·01<br>(0·01;0·02) | 0·11<br>(0·10;0·13) | 0·00<br>(0·00;0·00) | 0·24<br>(0·14; 0·43) | 3·31<br>(2·86;3·88) | 28·72<br>(24·82;33·66) | 0·0004<br>(0·0002;0·0009) | 0·06<br>(0·04;0·11) |
| | **Change (%) 2010-2019** | 13·32<br>(4·31;22·77) | 13·32<br>(4·31;22·77) | 11·14<br>(-14·89;45·66) | 10·94<br>(-1·71;23·28) | -0·41<br>(-7·07;5·88) | -0·41<br>(-7·07;5·88) | -2·69<br>(-27·59;34·12) | -0·20<br>(-12·32;13·97) |
| **Ireland** | **2010** | 0·20<br>(0·17;0·23) | 1·71<br>(1·47;2·00) | 0·38<br>(0·21;0·57) | 16·29<br>(9·74; 22·62) | 4·04<br>(3·51;4·71) | 34·98<br>(30·40;40·81) | 0·0070<br>(0·0040;0·0104) | 0·32<br>(0·19;0·44) |
| | **2019** | 0·22<br>(0·19;0·26) | 1·93<br>(1·63;2·24) | 0·25<br>(0·17;0·44) | 11·04(7·56; 16·82) | 4·03<br>(3·49;4·69) | 34·92<br>(30·25;40·65) | 0·0038<br>(0·0025;0·0066) | 0·19<br>(0·13;0·29) |
| | **Change (%) 2010-2019** | 12·82<br>(4·82;22·13) | 12·82<br>(4·82;22·13) | -34·02<br>(-50·25;-8·21) | -32·25<br>(-46·89;-8·70) | -0·15<br>(-6·00;6·53) | -0·15<br>(-6·00;6·53) | -45·22<br>(-58·23;-24·38) | -39·67<br>(-52·17;-20·22) |
| **Israel** | **2010** | 0·35<br>(0·30;0·41) | 3·00<br>(2·58;3·52) | 0·47<br>(0·33;0·82) | 19·72<br>(14·08; 31·36) | 4·25<br>(3·68;5·01) | 36·87<br>(31·87;43·44) | 0·0054<br>(0·0039;0·0098) | 0·24<br>(0·17;0·39) |
| | **2019** | 0·42<br>(0·36;0·50) | 3·64<br>(3·15;4·32) | 0·41<br>(0·27;0·80) | 18·68<br>(12·72; 30·94) | 4·22<br>(3·65;5·00) | 36·58<br>(31·62;43·33) | 0·0038<br>(0·0025;0·0075) | 0·19<br>(0·13;0·31) |
| | **Change (%) 2010-2019** | 21·44<br>(12·49;31·07) | 21·44<br>(12·49;31·07) | -11·23<br>(-32·68;9·25) | -5·25<br>(-22·88;12·81) | -0·79<br>(-8·20;7·11) | -0·79<br>(-8·20;7·11) | -29·54<br>(-46·51;-13·75) | -22·50<br>(-36·75;-7·92) |
| **Italy** | **2010** | 6·33<br>(5·29;7·62) | 54·83<br>(45·85;66·04) | 6·57<br>(4·20;17·69) | 237·53<br>(147·10; 524·57) | 8·90<br>(7·65;10·53) | 77·17<br>(66·29;91·24) | 0·0057<br>(0·0035;0·0163) | 0·29<br>(0·17;0·67) |
| | **2019** | 5·91<br>(4·88;7·21) | 51·20<br>(42·28;62·46) | 5·84<br>(3·76;14·15) | 205·87<br>(127·15; 412·95) | 7·81<br>(6·69;9·28) | 67·72<br>(57·95;80·41) | 0·0045<br>(0·0028;0·0115) | 0·24<br>(0·14;0·47) |
| | **Change (%) 2010-2019** | -6·63<br>(-9·68;-3·70) | -6·63<br>(-9·68;-3·70) | -11·17<br>(-28·32;18·77) | -13·33<br>(-28·89;4·69) | -12·24<br>(-13·63;-10·88) | -12·24<br>(-13·63;-10·88) | -21·82<br>(-37·61;4·55) | -19·10<br>(-34·75;-2·68) |
| **Luxembourg** | **2010** | 0·03 | 0·22 | 0·01 | 0·69 | 4·41 | 38·20 | 0·0016 | 0·12 |

| | | | | | | | | | |
|---|---|---|---|---|---|---|---|---|---|
| | | (0·02;0·03) | (0·19;0·26) | (0·01;0·02) | (0·43; 1·09) | (3·86;5·16) | (33·46;44·69) | (0·0008;0·0027) | (0·07;0·18) |
| | **2019** | 0·03 (0·03;0·04) | 0·27 (0·23;0·32) | 0·01 (0·00;0·02) | 0·71 (0·45; 1·19) | 4·34 (3·77;5·03) | 37·61 (32·69;43·63) | 0·0010 (0·0005;0·0020) | 0·10 (0·06;0·16) |
| | **Change (%) 2010-2019** | 23·61 (14·25;34·70) | 23·61 (14·25;34·70) | -21·61 (-42·75;5·35) | 2·11 (-14·67;19·91) | -1·54 (-7·21;5·59) | -1·54 (-7·21;5·59) | -36·36 (-52·87;-13·78) | -16·40 (-29·91;-3·06) |
| **Malta** | **2010** | 0·02 (0·02;0·02) | 0·16 (0·14;0·19) | 0·01 (0·00;0·02) | 0·56 (0·32; 0·87) | 3·78 (3·29;4·39) | 32·77 (28·51;38·07) | 0·0016 (0·0007;0·0026) | 0·11 (0·06;0·17) |
| | **2019** | 0·02 (0·02;0·03) | 0·18 (0·15;0·22) | 0·01 (0·00;0·01) | 0·52 (0·30; 0·87) | 3·77 (3·26;4·44) | 32·68 (28·23;38·46) | 0·0011 (0·0005;0·0021) | 0·09 (0·06;0·16) |
| | **Change (%) 2010-2019** | 12·32 (1·85;23·62) | 12·32 (1·85;23·62) | -19·79 (-42·30;5·03) | -6·20 (-22·94;9·12) | -0·26 (-7·34;7·57) | -0·26 (-7·34;7·57) | -30·34 (-48·77;-8·73) | -14·73 (-29·23;0·07) |
| **Monaco** | **2010** | 0·00 (0·00;0·00) A | 0·02 (0·02;0·02) | 0·00 (0·00;0·00) | 0·07 (0·05; 0·11) | 4·89 (4·26;5·67) | 42·41 (36·92;49·13) | 0·0023 (0·0012;0·0036) | 0·14 (0·09;0·21) |
| | **2019** | 0·00 (0·00;0·00) B | 0·02 (0·02;0·03) | 0·00 (0·00;0·00) | 0·08 (0·04; 0·12) | 4·89 (4·25;5·72) | 42·35 (36·81;49·55) | 0·0021 (0·0010;0·0035) | 0·13 (0·08;0·21) |
| | **Change (%) 2010-2019** | 7·70 (-0·63;16·87) | 7·70 (-0·63;16·87) | -2·67 (-39·96;61·66) | 4·72 (-21·31;39·94) | -0·15 (-5·68;6·18) | -0·15 (-5·68;6·18) | -8·81 (-43·66;49·31) | -4·89 (-26·00;24·06) |
| **Netherlands** | **2010** | 0·35 (0·30;0·41) | 3·06 (2·62;3·59) | 0·51 (0·21;0·69) | 18·92 (10·24; 26·29) | 1·71 (1·49;1·98) | 14·83 (12·90;17·16) | 0·0020 (0·0009;0·0028) | 0·09 (0·05;0·13) |
| | **2019** | 0·56 (0·47;0·67) | 4·85 (4·11;5·80) | 0·56 (0·19;0·83) | 22·66 (11·33; 34·09) | 2·46 (2·13;2·88) | 21·31 (18·50;24·93) | 0·0020 (0·0007;0·0029) | 0·10 (0·05;0·15) |
| | **Change (%) 2010-2019** | 58·51 (44·44;73·29) | 58·51 (44·44;73·29) | 10·25 (-17·64;48·84) | 19·74 (-5·41;50·17) | 43·70 (33·11;55·04) | 43·70 (33·11;55·04) | -2·72 (-26·26;30·93) | 10·38 (-12·42;39·49) |
| **Norway** | **2010** | 0·24 (0·20;0·29) | 2·09 (1·74;2·51) | 0·08( 0·04;0·15) | 6·07 (3·63; 9·79) | 4·38 (3·74;5·23) | 37·96 (32·41;45·30) | 0·0012 (0·0006;0·0021) | 0·11 (0·06;0·17) |
| | **2019** | 0·28 (0·23;0·33) | 2·40 (1·99;2·88) | 0·06 (0·03;0·12) | 5·76 (3·50; 9·94) | 4·46 (3·80;5·34) | 38·66 (32·91;46·27) | 0·0008 (0·0004;0·0015) | 0·09 (0·06;0·16) |
| | **Change (%) 2010-2019** | 14·66 (12·04;17·61) | 14·66 (12·04;17·61) | -28·15 (-40·95;-6·24) | -5·12 (-16·21;7·22) | 1·85 (0·77;2·85) | 1·85 (0·77;2·85) | -36·25 (-45·49;-17·58) | -14·06 (-23·43;-4·25) |
| **Portugal** | **2010** | 0·57 (0·49;0·68) | 4·95 (4·21;5·89) | 0·99 (0·40;1·31) | 40·01 (18·86; 54·18) | 4·50 (3·92;5·24) | 39·02 (33·96;45·41) | 0·0062 (0·0024;0·0083) | 0·30 (0·14;0·41) |
| | **2019** | 0·59 (0·50;0·71) | 5·12 (4·34;6·11) | 0·60 (0·25;0·87) | 26·22 (13·76; 37·52) | 4·37 (3·84;5·02) | 37·87 (33·31;43·55) | 0·0034 (0·0014;0·0049) | 0·19 (0·10;0·28) |
| | **Change (%) 2010-2019** | 3·48 (-4·95;14·28) | 3·48 (-4·95;14·28) | -39·23 (-56·29;-16·39) | -34·47 (-47·60;-14·13) | -2·94 (-9·16;4·02) | -2·94 (-9·16;4·02) | -44·91 (-59·38;-25·16) | -36·98 (-49·41;-18·41) |
| **San Marino** | **2010** | 0·00 (0·00;0·00) C | 0·01 (0·01;0·02) | 0·02 (0·01;0·03) | 0·52 (0·21; 0·83) | 4·25 (3·64;4·96) | 36·79 (31·52;43·02) | 0·0375 (0·0153;0·0606) | 1·43 (0·57;2·29) |
| | **2019** | 0·00 (0·00;0·00) D | 0·02 (0·01;0·02) | 0·01 (0·01;0·03) | 0·46 (0·21; 0·77) | 4·27 (3·69;5·03) | 37·00 (31·94;43·59) | 0·0292 (0·0133;0·0494) | 1·12 (0·51;1·87) |
| | **Change (%) 2010-2019** | 12·14 (5·59;18·91) | 12·14 (5·59;18·91) | -10·63 (-47·05;47·43) | -11·72 (-44·43;40·26) | 0·57 (-3·83;5·19) | 0·57 (-3·83;5·19) | -22·01 (-51·52;25·44) | -21·59 (-50·77;24·06) |
| **Spain** | **2010** | 2·86 (2·45;3·40) | 24·79 (21·21;29·48) | 6·39 (4·00;8·21) | 225·59 (150·18; 288·67) | 5·06 (4·42;5·85) | 43·87 (38·35;50·73) | 0·0090 (0·0057;0·0119) | 0·38 (0·27;0·50) |

| | | | | | | | | | |
|---|---|---|---|---|---|---|---|---|---|
| | **2019** | 2·87 (2·43;3·36) | 24·86 (21·06;29·14) | 4·98 (3·25;7·14) | 175·80 (120·91; 243·23) | 4·76 (4·15;5·47) | 41·29 (36·00;47·40) | 0·0064 (0·0041;0·0091) | 0·28 (0·20;0·41) |
| | **Change (%) 2010-2019** | 0·29 (-10·74;11·82) | 0·29 (-10·74;11·82) | -22·07 (-37·15;-0·97) | -22·07 (-34·71;-5·98) | -5·88 (-14·65;3·48) | -5·88 (-14·65;3·48) | -29·61 (-42·75;-10·89) | -25·87 (-37·94;-10·45) |
| **Sweden** | **2010** | 0·43 (0·35;0·52) | 3·72 (3·06;4·51) | 0·26 (0·17;0·57) | 13·86 (9·15; 22·53) | 3·73 (3·18;4·42) | 32·33 (27·56;38·28) | 0·0019 (0·0012;0·0037) | 0·12 (0·08;0·19) |
| | **2019** | 0·48 (0·40;0·59) | 4·18 (3·43;5·09) | 0·21 (0·12;0·52) | 12·79 (8·16; 22·22) | 3·77 (3·19;4·47) | 32·64 (27·68;38·72) | 0·0013 (0·0008;0·0031) | 0·10 (0·07;0·17) |
| | **Change (%) 2010-2019** | 12·27 (8·19;17·13) | 12·27 (8·19;17·13) | -17·91 (-40·20;7·66) | -7·71 (-21·78;7·21) | 0·97 (-1·41;3·75) | 0·97 (-1·41;3·75) | -29·04 (-46·99;-5·32) | -16·61 (-29·52;-2·49) |
| **Switzerland** | **2010** | 0·39 (0·33;0·47) | 3·39 (2·89;4·04) | 2·27 (1·17;2·77) | 62·55 (34·43; 76·58) | 4·12 (3·58;4·83) | 35·74 (31·04;41·87) | 0·0173 (0·0090;0·0210) | 0·57 (0·33;0·69) |
| | **2019** | 0·45 (0·38;0·54) | 3·94 (3·33;4·67) | 2·25 (1·07;2·80) | 60·64 (31·54; 75·80) | 4·17 (3·62;4·87) | 36·14 (31·35;42·19) | 0·0145 (0·0070;0·0181) | 0·48 (0·26;0·60) |
| | **Change (%) 2010-2019** | 16·28 (7·18;26·15) | 16·28 (7·18;26·15) | -0·96 (-16·11;15·48) | -3·06 (-15·98;9·65) | 1·11 (-5·73;8·44) | 1·11 (-5·73;8·44) | -16·29 (-29·07;-3·25) | -15·22 (-26·53;-3·76) |
| **United Kingdom** | **2010** | 5·65 (4·78;6·74) | 48·98 (41·41;58·43) | 2·40 (1·71;5·41) | 158·43 (100·25; 263·23) | 8·24 (7·08;9·78) | 71·45 (61·38;84·80) | 0·0028 (0·0020;0·0061) | 0·22 (0·14;0·37) |
| | **2019** | 4·02 (3·40;4·78) | 34·87 (29·49;41·45) | 1·52 (0·77;4·69) | 103·65 (57·14; 200·64) | 5·13 (4·43;6·04) | 44·49 (38·40;52·36) | 0·0016 (0·0008;0·0047) | 0·13 (0·08;0·25) |
| | **Change (%) 2010-2019** | -28·81 (-31·69;-25·63) | -28·81 (-31·69;-25·63) | -36·62 (-59·71;-4·14) | -34·58 (-47·63;-18·88) | -37·74 (-39·26;-36·05) | -37·74 (-39·26;-36·05) | -42·63 (-62·36;-15·66) | -40·36 (-51·46;-27·65) |
| **Cirrhosis and other chronic liver diseases due to hepatitis** | | | | | | | | | |
| **Andorra** | **2010** | 0·71 (0·58;0·87) | 0·01 (0·00;0·01) | 1·94 (1·29; 2·79) | 56·78 (37·09; 81·79) | 664·78 (545·12;822·40) | 4·19 (2·70;6·23) | 1·5842 (1·0511; 2·3050) | 46·73 (30·86;66·97) |
| | **2019** | 0·77 (0·64;0·94) | 0·00 (0·00;0·01) | 2·12 (1·33; 3·16) | 58·52 (36·68; 88·69) | 679·00 (556·01;823·85) | 3·93 (2·46;5·88) | 1·5040 (0·9568; 2·2473) | 43·93 (27·61;66·09) |
| | **Change (%) 2010-2019** | 9·68 (3·57;16·64) | -11·31 (-19·63;-2·66) | 8·93 (-13·12;37·86) | 3·07 (-18·20;31·03) | 2·14 (-3·03;8·33) | -6·29 (-15·40;2·74) | -5·06 (-23·92;19·47) | -6·00 (-25·59;19·82) |
| **Austria** | **2010** | 76·94 (63·65;94·70) | 0·74 (0·45;1·10) | 321·47 (227·25;446·68) | 8868·84 (6277·76; 12424·51) | 683·70 (558·75;851·39) | 7·05 (4·42;10·42) | 2·3453 (1·6686; 3·2973) | 70·33 (49·43;98·25) |
| | **2019** | 82·76 (67·90;100·44) | 0·60 (0·38;0·89) | 301·32 (212·68;417·54) | 7853·08 (5547·17; 10952·89) | 666·74 (542·17;819·57) | 5·86 (3·74;8·56) | 1·9108 (1·3558; 2·6433) | 55·95 (40·00;77·03) |
| | **Change (%) 2010-2019** | 7·55 (0·84;14·61) | -19·76 (-28·10;-10·16) | -6·27 (-14·51;3·81) | -11·45 (-19·17;-2·14) | -2·48 (-8·43;3·32) | -16·90 (-24·80;-7·18) | -18·53 (-24·98;-10·30) | -20·45 (-26·87;-12·63) |
| **Belgium** | **2010** | 95·48 (77·70;116·08) | 0·80 (0·52;1·15) | 457·70 (338·98;600·81) | 11586·53 (8577·11; 15343·51) | 656·83 (534·84;798·51) | 6·32 (4·16;8·90) | 2·5190 (1·9038; 3·3145) | 71·24 (53·05;93·77) |
| | **2019** | 103·66 (85·08;127·13) | 0·74 (0·48;1·08) | 469·52 (343·93;624·72) | 11303·83 (8281·41; 15265·22) | 655·72 (533·15;801·18) | 5·96 (3·87;8·53) | 2·2727 (1·6811; 3·0361) | 63·73 (46·86;85·98) |
| | **Change (%) 2010-2019** | 8·57 (1·12;16·40) | -7·48 (-18·47;3·18) | 2·58 (-7·31;12·82) | -2·44 (-10·82;7·02) | -0·17 (-6·36;6·66) | -5·70 (-16·43;5·04) | -9·78 (-17·47;-1·55) | -10·55 (-17·56;-2·34) |
| **Cyprus** | **2010** | 6·79 (5·52;8·35) | 0·05 (0·03;0·08) | 20·45 (14·85; 28·75) | 480·37 (354·47; 657·55) | 523·59 (423·48;644·63) | 3·88 (2·53;5·65) | 1·5986 (1·1450; 2·2060) | 35·16 (26·15;47·34) |

| | | | | | | | | | |
|---|---|---|---|---|---|---|---|---|---|
| | **2019** | 8·47 (6·86;10·37) | 0·06 (0·04;0·09) | 24·04 (16·77; 34·85) | 549·61 (388·84; 774·60) | 518·08 (420·11;636·20) | 3·69 (2·36;5·45) | 1·3593 (0·9622; 1·9193) | 30·44 (21·84;42·45) |
| | **Change (%) 2010-2019** | 24·75 (16·41;33·04) | 15·90 (1·37;32·11) | 17·52 (0·40;37·14) | 14·41 (-2·80;33·39) | -1·05 (-7·19;5·18) | -4·91 (-16·02;6·76) | -14·97 (-27·27;-1·46) | -13·42 (-26·35;1·17) |
| **Denmark** | **2010** | 46·34 (37·62;56·53) | 0·28 (0·18;0·42) | 175·21 (123·49;249·69) | 5021·19 (3427·59; 7209·16) | 644·79 (521·62;792·43) | 4·60 (2·90;6·78) | 2·0146 (1·4109; 2·8465) | 62·01 (42·72;88·10) |
| | **2019** | 47·54 (38·72;58·81) | 0·23 (0·14;0·34) | 152·44 (107·70;215·67) | 4088·95 (2815·93; 5844·04) | 601·21 (487·24;744·05) | 3·88 (2·43;5·77) | 1·5577 (1·1029; 2·2162) | 46·79 (32·58;66·66) |
| | **Change (%) 2010-2019** | 2·58 (-5·39;10·08) | -18·53 (-28·10;-8·16) | -12·99 (-22·13;-2·58) | -18·57 (-27·02;-9·38) | -6·76 (-13·14;-0·13) | -15·64 (-24·66;-5·93) | -22·68 (-30·36;-14·00) | -24·54 (-32·08;-16·03) |
| **Finland** | **2010** | 49·63 (40·66;60·27) | 0·25 (0·16;0·37) | 216·41 (146·49;312·16) | 6790·70 (4555·43; 9744·96) | 686·33 (558·09;836·02) | 4·78 (2·97;7·06) | 2·5544 (1·7443; 3·6277) | 86·54 (59·23;122·03) |
| | **2019** | 52·41 (42·71;64·07) | 0·22 (0·14;0·33) | 185·89 (128·91;268·42) | 5429·98 (3727·09; 7790·22) | 664·97 (541·57;810·36) | 4·21 (2·62;6·20) | 2·0492 (1·4178; 2·9198) | 67·88 (46·75;95·08) |
| | **Change (%) 2010-2019** | 5·60 (-1·54;13·98) | -11·30 (-20·65;-1·12) | -14·10 (-23·71;-4·20) | -20·04 (-28·31;-11·37) | -3·11 (-9·39;4·08) | -11·94 (-21·67;-2·16) | -19·78 (-27·18;-11·74) | -21·56 (-29·20;-13·34) |
| **France** | **2010** | 538·88 (441·26;663·43) | 3·32 (2·09;4·98) | 1927·12 (1392·40;2626·53) | 52016·59 (37060·79; 72597·76) | 640·21 (522·66;786·72) | 4·71 (2·99;7·03) | 1·9020 (1·3750; 2·6046) | 57·19 (40·42;79·98) |
| | **2019** | 564·90 (464·40;691·77) | 2·89 (1·78;4·34) | 1849·91 (1322·51;2507·45) | 45807·23(32203·07; 63192·32) | 610·91 (496·96;745·64) | 4·20 (2·64;6·22) | 1·5693(1·1215; 2·1433) | 45·98 (32·71;62·99) |
| | **Change (%) 2010-2019** | 4·83 (-2·90;12·92) | -12·95 (-22·51;-3·45) | -4·01 (-13·61;6·53) | -11·94 (-21·08;-2·52) | -4·58 (-10·92;2·06) | -10·95 (-20·88;-0·76) | -17·49 (-25·17;-9·21) | -19·60 (-27·06;-11·22) |
| **Germany** | **2010** | 594·86 (496·95;703·75) | 6·32 (3·84;9·35) | 3198·92 (2326·75;4416·22) | 85856·36 (61951·59; 118214·30) | 506·90 (423·19;598·69) | 6·44 (4·01;9·36) | 2·1869 (1·5792; 3·0070) | 65·66 (47·10;89·69) |
| | **2019** | 689·64 (564·54;847·31) | 5·07 (3·18;7·43) | 3332·41 (2344·29;4576·30) | 83355·01 (57653·66; 116217·70) | 548·22 (448·91;672·89) | 5·65 (3·58;8·19) | 2·0537 (1·4624; 2·8183) | 60·39 (42·74;83·01) |
| | **Change (%) 2010-2019** | 15·93 (6·80;25·08) | -19·91 (-29·18;-9·05) | 4·17 (-5·06;15·05) | -2·91 (-11·31;7·63) | 8·15 (0·25;16·35) | -12·34 (-20·81;-2·00) | -6·09 (-13·78;3·08) | -8·03 (-15·43;0·59) |
| **Greece** | **2010** | 61·62 (50·63;75·36) | 0·42 (0·27;0·62) | 213·38 (151·67;295·07) | 5116·13 (3785·22; 6949·53) | 401·63 (326·88;495·57) | 3·20 (2·09;4·69) | 1·1054 (0·8020; 1·4946) | 30·30 (21·87;41·21) |
| | **2019** | 63·04 (51·36;77·20) | 0·35 (0·22;0·53) | 229·29 (161·11;310·17) | 5062·11 (3714·11; 6869·45) | 402·23 (328·88;494·80) | 2·95 (1·87;4·38) | 1·0566 (0·7710; 1·4369) | 28·72 (21·09;39·15) |
| | **Change (%) 2010-2019** | 2·30 (-4·35;9·99) | -16·07 (-25·70;-5·98) | 7·45 (-3·39;19·07) | -1·06 (-10·39;8·66) | 0·15 (-5·69;6·53) | -7·62 (-17·79;2·22) | -4·42 (-13·06;4·62) | -5·19 (-13·93;4·14) |
| **Iceland** | **2010** | 1·90 (1·54;2·34) | 0·00 (0·00;0·00) E | 1·27 (0·89; 1·80) | 35·78 (24·71; 50·26) | 511·05 (413·32;635·15) | 0·93 (0·57;1·37) | 0·3043 (0·2127; 0·4338) | 8·94 (6·11;12·56) |
| | **2019** | 2·17 (1·77;2·65) | 0·00 (0·00;0·00) F | 1·44 (0·98; 2·05) | 39·85 (27·08; 58·01) | 505·28 (408·42;623·27) | 0·87 (0·52;1·34) | 0·2846 (0·1969; 0·4021) | 8·63 (5·93;12·61) |
| | **Change (%) 2010-2019** | 14·33 (7·65;21·17) | -2·50 (-15·36;10·53) | 12·80 (-3·62;33·21) | 11·35 (-5·01;32·66) | -1·13 (-6·80;4·38) | -6·01 (-18·66;6·41) | -6·45 (-19·80;11·11) | -3·51 (-16·96;14·54) |
| **Ireland** | **2010** | 32·03 (26·07;39·37) | 0·16 (0·10;0·23) | 64·31 (46·70; 86·97) | 1976·13 (1422·55; 2692·51) | 613·87 (500·23;755·25) | 2·96 (1·88;4·28) | 1·1390 (0·8291; 1·5387) | 35·62 (25·59;48·74) |
| | **2019** | 36·45 | 0·16 | 66·21 | 1868·94 | 607·80 | 2·96 | 0·9507 | 28·51 |

| | | | | | | | | | |
|---|---|---|---|---|---|---|---|---|---|
| | | (29·83;44·74) | (0·10;0·24) | (47·81; 90·86) | (1309·98; 2596·79) | (489·86;747·29) | (1·92;4·33) | (0·6879; 1·3024) | (20·33;39·73) |
| | **Change (%) 2010-2019** | 13·81<br>(6·95;20·95) | 4·45<br>(-6·56;16·84) | 2·95<br>(-9·00;15·39) | -5·42<br>(-16·33;5·85) | -0·99<br>(-6·75;4·71) | 0·06<br>(-9·77;11·06) | -16·53<br>(-25·54;-7·02) | -19·95<br>(-28·55;-10·76) |
| **Israel** | **2010** | 51·83<br>(42·22;64·09) | 0·31<br>(0·20;0·45) | 121·54<br>(86·04;164·10) | 2699·38<br>(1998·45; 3604·95) | 635·57<br>(515·09;787·77) | 4·04<br>(2·62;5·82) | 1·3767<br>(0·9818; 1·8570) | 32·20<br>(23·74;43·01) |
| | **2019** | 63·64<br>(51·84;79·47) | 0·34<br>(0·22;0·50) | 142·17<br>(99·97;195·25) | 3024·66<br>(2209·58; 4027·98) | 634·75<br>(515·28;793·71) | 3·71<br>(2·38;5·36) | 1·2237<br>(0·8650; 1·6544) | 28·49<br>(21·11;37·86) |
| | **Change (%) 2010-2019** | 22·78<br>(14·53;31·59) | 9·75<br>(-2·82;23·11) | 16·97<br>(5·70;29·40) | 12·05<br>(1·67;23·50) | -0·13<br>(-7·16;6·92) | -8·04<br>(-18·70;3·04) | -11·11<br>(-19·29;-2·40) | -11·52<br>(-19·78;-2·86) |
| **Italy** | **2010** | 1181·11<br>(961·28;1443·45) | 10·68<br>(8·60;13·03) | 4092·42<br>(3510·29;4699·95) | 89521·68<br>(79113·68; 100976·92) | 1401·95<br>(1140·94;1720·50) | 13·97<br>(11·33;16·83) | 3·4090<br>(2·9812; 3·8808) | 87·17<br>(77·49;98·31) |
| | **2019** | 1076·37<br>(870·98;1320·49) | 9·19<br>(6·90;11·78) | 4012·36<br>(3442·18;4632·13) | 82862·56<br>(72954·72; 93802·35) | 1214·18<br>(981·91;1497·73) | 12·04<br>(9·30;15·02) | 2·9103<br>(2·5338; 3·3104) | 73·44<br>(65·13;82·77) |
| | **Change (%) 2010-2019** | -8·87<br>(-10·68;-6·78) | -13·94<br>(-22·33;-6·37) | -1·96<br>(-5·70;2·00) | -7·44<br>(-10·70;-3·83) | -13·39<br>(-14·97;-11·71) | -13·77<br>(-20·88;-7·66) | -14·63<br>(-17·56;-11·31) | -15·75<br>(-18·58;-12·66) |
| **Luxembourg** | **2010** | 4·13<br>(3·40;5·02) | 0·04<br>(0·03;0·06) | 14·79<br>(10·76; 20·03) | 407·52<br>(291·27; 553·32) | 656·89<br>(537·84;803·35) | 6·53<br>(4·08;9·61) | 2·0115<br>(1·4696; 2·7161) | 58·28<br>(41·37;79·55) |
| | **2019** | 5·17<br>(4·25;6·27) | 0·04<br>(0·02;0·06) | 15·45<br>(11·08; 21·31) | 422·43<br>(298·21; 591·90) | 649·77<br>(533·65;795·62) | 5·19<br>(3·28;7·69) | 1·6187<br>(1·1628; 2·2384) | 47·49<br>(33·89;65·85) |
| | **Change (%) 2010-2019** | 25·14<br>(17·60;34·29) | -5·13<br>(-15·24;5·49) | 4·42<br>(-10·58;21·04) | 3·66<br>(-11·05;20·21) | -1·08<br>(-6·42;5·64) | -20·54<br>(-28·46;-12·41) | -19·53<br>(-30·61;-6·79) | -18·51<br>(-30·07;-5·21) |
| **Malta** | **2010** | 3·20<br>(2·61;3·91) | 0·01<br>(0·01;0·02) | 7·05<br>(5·07; 9·70) | 194·67<br>(138·35; 271·19) | 581·93<br>(472·02;712·03) | 3·07<br>(2·00;4·44) | 1·0688<br>(0·7728; 1·4484) | 32·09<br>(23·07;43·72) |
| | **2019** | 3·59<br>(2·93;4·40) | 0·01<br>(0·01;0·02) | 6·99<br>(4·89; 9·59) | 182·87<br>(127·81; 252·29) | 577·11<br>(469·75;718·40) | 2·70<br>(1·73;3·92) | 0·8981<br>(0·6393; 1·2254) | 27·44<br>(19·45;37·73) |
| | **Change (%) 2010-2019** | 12·09<br>(3·66;20·87) | -10·00<br>(-18·94;0·50) | -0·77<br>(-13·75;14·23) | -6·06<br>(-18·47;7·83) | -0·83<br>(-7·69;5·97) | -12·02<br>(-20·19;-2·71) | -15·97<br>(-26·27;-4·46) | -14·48<br>(-25·20;-2·67) |
| **Monaco** | **2010** | 0·39<br>(0·32;0·48) | 0·00<br>(0·00;0·00) G | 1·31<br>(0·90; 1·80) | 32·41<br>(22·06; 45·20) | 707·08<br>(581·64;866·62) | 5·08<br>(3·19;7·61) | 1·8017<br>(1·2537; 2·4670) | 54·77<br>(36·99;77·68) |
| | **2019** | 0·42<br>(0·34;0·51) | 0·00<br>(0·00;0·00) H | 1·30(0·86; 1·85) | 31·45(20·82;<br>46·43) | 702·95<br>(574·64;862·42) | 4·90<br>(3·08;7·36) | 1·6481(1·0887;<br>2·4108) | 50·18<br>(32·84;73·82) |
| | **Change (%) 2010-2019** | 6·69<br>(0·21;14·44) | -10·90<br>(-19·67;-0·77) | -1·29<br>(-21·39;21·18) | -2·95<br>(-23·69;21·59) | -0·58<br>(-6·12;5·74) | -3·45<br>(-11·62;4·85) | -8·52<br>(-27·61;12·56) | -8·37<br>(-27·98;16·23) |
| **Netherlands** | **2010** | 50·02<br>(40·97;60·12) | 0·61<br>(0·39;0·90) | 268·45<br>(190·14;366·85) | 5953·50<br>(4292·74; 8214·88) | 229·66<br>(188·92;277·40) | 3·06<br>(1·99;4·46) | 0·9795<br>(0·7112; 1·3185) | 23·63<br>(17·20;32·43) |
| | **2019** | 82·65<br>(67·91;101·04) | 0·51<br>(0·33;0·75) | 290·56<br>(201·84;399·18) | 5932·63<br>(4274·91; 8170·02) | 338·73<br>(276·55;417·94) | 2·76<br>(1·78;4·04) | 0·8808<br>(0·6230; 1·1789) | 20·84<br>(15·22;28·71) |
| | **Change (%) 2010-2019** | 65·24<br>(51·90;79·94) | -15·53<br>(-25·48;-4·85) | 8·23<br>(-3·08;21·27) | -0·35<br>(-10·62;11·07) | 47·49<br>(34·80;60·06) | -9·94<br>(-20·02;0·34) | -10·08<br>(-18·45;-0·33) | -11·80<br>(-19·68;-3·03) |
| **Norway** | **2010** | 42·11<br>(34·09;51·66) | 0·18<br>(0·13;0·24) | 58·06<br>(48·64; 69·86) | 1446·87<br>(1227·17; 1710·31) | 691·67<br>(555·74;856·44) | 3·32<br>(2·43;4·34) | 0·7507<br>(0·6371; 0·8912) | 21·00<br>(17·87;24·80) |
| | **2019** | 48·72<br>(39·43;59·83) | 0·16<br>(0·11;0·21) | 60·29<br>(49·68; 73·50) | 1423·42<br>(1201·76; 1705·41) | 703·19<br>(565·42;871·47) | 2·66<br>(1·82;3·63) | 0·6617<br>(0·5527; 0·7976) | 17·92<br>(15·16;21·40) |

| | | | | | | | | | |
|---|---|---|---|---|---|---|---|---|---|
| | **Change (%) 2010-2019** | 15·70 (14·28;17·26) | -14·45 (-28·35;-1·80) | 3·85 (-1·94;10·90) | -1·62 (-7·60;5·69) | 1·67 (0·49;2·92) | -20·06 (-32·56;-9·18) | -11·85 (-16·94;-5·60) | -14·66 (-20·00;-8·32) |
| **Portugal** | **2010** | 99·27 (81·17;120·54) | 0·63 (0·39;0·94) | 399·35 (290·63;544·92) | 11409·67 (8282·95; 15571·56) | 683·13 (553·41;840·25) | 4·98 (3·15;7·36) | 2·3050 (1·6927; 3·1282) | 73·24 (52·94;99·82) |
| | **2019** | 102·27 (84·04;123·90) | 0·48 (0·30;0·73) | 363·59 (263·11;494·61) | 9565·27 (6765·35; 13396·00) | 656·27 (536·22;806·93) | 3·92 (2·47;5·87) | 1·8265 (1·3172; 2·5258) | 56·10 (40·46;78·53) |
| | **Change (%) 2010-2019** | 3·02 (-4·17;10·41) | -22·96 (-31·59;-12·59) | -8·95 (-17·95;0·63) | -16·17 (-24·57;-7·42) | -3·93 (-10·14;2·47) | -21·29 (-29·45;-11·68) | -20·76 (-28·43;-13·10) | -23·41 (-31·02;-15·41) |
| **San Marino** | **2010** | 0·25 (0·21;0·31) | 0·00 (0·00;0·00) I | 1·23 (0·76; 1·82) | 25·41 (15·97; 38·22) | 625·04 (508·86;777·80) | 7·07 (4·44;10·54) | 2·2414 (1·3936; 3·3322) | 54·95 (34·20;83·73) |
| | **2019** | 0·28 (0·23;0·35) | 0·00 (0·00;0·00) L | 1·41 (0·84; 2·19) | 28·74 (16·61; 45·59) | 625·98 (510·04;768·35) | 6·73 (4·26;10·17) | 2·1803 (1·2709; 3·4449) | 54·14 (30·38;86·72) |
| | **Change (%) 2010-2019** | 12·48 (7·19;17·94) | -4·20 (-13·98;5·29) | 14·91 (-15·19;52·53) | 13·12 (-18·44;54·21) | 0·15 (-4·67;5·24) | -4·82 (-13·24;3·95) | -2·73 (-28·96;30·70) | -1·47 (-29·09;35·23) |
| **Spain** | **2010** | 454·05 (366·21;553·77) | 4·80 (3·29;6·41) | 2151·14 (1619·60;2787·59) | 52273·42 (40482·59; 66741·85) | 723·17 (583·64;889·13) | 8·00 (5·56;10·67) | 2·7524 (2·1008; 3·5647) | 75·15 (57·12;97·24) |
| | **2019** | 448·86 (363·37;544·26) | 4·00 (2·71;5·49) | 2076·77 (1548·31;2699·33) | 46479·76 (35862·86; 59762·60) | 672·92 (547·20;825·97) | 6·75 (4·64;9·14) | 2·2533 (1·7255; 2·8967) | 59·44 (45·25;76·83) |
| | **Change (%) 2010-2019** | -1·14 (-10·07;8·52) | -16·74 (-25·31;-7·58) | -3·46 (-12·21;6·28) | -11·08 (-19·00;-2·94) | -6·95 (-14·12;0·97) | -15·64 (-23·33;-7·91) | -18·14 (-24·85;-11·01) | -20·90 (-27·72;-14·07) |
| **Sweden** | **2010** | 72·41 (58·41;89·18) | 0·29 (0·20;0·39) | 155·67 (130·69;183·83) | 3636·50 (3049·25; 4370·76) | 567·83 (460·04;701·13) | 2·76 (1·93;3·70) | 0·9201 (0·7791; 1·0964) | 24·75 (20·68;29·53) |
| | **2019** | 79·98 (64·52;98·17) | 0·28 (0·18;0·38) | 164·22 (134·98;196·82) | 3644·91 (3009·68; 4416·80) | 566·96 (456·53;699·23) | 2·54 (1·71;3·52) | 0·8564 (0·7103; 1·0313) | 22·37 (18·56;27·00) |
| | **Change (%) 2010-2019** | 10·45 (7·48;13·76) | -4·27 (-13·51;4·48) | 5·49 (-3·29;14·38) | 0·23 (-7·95;8·63) | -0·15 (-2·84;2·73) | -8·14 (-16·89;-0·05) | -6·92 (-14·59;1·08) | -9·63 (-16·67;-2·06) |
| **Switzerland** | **2010** | 65·99 (53·65;81·42) | 0·37 (0·23;0·55) | 168·10 (120·53;229·46) | 4160·75 (2970·60; 5764·67) | 618·96 (498·67;759·94) | 3·69 (2·35;5·37) | 1·2402 (0·9003; 1·7018) | 34·10 (24·23;47·01) |
| | **2019** | 76·19 (61·95;92·73) | 0·33 (0·21;0·49) | 169·70 (118·08;231·78) | 3937·47 (2831·37; 5532·25) | 625·29 (507·21;765·07) | 3·23 (2·07;4·80) | 1·0375 (0·7449; 1·4291) | 27·78 (19·73;38·76) |
| | **Change (%) 2010-2019** | 15·46 (7·08;23·88) | -11·20 (-21·48;-0·11) | 0·96 (-8·85;11·76) | -5·37 (-14·31;4·17) | 1·02 (-5·47;8·06) | -12·30 (-22·39;-1·87) | -16·34 (-23·83;-8·01) | -18·52 (-25·92;-10·40) |
| **United Kingdom** | **2010** | 1108·29 (906·45;1359·91) | 1·29 (0·97;1·68) | 745·91 (616·98;909·67) | 22965·61 (18767·66; 28213·61) | 1386·43 (1120·17;1711·74) | 1·88 (1·40;2·40) | 0·8108 (0·6695; 0·9935) | 27·35 (22·37;33·55) |
| | **2019** | 716·74 (582·86;873·12) | 1·26 (0·92;1·67) | 786·72 (646·07;967·07) | 23132·80 (18862·71; 28379·12) | 815·28 (660·06;1004·45) | 1·83 (1·33;2·42) | 0·7688 (0·6336; 0·9371) | 25·47 (20·79;31·31) |
| | **Change (%) 2010-2019** | -35·33 (-36·83;-33·72) | -2·25 (-10·90;7·27) | 5·47 (1·43;9·33) | 0·73 (-3·16;4·45) | -41·20 (-42·43;-40·11) | -2·31 (-10·39;6·14) | -5·19 (-8·32;-2·23) | -6·88 (-9·84;-4·15) |
| **Liver cancer due to hepatitis** | | | | | | | | | |
| **Andorra** | **2010** | 0·01 (0·00;0·01) | 0·00 (0·00;0·01) | 4·00 (2·72; 5·58) | 76·26 (50·90; 109·18) | 5·07 (3·37;7·37) | 3·56 (2·37;5·07) | 3·2573 (2·2108;4·5745) | 63·95 (42·53;92·49) |
| | **2019** | 0·01 (0·00;0·01) | 0·01 (0·00;0·01) | 5·07 (3·43; 7·07) | 94·98 (61·37; 135·58) | 5·52 (3·56;7·88) | 3·84 (2·50;5·45) | 3·5121 (2·3268;4·9667) | 67·87 (43·96;97·65) |

| | | | | | | | | | |
|---|---|---|---|---|---|---|---|---|---|
| | **Change (%) 2010-2019** | 25·96 (-2·24;64·08) | 25·67 (-1·75;61·30) | 26·73 (-0·34;62·21) | 24·55 (-3·03;60·33) | 8·92 (-15·45;42·48) | 7·74 (-16·36;38·71) | 7·82 (-16·00;38·32) | 6·13 (-17·86;37·68) |
| **Austria** | **2010** | 0·38 (0·28;0·50) | 0·27 (0·20;0·35) | 237·21 (174·82;306·47) | 4404·75 (3160·84; 5823·46) | 2·51 (1·81;3·32) | 1·70 (1·24;2·23) | 1·4755 (1·0840;1·9247) | 29·89 (20·96;39·69) |
| | **2019** | 0·42 (0·29;0·61) | 0·29 (0·20;0·41) | 256·08 (186·57;342·44) | 4658·61 (3306·06; 6390·02) | 2·45 (1·66;3·53) | 1·60 (1·10;2·30) | 1·3690 (0·9939;1·8466) | 27·43 (19·17;37·62) |
| | **Change (%) 2010-2019** | 11·68 (-12·07;40·58) | 8·39 (-13·64;35·09) | 7·95 (-3·97;21·23) | 5·76 (-6·43;20·28) | -2·38 (-23·72;23·20) | -6·11 (-25·92;17·74) | -7·22 (-17·58;4·77) | -8·22 (-19·43;4·25) |
| **Belgium** | **2010** | 0·34 (0·25;0·43) | 0·28 (0·21;0·35) | 275·45 (207·26;345·79) | 5001·86 (3749·90; 6322·23) | 1·75 (1·29;2·23) | 1·36 (1·02;1·72) | 1·3046 (0·9884;1·6457) | 26·17 (19·36;33·40) |
| | **2019** | 0·45 (0·31;0·63) | 0·36 (0·25;0·48) | 348·84 (265·05;437·57) | 6026·99 (4514·01; 7721·51) | 2·04 (1·40;2·90) | 1·51 (1·05;2·08) | 1·4233 (1·0722;1·8050) | 27·67 (20·67;35·93) |
| | **Change (%) 2010-2019** | 32·27 (2·89;67·97) | 27·59 (0·26;59·77) | 26·65 (14·67;40·35) | 20·49 (8·87;33·79) | 16·95 (-9·64;50·23) | 11·17 (-12·96;40·64) | 9·10 (-1·36;20·72) | 5·71 (-4·69;17·55) |
| **Cyprus** | **2010** | 0·02 (0·02;0·03) | 0·02 (0·01;0·02) | 18·82 (13·95; 24·01) | 354·57 (260·16; 461·12) | 1·73 (1·30;2·19) | 1·45 (1·09;1·82) | 1·4648 (1·1237;1·8332) | 25·48 (18·92;32·69) |
| | **2019** | 0·03 (0·02;0·05) | 0·03 (0·02;0·03) | 25·72 (18·61; 33·03) | 469·61 (334·56; 608·61) | 1·80 (1·30;2·32) | 1·41 (1·04;1·82) | 1·4030 (1·0478;1·7866) | 24·60 (17·90;31·74) |
| | **Change (%) 2010-2019** | 43·32 (18·52;69·54) | 37·77 (15·40;62·48) | 36·64 (14·70;60·14) | 32·45 (11·73;55·71) | 3·84 (-13·20;22·77) | -2·27 (-18·12;15·08) | -4·22 (-18·16;12·38) | -3·47 (-18·33;13·39) |
| **Denmark** | **2010** | 0·18 (0·13;0·24) | 0·13 (0·09;0·16) | 112·85 (83·28;143·91) | 2200·07 (1609·58; 2879·77) | 1·90 (1·39;2·51) | 1·30 (0·96;1·69) | 1·1412 (0·8428;1·4585) | 23·53 (17·15;30·65) |
| | **2019** | 0·22 (0·14;0·31) | 0·15 (0·10;0·22) | 136·52 (100·28;177·48) | 2575·49 (1860·63; 3422·97) | 1·98 (1·31;2·83) | 1·32 (0·90;1·86) | 1·1512 (0·8537;1·4904) | 23·58 (16·90;31·67) |
| | **Change (%) 2010-2019** | 21·82 (-7·67;57·53) | 20·76 (-6·29;54·59) | 20·98 (7·42;34·92) | 17·06 (2·52;30·78) | 4·01 (-20·21;34·68) | 1·54 (-21·09;29·79) | 0·88 (-10·08;12·50) | 0·21 (-11·63;12·33) |
| **Finland** | **2010** | 0·36 (0·27;0·46) | 0·20 (0·15;0·26) | 161·57 (120·69;205·76) | 2841·15 (2094·99; 3636·37) | 3·41 (2·58;4·34) | 1·91 (1·44;2·42) | 1·4844 (1·1105;1·8906) | 28·03 (20·83;35·73) |
| | **2019** | 0·46 (0·31;0·65) | 0·26 (0·18;0·36) | 202·86 (151·93;257·28) | 3371·51 (2457·71; 4413·66) | 3·72 (2·53;5·25) | 2·00 (1·39;2·76) | 1·5219 (1·1276;1·9435) | 28·19 (20·69;36·83) |
| | **Change (%) 2010-2019** | 28·90 (-1·86;67·30) | 26·10 (-0·90;60·70) | 25·55 (11·99;40·21) | 18·67 (5·22;33·01) | 8·89 (-17·57;41·80) | 4·57 (-17·87;34·07) | 2·53 (-8·73;13·88) | 0·54 (-10·67;12·33) |
| **France** | **2010** | 4·34 (3·36;5·47) | 3·39 (2·63;4·19) | 3245·52 (2514·89;3990·82) | 61606·53 (47092·30; 76942·87) | 3·97 (3·02;5·04) | 2·94 (2·27;3·67) | 2·7157 (2·1005;3·3530) | 57·16 (43·29;72·29) |
| | **2019** | 4·79 (3·27;6·66) | 3·64 (2·54;5·00) | 3503·58 (2648·58;4375·37) | 63012·80 (46685·65; 80863·97) | 3·80 (2·58;5·30) | 2·68 (1·84;3·71) | 2·4494 (1·8367;3·1096) | 50·31 (36·98;65·29) |
| | **Change (%) 2010-2019** | 10·33 (-17·17;44·96) | 7·57 (-16·60;38·73) | 7·95 (-4·88;21·51) | 2·28 (-11·37;16·15) | -4·36 (-29·25;27·04) | -8·68 (-30·61;19·39) | -9·81 (-21·31;1·89) | -11·98 (-23·97;1·05) |
| **Germany** | **2010** | 3·37 (2·56;4·29) | 2·36 (1·80;3·00) | 2111·76 (1608·16;2647·70) | 36525·97 (27641·08; 46390·65) | 1·99 (1·52;2·56) | 1·34 (1·03;1·70) | 1·1696 (0·9005;1·4709) | 22·16 (16·79;28·35) |
| | **2019** | 4·02 (2·70;5·63) | 2·73 (1·84;3·79) | 2413·11 (1783·90;3079·27) | 41231·22 (30215·22; 53409·55) | 2·14 (1·45;3·00) | 1·37 (0·93;1·90) | 1·1660 (0·8661;1·4917) | 22·56 (16·38;30·02) |
| | **Change (%)** | 19·33 | 15·57 | 14·27 | 12·88 | 7·18 | 2·28 | -0·31 | 1·79 |

| | | | | | | | | | |
|---|---|---|---|---|---|---|---|---|---|
| | **2010-2019** | (-8·71;53·08) | (-10·21;47·54) | (1·64;26·61) | (0·65;25·99) | (-18·59;38·79) | (-21·16;31·25) | (-10·98;10·49) | (-9·27;13·14) |
| **Greece** | **2010** | 0·15<br>(0·10;0·20) | 0·13<br>(0·09;0·17) | 129·50<br>(89·11;175·80) | 2016·58<br>(1383·31; 2756·64) | 0·66<br>(0·46;0·88) | 0·55<br>(0·39;0·73) | 0·5515<br>(0·3908;0·7352) | 9·24<br>(6·43;12·56) |
| | **2019** | 0·18<br>(0·11;0·26) | 0·16<br>(0·10;0·23) | 165·61<br>(115·30;226·59) | 2424·74<br>(1670·51; 3366·57) | 0·71<br>(0·46;1·06) | 0·59<br>(0·38;0·86) | 0·5850<br>(0·4081;0·8007) | 10·11<br>(6·93;14·17) |
| | **Change (%) 2010-2019** | 19·89<br>(-7·34;52·51) | 24·44<br>(-2·14;56·73) | 27·89<br>(12·50;46·31) | 20·24<br>(7·16;35·03) | 8·04<br>(-16·84;37·78) | 6·99<br>(-15·55;35·14) | 6·08<br>(-5·41;18·65) | 9·40<br>(-2·01;22·23) |
| **Iceland** | **2010** | 0·01<br>(0·01;0·01) | 0·01<br>(0·00;0·01) | 4·62<br>(3·43; 5·83) | 85·21<br>(62·04; 109·97) | 1·68<br>(1·23;2·18) | 1·13<br>(0·83;1·45) | 1·0159<br>(0·7447;1·2972) | 19·84<br>(14·47;25·69) |
| | **2019** | 0·01<br>(0·01;0·01) | 0·01<br>(0·00;0·01) | 5·96<br>(4·40; 7·73) | 108·78<br>(77·96; 145·64) | 1·78<br>(1·26;2·38) | 1·20<br>(0·87;1·59) | 1·0293<br>(0·7597;1·3533) | 20·24<br>(14·40;27·11) |
| | **Change (%) 2010-2019** | 31·21<br>(9·62;57·84) | 33·12<br>(12·90;57·61) | 29·12<br>(13·20;47·36) | 27·66<br>(9·97;47·21) | 5·96<br>(-12·13;27·02) | 5·68<br>(-10·90;25·26) | 1·32<br>(-11·54;16·18) | 2·00<br>(-12·06;17·75) |
| **Ireland** | **2010** | 0·11<br>(0·08;0·14) | 0·08<br>(0·06;0·10) | 76·77<br>(58·83; 96·73) | 1425·03<br>(1073·56; 1815·02) | 1·84<br>(1·39;2·33) | 1·37<br>(1·03;1·73) | 1·2987<br>(0·9951;1·6383) | 24·56<br>(18·35;31·31) |
| | **2019** | 0·16<br>(0·10;0·22) | 0·11<br>(0·08;0·15) | 101·10<br>(75·76;129·39) | 1822·41<br>(1335·05; 2362·97) | 2·09<br>(1·40;2·93) | 1·46<br>(0·99;2·03) | 1·3137<br>(0·9857;1·6839) | 24·53<br>(17·95;31·62) |
| | **Change (%) 2010-2019** | 45·02<br>(10·39;84·99) | 37·69<br>(5·74;74·68) | 31·70<br>(17·78;47·59) | 27·89<br>(14·08;43·57) | 13·77<br>(-13·58;45·64) | 6·56<br>(-17·93;35·35) | 1·16<br>(-9·14;12·74) | -0·14<br>(-10·62;11·60) |
| **Israel** | **2010** | 0·13<br>(0·10;0·16) | 0·11<br>(0·09;0·14) | 115·11<br>(90·28;140·66) | 2162·72<br>(1648·47; 2665·88) | 1·47<br>(1·11;1·82) | 1·25<br>(0·96;1·54) | 1·2726<br>(0·9924;1·5626) | 24·86<br>(18·76;30·74) |
| | **2019** | 0·18<br>(0·12;0·24) | 0·15<br>(0·11;0·20) | 149·43<br>(116·32;185·31) | 2760·98<br>(2071·54; 3447·94) | 1·55<br>(1·08;2·12) | 1·27<br>(0·90;1·74) | 1·2542<br>(0·9657;1·5624) | 24·46<br>(18·36;30·74) |
| | **Change (%) 2010-2019** | 36·41<br>(5·04;74·75) | 32·25<br>(3·23;67·88) | 29·82<br>(18·04;42·73) | 27·66<br>(15·29;40·66) | 5·47<br>(-19·13;35·63) | 1·18<br>(-21·88;28·76) | -1·45<br>(-10·26;8·33) | -1·58<br>(-11·06;8·18) |
| **Italy** | **2010** | 6·03<br>(5·40;6·65) | 4·05<br>(3·63;4·40) | 3673·44<br>(3281·79;3992·30) | 66265·35<br>(60126·26; 71953·06) | 5·27<br>(4·68;5·87) | 3·23<br>(2·93;3·52) | 2·7703<br>(2·5007;2·9977) | 56·00<br>(50·89;61·00) |
| | **2019** | 6·62<br>(5·21;8·38) | 4·44<br>(3·57;5·46) | 4032·14<br>(3535·40;4451·72) | 71136·44<br>(62706·62; 78794·53) | 5·20<br>(4·05;6·65) | 3·14<br>(2·50;3·92) | 2·6610<br>(2·3395;2·9328) | 53·90<br>(47·26;60·26) |
| | **Change (%) 2010-2019** | 9·85<br>(-10·40;33·52) | 9·51<br>(-8·88;30·94) | 9·76<br>(2·29;16·07) | 7·35<br>(-0·27;13·78) | -1·20<br>(-20·27;22·07) | -2·91<br>(-19·94;17·63) | -3·95<br>(-10·61;1·63) | -3·75<br>(-10·88;2·32) |
| **Luxembourg** | **2010** | 0·02<br>(0·01;0·02) | 0·01<br>(0·01;0·02) | 12·39<br>(9·20; 15·81) | 225·52<br>(166·58; 292·19) | 2·22<br>(1·63;2·87) | 1·64<br>(1·23;2·11) | 1·5396<br>(1·1518;1·9636) | 29·59<br>(21·82;38·56) |
| | **2019** | 0·02<br>(0·01;0·03) | 0·01<br>(0·01;0·02) | 14·38<br>(10·29; 19·65) | 255·63<br>(178·17; 354·07) | 1·99<br>(1·35;2·86) | 1·43<br>(0·98;2·06) | 1·3729<br>(0·9760;1·8767) | 25·97<br>(17·93;36·26) |
| | **Change (%) 2010-2019** | 15·03<br>(-9·43;49·73) | 12·52<br>(-11·93;44·05) | 16·10<br>(-4·53;39·45) | 13·35<br>(-6·36;37·15) | -10·32<br>(-29·97;17·27) | -12·90<br>(-31·49;12·58) | -10·82<br>(-26·51;6·91) | -12·23<br>(-28·06;6·01) |
| **Malta** | **2010** | 0·01<br>(0·01;0·01) | 0·01<br>(0·01;0·01) | 6·70<br>(4·98; 8·65) | 128·83<br>(93·67; 169·20) | 1·12<br>(0·84;1·46) | 0·92<br>(0·69;1·19) | 0·9071<br>(0·6781;1·1663) | 17·83<br>(13·00;23·28) |
| | **2019** | 0·01<br>(0·01;0·01) | 0·01<br>(0·01;0·01) | 8·29<br>(6·08; 10·73) | 150·50<br>(107·95; 200·42) | 1·17<br>(0·82;1·59) | 0·91<br>(0·66;1·22) | 0·8538<br>(0·6321;1·1035) | 16·79<br>(12·15;22·52) |
| | **Change (%) 2010-2019** | 29·69<br>(7·36;59·00) | 27·29<br>(6·54;54·70) | 23·78<br>(7·32;43·79) | 16·82<br>(0·23;36·19) | 4·29<br>(-13·56;26·61) | -1·15<br>(-17·09;19·27) | -5·87<br>(-17·93;8·72) | -5·83<br>(-18·77;8·42) |

| | | | | | | | | | |
|---|---|---|---|---|---|---|---|---|---|
| **Monaco** | **2010** | 0·00 (0·00;0·01) | 0·00 (0·00;0·00) M | 2·91 (2·08; 3·81) | 49·80 (34·97; 66·94) | 4·70 (3·22;6·32) | 3·42 (2·41;4·58) | 3·1066 (2·2237;4·1349) | 61·35 (41·99;83·14) |
| | **2019** | 0·00 (0·00;0·01) | 0·00 (0·00;0·00) N | 2·99 (2·12; 3·96) | 51·69 (35·27; 70·35) | 4·63 (3·17;6·32) | 3·28 (2·27;4·41) | 2·9291 (2·0620;3·9321) | 57·84 (39·39;79·22) |
| | **Change (%) 2010-2019** | 6·95 (-15·06;34·77) | 4·19 (-17·57;29·07) | 2·56 (-18·05;26·12) | 3·80 (-18·28;29·43) | -1·39 (-22·72;25·60) | -4·13 (-24·48;20·13) | -5·71 (-24·71;16·90) | -5·72 (-26·11;19·08) |
| **Netherlands** | **2010** | 0·33 (0·25;0·43) | 0·25 (0·18;0·31) | 234·41 (174·07;296·29) | 4171·72 (3067·11; 5339·14) | 1·21 (0·91;1·56) | 0·87 (0·65;1·10) | 0·8066 (0·6003;1·0168) | 15·12 (11·20;19·40) |
| | **2019** | 0·49 (0·33;0·69) | 0·36 (0·25;0·49) | 329·58 (243·83;417·96) | 5712·25 (4178·22; 7424·33) | 1·50 (1·02;2·08) | 1·03 (0·71;1·43) | 0·9209 (0·6854;1·1727) | 17·34 (12·61;22·47) |
| | **Change (%) 2010-2019** | 46·62 (12·79;84·63) | 43·18 (12·16;78·80) | 40·60 (28·01;55·31) | 36·93 (24·00;51·67) | 23·93 (-4·85;57·55) | 18·00 (-7·87;47·14) | 14·17 (4·33;26·12) | 14·63 (3·97;27·14) |
| **Norway** | **2010** | 0·11 (0·10;0·13) | 0·08 (0·07;0·09) | 80·28 (71·20; 89·45) | 1462·72 (1293·18; 1638·36) | 1·48 (1·28;1·68) | 1·04 (0·92;1·17) | 0·9471 (0·8371;1·0559) | 19·02 (16·75;21·38) |
| | **2019** | 0·14 (0·11;0·18) | 0·10 (0·08;0·13) | 98·32 (84·16;112·69) | 1793·09 (1535·54; 2083·32) | 1·58 (1·26;1·96) | 1·08 (0·87;1·32) | 0·9870 (0·8466;1·1339) | 19·63 (16·72;22·86) |
| | **Change (%) 2010-2019** | 26·55 (6·21;50·64) | 22·65 (4·11;43·77) | 22·48 (12·73;33·84) | 22·59 (12·02;35·46) | 7·17 (-10·48;27·73) | 3·97 (-11·75;21·93) | 4·22 (-4·38;13·98) | 3·22 (-5·90;14·31) |
| **Portugal** | **2010** | 0·33 (0·24;0·42) | 0·30 (0·22;0·38) | 315·77 (235·81;399·76) | 6136·02(4513·85; 7857·01) | 1·69 (1·24;2·17) | 1·48 (1·10;1·88) | 1·5050 (1·1267;1·8926) | 32·22 (23·59;41·45) |
| | **2019** | 0·43 (0·29;0·60) | 0·39 (0·27;0·54) | 404·36 (301·70;512·15) | 7535·92 (5439·45; 9827·54) | 1·97 (1·31;2·80) | 1·64 (1·11;2·29) | 1·6339 (1·2064;2·0879) | 34·82 (24·71;46·34) |
| | **Change (%) 2010-2019** | 32·16 (1·69;70·28) | 28·83 (0·16;64·94) | 28·05 (15·94;41·61) | 22·81 (10·67;35·99) | 16·38 (-11·02;51·11) | 10·98 (-14·73;42·93) | 8·56 (-2·17;19·95) | 8·07 (-3·37;20·32) |
| **San Marino** | **2010** | 0·00 (0·00;0·00) O | 0·00 (0·00;0·00) P | 0·56 (0·35; 0·83) | 9·73 (5·89; 14·71) | 1·39 (0·91;1·99) | 1·00 (0·67;1·44) | 0·9641 (0·6027;1·4343) | 18·69 (11·23;28·38) |
| | **2019** | 0·00 (0·00;0·00) Q | 0·00 (0·00;0·00) R | 0·69 (0·43; 1·04) | 11·93 (7·04; 18·29) | 1·55 (1·03;2·24) | 1·08 (0·73;1·55) | 1·0047 (0·6069;1·5300) | 19·65 (11·40;30·97) |
| | **Change (%) 2010-2019** | 29·03 (1·24;65·92) | 26·07 (0·19;60·74) | 22·85 (-10·08;69·64) | 22·66 (-11·71;71·56) | 11·25 (-13·10;43·32) | 7·65 (-15·13;37·05) | 4·21 (-23·81;44·26) | 5·14 (-24·91;47·85) |
| **Spain** | **2010** | 3·60 (2·84;4·38) | 2·60 (2·08;3·10) | 2436·28 (1967·71;2893·88) | 44848·56 (35375·10; 53831·55) | 4·64 (3·60;5·75) | 3·11 (2·47;3·75) | 2·7779 (2·2305;3·3252) | 57·06 (43·80;69·18) |
| | **2019** | 3·80 (2·67;5·24) | 2·84 (2·02;3·75) | 2735·49 (2182·35;3275·18) | 48902·21 (37654·84; 60372·65) | 4·31 (2·99;5·92) | 2·97 (2·07;3·98) | 2·7020 (2·1019;3·3022) | 54·97 (41·91;68·27) |
| | **Change (%) 2010-2019** | 5·53 (-18·24;36·57) | 9·26 (-13·99;38·78) | 12·28 (2·84;23·12) | 9·04 (-1·12;19·77) | -7·06 (-29·53;20·88) | -4·59 (-25·85;22·00) | -2·73 (-11·17;6·67) | -3·67 (-13·65;6·29) |
| **Sweden** | **2010** | 0·17 (0·14;0·20) | 0·16 (0·14;0·19) | 179·31 (152·59;208·30) | 3291·62 (2740·84; 3889·86) | 0·98 (0·81;1·16) | 0·89 (0·75;1·05) | 0·9467 (0·7978;1·1114) | 19·51 (16·08;23·30) |
| | **2019** | 0·23 (0·18;0·28) | 0·22 (0·17;0·27) | 236·72 (198·07;276·79) | 4224·83 (3499·55; 4953·87) | 1·16 (0·90;1·46) | 1·04 (0·82;1·28) | 1·0825 (0·9072;1·2665) | 21·96 (18·13;26·20) |
| | **Change (%) 2010-2019** | 34·44 (13·87;56·97) | 33·63 (13·80;55·08) | 32·02 (21·51;42·03) | 28·35 (17·67;38·94) | 18·52 (0·33;38·77) | 16·40 (-1·05;35·65) | 14·34 (5·44;22·93) | 12·59 (3·24;22·18) |
| **Switzerland** | **2010** | 0·44 | 0·28 | 235·81 | 4428·73 | 3·20 | 1·93 | 1·5855 | 32·46 |

| | | | | | | | | | |
|---|---|---|---|---|---|---|---|---|---|
| | | (0·32;0·58) | (0·21;0·36) | (175·87;299·64) | (3235·73; 5771·85) | (2·30;4·25) | (1·41;2·50) | (1·1746;2·0327) | (23·43;42·78) |
| | **2019** | 0·55 (0·36;0·79) | 0·34 (0·23;0·48) | 288·77 (211·17;377·33) | 5192·98 (3698·17; 6882·14) | 3·26 (2·11;4·76) | 1·93 (1·29;2·73) | 1·5802 (1·1477;2·0772) | 31·38 (22·07;42·23) |
| | **Change (%) 2010-2019** | 23·60 (-6·52;63·21) | 22·29 (-4·46;60·21) | 22·46 (10·01;36·80) | 17·26 (4·33;31·39) | 1·88 (-23·69;35·23) | 0·12 (-22·65;30·90) | -0·33 (-11·10;11·04) | -3·34 (-14·27;8·68) |
| **United Kingdom** | **2010** | 2·53 (2·25;2·83) | 1·74 (1·54;1·94) | 1569·13 (1377·66;1745·88) | 27049·22 (23966·66; 30273·30) | 2·32 (2·06;2·61) | 1·53 (1·36;1·71) | 1·3425 (1·1885;1·4919) | 25·42 (22·50;28·51) |
| | **2019** | 3·48 (2·82;4·25) | 2·34 (1·91;2·84) | 2086·86 (1830·65;2334·86) | 35044·46 (30905·96; 39629·97) | 2·78 (2·25;3·40) | 1·79 (1·46;2·17) | 1·5456 (1·3644;1·7309) | 28·67 (25·18;32·52) |
| | **Change (%) 2010-2019** | 37·77 (15·83;61·99) | 34·41 (13·64;57·57) | 32·99 (27·52;38·65) | 29·56 (24·32;34·89) | 20·02 (0·17;41·61) | 16·54 (-1·83;37·15) | 15·13 (10·37;19·83) | 12·78 (8·13;17·56) |

UI= Uncertainty Interval

A: 2·38(1·99;2·85); B: 2·57(2·10;3·09); C: 1·57(1·32;1·87); D: 1·76(1·47;2·09); E: 3·23(2·00;4·86); F: 3·15(1·88;4·88); G: 2·04(1·25;3·09); H: 1·81(1·12;2·76); I: 2·60(1·61;3·94); L: 2·49(1·55;3·75); M: 3·04(2·16;4·03); N: 3·17(2·24;4·24); O: 0·73(0·49;1·04); P: 0·56(0·38;0·80); Q: 0·94(0·64;1·35); R: 0·71(0·48;1·02)
